# Expansion of the phosphatidylethanolamine binding protein family in legumes: a case study of *Lupinus angustifolius* L. *FLOWERING LOCUS T* homologs, *LanFTc1* and *LanFTc2*

**DOI:** 10.1186/s12864-016-3150-z

**Published:** 2016-10-21

**Authors:** Michał Książkiewicz, Sandra Rychel, Matthew N. Nelson, Katarzyna Wyrwa, Barbara Naganowska, Bogdan Wolko

**Affiliations:** 1 Institute of Plant Genetics of the Polish Academy of Sciences, Strzeszyńska 34, 60-479 Poznań, Poland; 2 Natural Capital and Plant Health, Royal Botanic Gardens Kew, Wakehurst Place, Ardingly, West Sussex, RH17 6TN UK; 3 School of Plant Biology, The University of Western Australia, 35 Stirling Highway, Crawley, WA 6009 Australia; 4 The UWA Institute of Agriculture, The University of Western Australia, 35 Stirling Highway, Crawley, WA 6009 Australia

**Keywords:** *Lupinus angustifolius*, Genomics, DNA sequencing, Flowering locus T, Phosphatidylethanolamine binding protein, Synteny, Duplication, BAC-FISH

## Abstract

**Background:**

The *Arabidopsis FLOWERING LOCUS T* (*FT*) gene, a member of the phosphatidylethanolamine binding protein (PEBP) family, is a major controller of flowering in response to photoperiod, vernalization and light quality. In legumes, *FT* evolved into three, functionally diversified clades, *FTa*, *FTb* and *FTc*. A milestone achievement in narrow-leafed lupin (*Lupinus angustifolius* L.) domestication was the loss of vernalization responsiveness at the *Ku* locus. Recently, one of two existing *L. angustifolius* homologs of *FTc*, *LanFTc1*, was revealed to be the gene underlying *Ku*. It is the first recorded involvement of an *FTc* homologue in vernalization. The evolutionary basis of this phenomenon in lupin has not yet been deciphered.

**Results:**

Bacterial artificial chromosome (BAC) clones carrying *LanFTc1* and *LanFTc2* genes were localized in different mitotic chromosomes and constituted sequence-specific landmarks for linkage groups NLL-10 and NLL-17. BAC-derived superscaffolds containing *LanFTc* genes revealed clear microsyntenic patterns to genome sequences of nine legume species. Superscaffold-1 carrying *LanFTc1* aligned to regions encoding one or more *FT*-like genes whereas superscaffold-2 mapped to a region lacking such a homolog. Comparative mapping of the *L. angustifolius* genome assembly anchored to linkage map localized superscaffold-1 in the middle of a 15 cM conserved, collinear region. In contrast, superscaffold-2 was found at the edge of a 20 cM syntenic block containing highly disrupted collinearity at the *LanFTc2* locus. 118 PEBP-family full-length homologs were identified in 10 legume genomes. Bayesian phylogenetic inference provided novel evidence supporting the hypothesis that whole-genome and tandem duplications contributed to expansion of PEBP-family genes in legumes. Duplicated genes were subjected to strong purifying selection. Promoter analysis of *FT* genes revealed no statistically significant sequence similarity between duplicated copies; only RE-alpha and CCAAT-box motifs were found at conserved positions and orientations.

**Conclusions:**

Numerous lineage-specific duplications occurred during the evolution of legume PEBP-family genes. Whole-genome duplications resulted in the origin of subclades *FTa*, *FTb* and *FTc* and in the multiplication of *FTa* and *FTb* copy number. *LanFTc1* is located in the region conserved among all main lineages of Papilionoideae. *LanFTc1* is a direct descendant of ancestral *FTc*, whereas *LanFTc2* appeared by subsequent duplication.

**Electronic supplementary material:**

The online version of this article (doi:10.1186/s12864-016-3150-z) contains supplementary material, which is available to authorized users.

## Background

Transition from vegetative to reproductive growth depends on many environmental factors including photoperiod, light quality and temperature. The key control point where these external cues converge is the transcriptional regulation of *FLOWERING LOCUS T* (*FT*) gene [1]. FT belongs to phosphatidylethanolamine binding protein (PEBP) family, a very old group of proteins, widely distributed in the tree of life [2, 3]. The plant representatives of the PEBP family constitute three subfamilies, *FLOWERING LOCUS T* (*FT*)-like, *TERMINAL FLOWER1* (*TFL1*)-like, and *MOTHER OF FT AND TFL1* (*MFT*)-like [4]. FT protein was evidenced as a major component of florigen, the molecular signal that triggers the transition to flowering [5]. The promoter and intronic regions of *FT* carry all the elements that are necessary to alter *FT* expression in response to photoperiod and vernalization, and consequently, to trigger flowering [6]. In the genome of *Arabidopsis thaliana* only two homologs of this gene exist, *FT* and *TWIN SISTER OF FT* (*TSF*), and are very similar to each other (~83 % of coding sequence identity) [7]. In legumes, the number of *FT* homologs is higher, and they were grouped into three subclades *FTa*, *FTb* and *FTc* [8]. Typically, when multiple copies of the same gene appear in the genome, they acquire different functions by the processes of pseudogenization, subfunctionalization, or neo-functionalization [9, 10]. Such a phenomenon has also been observed in the legume *FT* subfamily. The *M. truncatula FTa1* gene is associated with vernalization responsiveness and early flowering, whereas *FTb* is considered to be involved in the photoperiod pathway [8, 11]. In *G. max*, a species that does not require vernalization for flowering induction, the photoperiod response is maintained by two genes classified as *FTa* and *FTc* [8, 11, 12]. In the narrow-leafed lupin genome the whole *FTb* subclade is absent and vernalization responsiveness is mediated by a gene from the *FTc* subclade [13]. *L. angustifolius* is the first legume species with its vernalization pathway anchored in the *FTc* gene and as such is a very useful model for understanding the evolution of *FT* homologs in this lineage. The uniqueness of the narrow-leafed lupin (so far) implies that phylogenetic inference based on model legumes is not representative for the genus *Lupinus* and, therefore, deciphering of evolutionary pathways require involvement genomic data from this species.

Lupins are valuable crops, appreciated as sources of protein for food and feed, as well as plants improving soil, enhancing yields and increasing economic payback for the succeeding crops in rotations. Narrow-leafed lupin (*Lupinus angustifolius* L.), as the most widely-grown lupin crop, has become the reference species for the genus *Lupinus* and more generally for the large genistoid clade. It has been the subject of cytological and molecular studies because of its relatively low chromosome number (2n = 40) and small genome size (2C = 1.89 pg), compared with other lupins [14]. Linkage maps with microsatellite-anchored fragment length polymorphisms [15], gene-based sequence tagged site (STS) markers [16] as well as consensus maps with both types of markers [17–19] were constructed. The current reference linkage map [17] contains 1475 markers of which 827 were sequenced. Bacterial artificial chromosome (BAC) libraries of the nuclear genomes were developed for two *L. angustifolius* cultivars: Polish cv. Sonet [20] and Australian cv. Tanjil [21]. An average insert size of both libraries is ~100 kb whereas the genome coverage is estimated as 6 and 12, respectively. BAC analysis and cytogenetic experiments resulted in integration of all linkage groups with the corresponding chromosomes, as well as in identification of several gene-rich regions [22–27]. BAC-derived gene sequences enabled phylogenetic studies of particular gene families [26]. A specific bioinformatic pipeline has been developed to accelerate the analysis and support annotation of lupin sequence data [28]. Recently, draft assemblies were released, spanning about 45–50 % of the *L. angustifolius* genome [17, 29]. The genome sequence length for cv. Sonet was estimated as 924 Mbp using flow cytometry [14] or 1037–1153 Mbp based on the K-number/peak depth model calculation [17, 29]. The opportunities for gene search and comparative mapping were greatly enhanced by the release of comprehensive transcriptome assembly, derived from sequencing of different narrow-leafed lupin tissue types and anchored in the reference linkage map [17]. The recent developments in next generation sequencing techniques have considerably accelerated the progress in legume genomics in general. Up to now, the high quality genome sequences of nine Fabaceae species have been published: *Arachis duranensis*, *A. ipaensis* [30], *Cajanus cajan* [31], *Cicer arietinum* [32], *Glycine max* [33], *Lotus japonicus* [34], *Medicago truncatula* [35], *Phaseolus vulgaris* [36] and *Vigna radiata* [37]. These species represent main clades of Papilionoideae: dalbergioids (*Arachis*), genistoids (*Lupinus*), millettioids (*Cajanus*, *Glycine*, *Phaseolus*, *Vigna*), robinioids (*Lotus*), and the inverted repeat-lacking clade (*Medicago*). Comparative genomic studies between *L. angustifolius* and *G. max* identified a high level of microsynteny in the gene-rich regions. Not only was the gene nucleotide sequence conserved, but also the order and orientation of particular genes in syntenic blocks [23, 24, 26].

The synteny-based approach was applied to identify a gene underlying locus *Ku*, conferring thermoneutrality of *L. angustifolius*, i.e. removing the need for vernalization to promote flowering. This natural dominant mutation was observed in Western Australia as an early flowering off-type in a field of the late flowering cultivar Borre [38]. *Ku* resulted in advancing flowering by 2–5 weeks and therefore was widely introduced to cultivars in Europe and Australia [39]. It was a key advance in that it provided adaptation to major growing areas with light acidic soils in temperate and warmer climatic zones. One of *FLOWERING LOCUS T* (*FT*)-derived markers, dFTc, was localized directly in the *Ku* locus with no trace of recombination event between marker and the trait [18]. Such an observation strongly justified further investigation of this gene as a candidate gene for *Ku*. Very recently, it was revealed that two homologs of *FTc* are present in *L. angustifolius* genome, named as *LanFTc1* and *LanFTc2*. Gene expression profiling of these genes after prolonged exposure to cold temperatures demonstrated that *LanFTc1* is involved in the vernalization independence, whereas *LanFTc2* is not [13]. As the *L. angustifolius* genome appears to have undergone whole genome duplication (WGD) events [18, 40], the question arises whether and to what extent WGD shaped the evolution of *FT* and other PEBP-family genes in this species.

Here, recently developed legume genomic resources were enlisted to survey the *L. angustifolius* genome regions carrying both *LanFTc* genes. *LanFTc* BAC clones were physically localized in *L. angustifolius* chromosomes using fluorescence *in situ* hybridization. Sequenced BACs were mapped to the *L. angustifolius* genome scaffolds to form consensus sequences which were further used for functional annotation and microsynteny search across the legume family. Linkage groups carrying *LanFTc* genes, supplemented with sequence-defined markers, were aligned to the preliminary genome sequence of the species and then exploited for identification of cross genera large, chromosome-scale blocks of collinearity. Nucleotide and protein multiple alignment and protein-based Hidden Markov Model gene prediction were subsequently applied to retrieve PEBP-family sequences from sequenced legume genomes. Bayesian inference of phylogeny was performed to assign PEBP-family sequences to appropriate subclades and to track evolution of particular homologs.

## Results

### Chromosomal localization of *LanFTc* genes

**Fig. 1:**
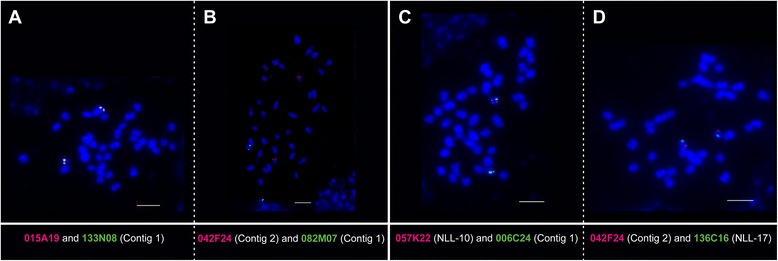
Localization of BAC clones carrying *LanFTc1* and *LanFTc2* genes in *L. angustifolius* mitotic chromosomes. Chromosomes were counterstained with DAPI whereas BAC clones DNA was labelled with tetramethylrhodamine-5-dUTP - red signals and digoxigenin-11-dUPT - green signals. Common localization resulted in yellow signals. Scale bar = 5 μm. The following combinations of clone pairs were used in BAC-FISH: **a**, both clones from contig 1; **b**, one clone from every contig; **c**, a clone from contig 1 and a BAC cytogenetic marker for linkage group NLL-10; **d**, a clone from contig 2 and a BAC cytogenetic marker for linkage group NLL-17

In the narrow-leafed lupin genome two copies of *FTc* homolog exist, *LanFTc1* assigned to linkage group NLL-10 and *LanFTc2* localized in NLL-17 [13]. Based on the sequence annotation, BAC clones carrying these genes were grouped into two contigs, 12 in contig 1 carrying *LanFTc1* and 5 in contig 2 carrying *LanFTc2*. To visualize their chromosomal localization, all clones derived from these contigs were used as molecular probes and subjected to fluorescent *in situ* hybridization (BAC-FISH) to mitotic chromosomes of *L. angustifolius*. Five BACs from contig 1 (006C24, 015A19, 075P11, 082M07, 133N08) and one from contig 2 (042F24) gave single locus signals, whereas the remaining ones produced repetitive signals dispersed over numerous chromosomes. BAC clones hybridizing to single loci were used in various combinations in two-colored BAC-FISH experiments. To verify BAC localization, available chromosome-specific landmarks for linkage groups NLL-10 (clones 057K22 and 077C13) and NLL-17 (003B18 and 136C16) [23–25] were included in the survey. In total, 15 BAC pairs were tested: 5 for contig 1 itself, 4 for contig 1 vs 2, and 6 for contig vs linkage group (Table 1). BAC clones originating from the same contig or linkage group produced overlapping signals on one pair of homologous chromosomes whereas those from different contigs or linkage groups yielded single locus signals on two different chromosome pairs (Fig. 1). BAC clones which hybridized to single loci on mitotic chromosomes were considered as new cytogenetic markers. Together with those already published [23–27], they constituted physical anchors for the integration of genetic and cytogenetic maps of the *L. angustifolius* genome.

**Table 1:** Co-localization of cytogenetic markers for both contigs and *L. angustifolius* linkage groups NLL-10 and NLL-17

	006C24, contig 1	015A19, contig 1	075P11, contig 1	082M07, contig 1	057K22, NLL-10	042F24, contig 2
015A19, contig 1	Y	-	-	N	Y	N
082M07, contig 1	Y	N	Y	-	-	N
133N08, contig 1	-	Y	Y	-	Y	N
042F24, contig 2	N	N	-	N	-	-
003B18, NLL-17	-	-	-	-	-	Y
136C16, NLL-17	-	-	-	-	-	Y
077C13, NLL-10	-	-	Y	Y	-	-

Y: BAC-FISH signals for clones from this pair were observed in the same chromosome pair

N: BAC-FISH signals for clones from this pair were observed in different chromosome pairs

### Anchoring *L. angustifolius* genome scaffolds to the updated reference linkage map

The reference map of the narrow-leafed lupin genome [17] was supplemented with recently published BAC-end sequence (BES)-derived markers from contig 1 (133N08_5, 080K01_3D, 008L15_5, 082M07_3, 130O02_5) and 2 (137O24_5, 071N21_3, 042F24_5, 092M12_5, 042F24_3) [13]. Moreover, the existing BES markers from linkage groups NLL-10 (077C13_3, 057K22_3F2) and NLL-17 (107M16_3, 111G03_5, 024F12_5D, 111L22_5, 141C03_5D, 003B18_3) [23–25] not previously incorporated to the reference map [17] were mapped here. *LanFTc1* and *LanFTc2* regions differed in their estimated recombination frequencies. Between all BAC and gene-based markers from contig 1 no recombination in RILs was observed, whereas for such markers from contig 2 two recombination events were observed (see Additional file 1). 44 sequence-defined markers from linkage groups NLL-10 and 59 from NLL-17 were subjected to repeat masking and *L. angustifolius* genome mapping. Markers from *LanFTc1* and *LanFTc2* loci were not included in this alignment as there were BAC clone sequences available. High confidence alignments to genome sequences were constructed for 29 markers from the NLL-10 and 37 from NLL-17. As some redundant or closely linked molecular markers tagged the same scaffolds, the numbers of unique scaffolds anchored to particular linkage groups were lower: 28 for NLL-10 and 31 for NLL-17 (see Additional file 2). The total length of sequence assigned to the linkage map was calculated as 757 164 nucleotides for NLL-10 and 1 226 451 for NLL-17.

### Assembly and functional annotation of superscaffolds carrying *LanFTc* genes

BES markers from both BAC contigs were aligned to their respective BAC sequences as well as to the narrow-leafed lupin genome assembly. 23 BES markers from contig 1 were anchored in clone 133N08 whereas one BES marker mapped to scaffold28512 (accession KB416309.1). The 5′ part of 133N08 was found to overlap with the scaffold14655 (KB408211.1). 8 BESs from contig 2 were mapped to clone 137O24 but two remained unlinked. 5′ part of 137O24 overlapped with the scaffold72960 (AOCW01133146.1). Based on these results, superscaffolds were constructed, one for *LanFTc1* encompassing 208 734 nt, and the other for *LanFTc2* carrying 189 931 nt.

RepeatMasker and Censor annotation revealed relatively high levels of repetitive content, estimated to occupy as much as 30.2 % of total sequence length in superscaffold-1 and 35.4 % in superscaffold-2. The major components of repeat fraction were retrotransposons, LTR/Gypsy and LTR/Copia. The transposon fraction was represented mainly by DNA/EnSpm. Total percentage sequence occupancy by the transposon fraction was 44-fold (superscaffold-1) and 6-fold (superscaffold-2) lower than that of retrotransposons. As BAC clones were positioned in the superscaffolds by their BAC ends, it was possible to survey the distribution of repeats over particular clones and to compare it with type of BAC-FISH signals yielded by these clones on mitotic chromosomes (Table 2). No direct correlation between type of BAC-FISH signal and total repeat content nor with the presence or prevalence of different repeat families, like LTR/Copia or LTR/Gypsy, was identified. However, it should be noted that repeats were relatively abundant in these clones (from 25.21 % to 49.81 %).

**Table 2:** Repetitive content (% occupancy) of BAC clones and type of BAC-FISH signals observed on mitotic chromosomes

	Contig1 (*LanFTc1*)												Contig2 (*LanFTc2*)				
BAC-FISH^a^	R	R	R	R	R	R	R	S	S	S	S	S	R	R	R	R	S
BAC clone	080 K01	008 L15	124 E17	139 P24	140 E06	130 O02	055 I07	133 N08	015 A19	082 M07	006 C24	075 P11	137 O24	071 N21	100 H14	092 M12	042 F24
DNA other	-	0.33	-	0.28	0.39	0.44	0.39	0.25	0.34	0.43	0.41	0.47	-	-	-	-	-
DNA/EnSpm	-	-	-	-	-	-	-	0.40	-	-	-	-	3.35	-	-	-	-
DNA/hAT	-	-	-	-	-	-	-	0.12	-	-	-	-	1.54	0.86	-	-	-
DNA/IS	-	-	-	-	-	-	-	-	-	-	-	-	0.45	-	-	-	-
LINE/L1	0.59	1.11	0.33	0.93	1.10	1.25	1.11	0.98	1.13	1.21	1.15	1.31	-	-	-	-	-
LINE/RTE	2.99	1.86	3.61	1.56	2.17	1.74	1.55	1.39	1.88	2.38	0.42	0.48	0.31	0.75	0.84	1.24	1.30
LTR/Copia	14.93	17.94	9.77	11.96	16.61	18.42	16.81	13.85	14.43	17.81	17.40	16.34	22.81	25.10	29.69	35.88	18.43
LTR/Gypsy	17.10	14.20	20.66	16.02	9.16	10.41	9.27	12.89	11.67	10.07	9.59	4.97	7.59	15.61	17.53	11.10	27.26
Simple repeat	0.82	1.26	0.99	1.26	1.50	1.52	1.52	1.34	1.47	1.47	1.57	1.63	1.79	1.98	1.71	1.59	2.26
Total repeats	36.43	36.71	35.36	32.01	30.92	33.78	30.66	31.22	30.92	33.36	30.54	25.21	37.83	44.31	49.77	49.81	49.25

^a^R - repetitive BAC-FISH signals dispersed over numerous chromosomes. S – single-locus BAC-FISH signals

**Fig. 2:**
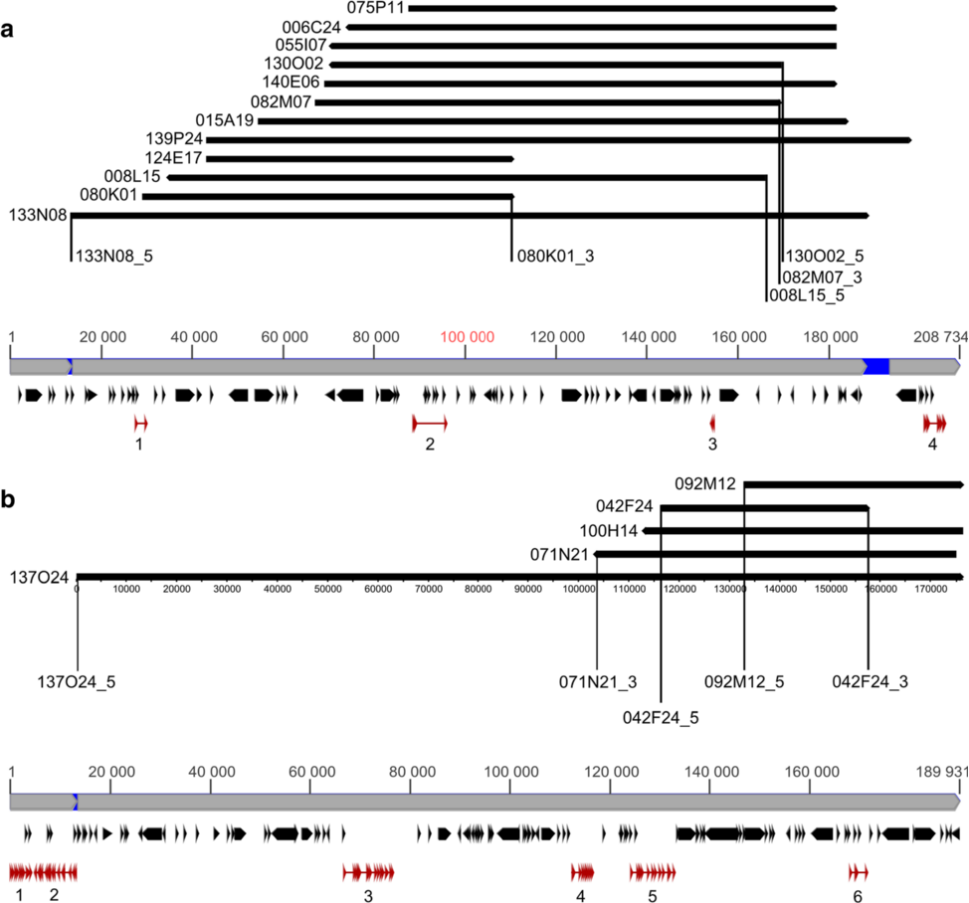
The structure and functional annotation of superscaffolds carrying *LanFTc1* (**a**) and *LanFTc2* (**b**) genes. Consensus superscaffold sequence is visualized by grey bar, whereas overlapping BAC clones by black bars. Vertical lines tag position of BES markers. Black objects represent repetitive elements whereas red ones mark non-repetitive coding sequences. Arrows indicate orientation in consensus sequence

Hidden Markov Model gene prediction based on reference protein sequences identified four and six genes in the superscaffolds, respectively (see Additional file 3). These genes included, besides previously annotated *LanFTc1* and *LanFTc2*, apyrase, acetyl-CoA carboxylase biotin carboxylase subunit, ethanolamine-phosphate cytidylyltransferase, galacturonokinase, and four not functionally characterized sequences (Table 3). Alignment of predicted coding sequences (CDSs) to the transcriptome assemblies of *L. angustifolius* [17], *L. albus* [41] and *L. luteus* [42] provided evidence of expression for 9, 9 and 6 genes, respectively (see Additional file 4). Based on this annotation data, gene density for *LanFTc1* region was calculated as 1.9 genes/100 kbp, whereas for *LanFTc2* as 3.2 genes/100 kbp. Graphical visualization of BAC, BES, linkage marker, repeat and predicted gene model localization in the superscaffolds sequences is presented at Fig. 2.

**Table 3:** Genes identified in superscaffolds and their transcriptomic evidence

Superscaffold	Gene No.	Gene (protein) name	CDS length	Reference accession	Fgenesh + score	Positives (%)	Transcriptomic evidence
1	1	DNA-binding domain protein	684	XP_003624562.1	975.49	77.98	ang, alb
1	2	*LanFTc1*	525	*LanFTc1*	966.60	100.00	ang
1	3	uncharacterized protein	492	XP_006603704.1	498.86	83.07	ang, alb, lut
1	4	apyrase 2-like	1407	XP_006598985.1	1916.76	95.51	ang, alb, lut
2	1	U-box domain-containing protein	2208	XP_006580498.1	2276.42	87.66	alb
2	2	acetyl-CoA carboxylase biotin carboxylase subunit	1614	XP_003630608.2	2159.24	91.81	ang, alb, lut
2	3	uncharacterized protein	2934	XP_006580493.1	2851.19	79.84	ang, alb
2	4	ethanolamine-phosphate cytidylyltransferase	1191	XP_006580492.1	1552.44	88.81	ang, alb, lut
2	5	galacturonokinase-like	1317	XP_006580491.1	1561.85	88.13	ang, alb, lut
2	6	*LanFTc2*	528	*LanFTc2*	978.98	100.00	ang, alb, lut

*Abbreviations*: ang, *L. angustifolius*; alb, *L. albus*; lut, *L. luteus*

### Insight into micro- and macrosynteny of *LanFTc* regions

**Fig. 3:**
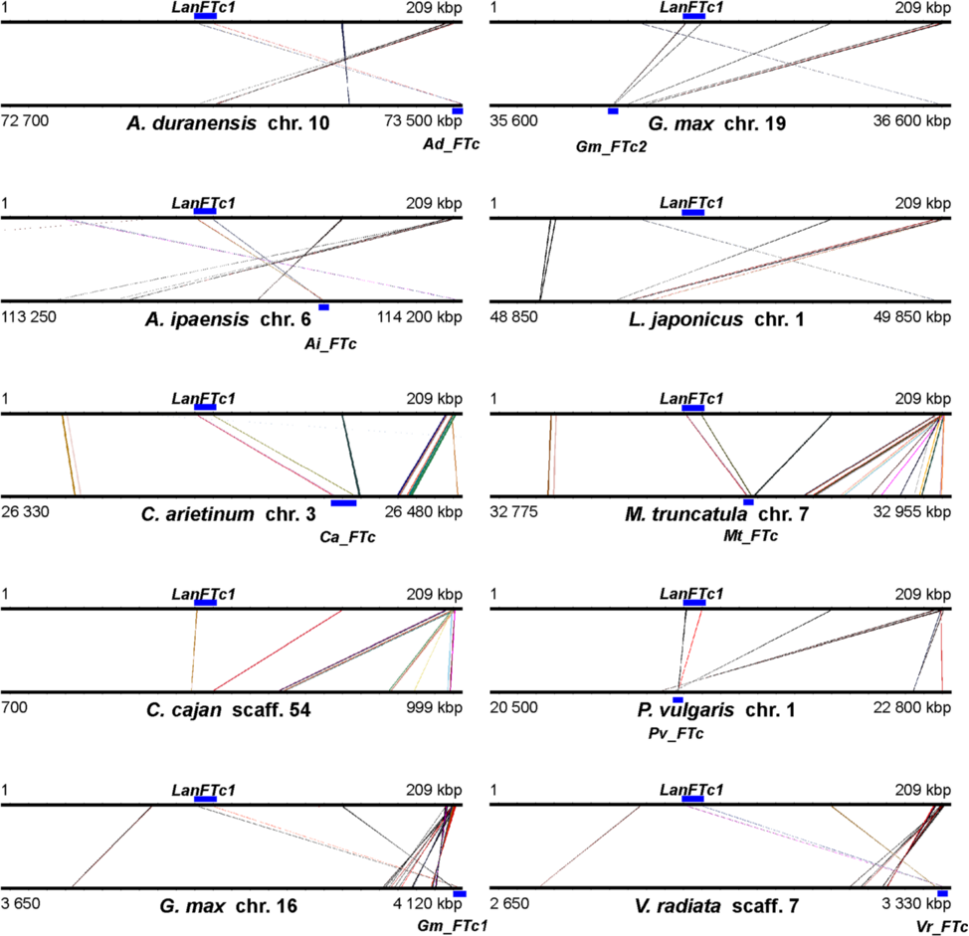
Synteny between superscaffold-1 and corresponding genome regions of nine legume species. Microsyntenic blocks are drawn as Genome Synteny Viewer diagrams. Each graph is composed of two horizontal lines; the upper presents the sequence of *L. angustifolius* superscaffold-1, while the lower line shows the corresponding region of a model legume genome. The scale of the bottom bars is variable, so the chromosome localizations are given. Blue blocks visualize the positions of *FTc* homologs

**Fig. 4:**
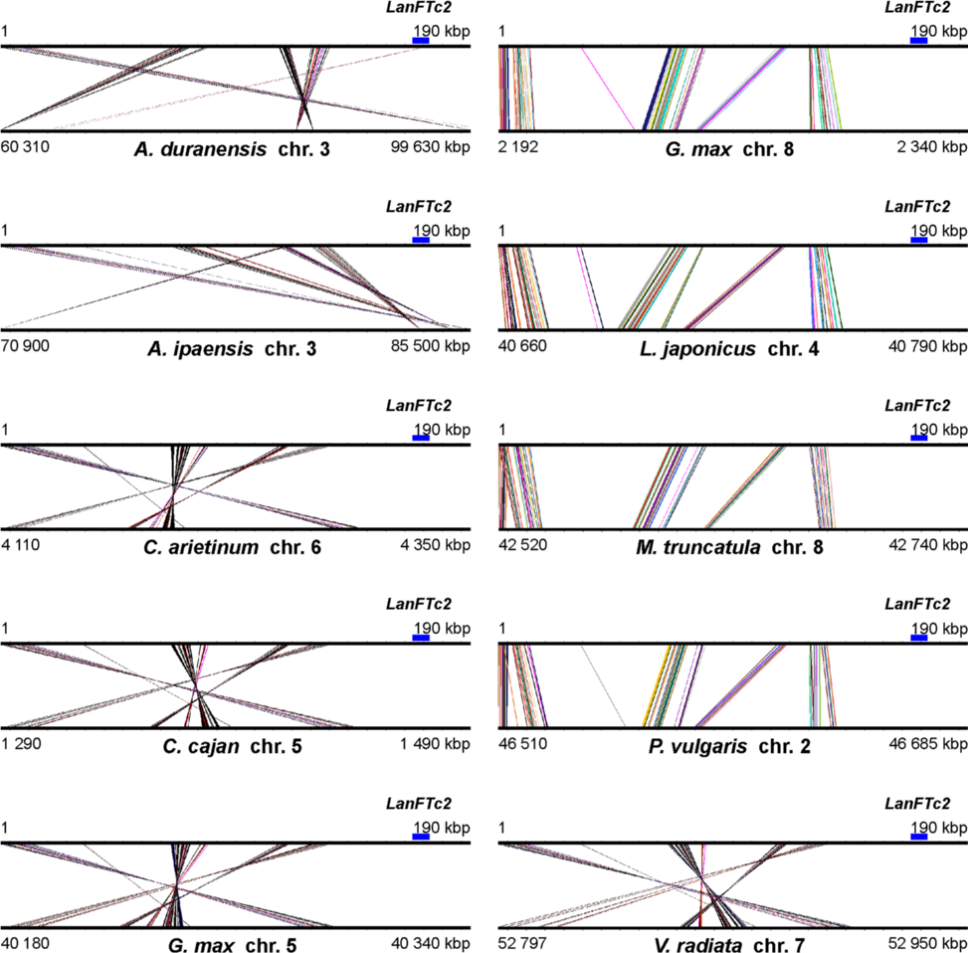
Synteny between superscaffold-2 and corresponding genome regions of nine legume species. Microsyntenic blocks are drawn as Genome Synteny Viewer diagrams. Each graph is composed of two horizontal lines; the upper presents the sequence of *L. angustifolius* superscaffold-2, while the lower line shows the corresponding region of a model legume genome. The scale of the bottom bars is variable, so the chromosome localizations are given. Blue blocks visualize the positions of *FTc* homologs

Both superscaffolds with low complexity regions and repeats masked by RepeatMasker and Censor were aligned to genome sequences of nine legume species: *A. duranensis*, *A. ipaensis* [30], *C. cajan* [31], *C. arietinum* [32], *G. max* [33], *L. japonicus* [34], *M. truncatula* [35], *P. vulgaris* [36] and *V. radiata* [37]. Both regions revealed distinct microsyntenic patterns (see Additional files 5 and 6 where coordinates and statistics of alignments were provided). Superscaffold-1 showed links of sequence collinearity to *A. duranensis* chromosome 10 (region between 72.7 and 73.5 Mbp), *A. ipaensis* chr. 6 (113.2–114.2 Mbp), *C. arietinum* chr. 3 (26.3–26.5 Mbp), *C. cajan* scaffold 54 (0.7–1.0 Mbp), *G. max* chr. 16 (3.6–4.1 Mbp) and 19 (35.6–36.6 Mbp), *L. japonicus* chr. 1 (48.8–49.9 Mbp), *M. truncatula* chr. 7 (32.8–33.0 Mbp), *P. vulgaris* chr. 1 (20.5–22.8 Mbp), and *V. radiata* scaffold 7 (2.7–3.3 Mbp) (Fig. 3). In all these regions but *C. cajan* and *L. japonicus* one copy of *FTc* gene was identified. In the *C. cajan* region showing synteny to *LanFTc1* no *FT* gene was recognized whereas in the *L. japonicus* syntenic region one copy of *FTa* gene was annotated. Superscaffold-2 was found to have higher level of synteny to genome regions of all studied species than superscaffold-1, however, the patterns of preserved sequence collinearity did not include the 3′ end of the superscaffold-2, containing the *LanFTc2* gene (Fig. 4). Syntenic links observed for this scaffold matched different chromosomes than those found for superscaffold-1. Namely, they were as follows: *A. duranensis* chr. 3 (60.3–99.6 Mbp), *A. ipaensis* chr. 3 (70.9–85.5 Mbp), *C. arietinum* chr. 6 (4.1–4.4 Mbp), *C. cajan* chr. 5 (1.3–1.5 Mbp), *G. max* chr. 5 (40.2–40.3 Mbp) and chr. 8 (2.2–2.3 Mbp), *L. japonicus* chr. 4 (40.7–40.8 Mbp), *P. vulgaris* chr. 2 (46.5–46.7 Mbp), *V. radiata* chr. 7 (52.8–53.0 Mbp). No copy of *FT* gene was annotated in any of these syntenic regions.

Comparative mapping of sequence defined markers from linkage groups NLL-10 and NLL-17 resulted in the identification of complex networks of syntenic links to several chromosomes in every species studied (see Additional file 7). Regions syntenic to NLL-10 included those showing microsynteny to superscaffold-1. *LanFTc1* gene appeared to be located in the middle of a large conserved region, marked by 9 linkage map loci spanning a distance of 15.3 cM. Despite some inversions and/or insertions/deletions, the arrays of macrosyntenic links observed in this section between sequences from *L. angustifolius* and studied species were visually similar to each other.

**Fig. 5:**
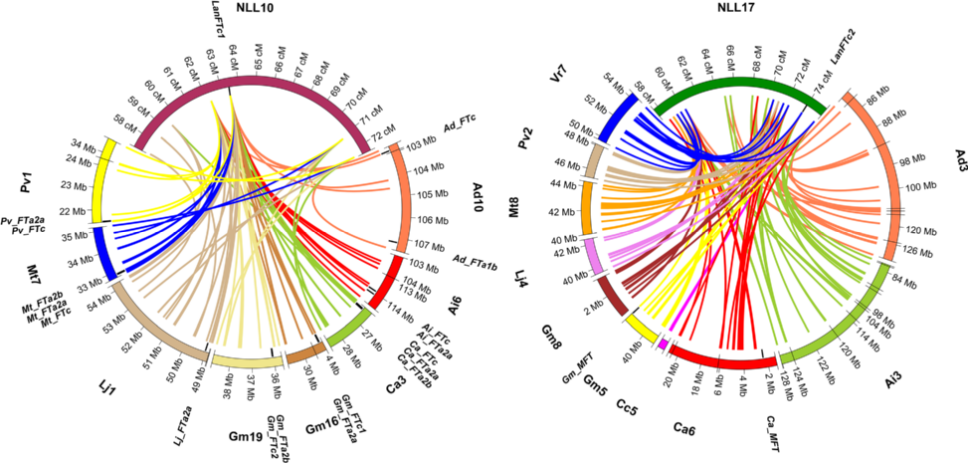
Macrosyntenic links between NLL-10 and NLL-17 linkage groups and selected chromosome sequences. Circos plots show the selected regions of *L. angustifolius* linkage groups and corresponding chromosomes. Ribbons symbolize homologous links identified by DNA sequence similarity

The most conserved arrays of collinearity between NLL-17 and sequenced genome sequences were observed for *A. duranensis* chr. 1, 3 and 5, *A. ipaensis* chr. 1, 3 and 5, *C. arietinum* chr. 6, 7 and 8, *C. cajan* chr. 6, *G. max* chr. 1, 5, 8 and 11, *L. japonicus* chr. 2 and 4, *M. truncatula* chr. 5 and 8, *P. vulgaris* chr. 2 and 3, and *V. radiata* chr. 7 and 11. Those arrays included also all regions (but *C. cajan* chr. 5) matching superscaffold-2 *LanFTc2* (Fig. 5). The syntenic block adjacent to *LanFTc2* gene was flanked by markers 111G03_5 (53.1 cM) and 042F24_5 (73.5 cM). It should be noted that the superscaffold-2 was located at the edge of the large syntenic region (73.0–74.1 cM), with a *LanFTc2* gene itself excluded from this pattern of collinearity.

### Phylogenetic inference of PEBP-family sequences in sequenced legume species

The reference set of *A. thaliana* PEBP-family protein sequences included *FT* (AT1G65480); *TSF* (NM_118156.1), *BFT* (AT5G62040), *MFT* (AT1G18100), and *TFL1* (AT5G03840). The alignment of these sequences to the genome assemblies of 10 legume species resulted in identification of numerous homologs in the analyzed species: *A. duranensis*, 16; *A. ipaensis*, 16; *C. cajan*, 14; *C. arietinum*, 13; *G. max*, 22; *L. japonicus* 6; *L. angustifolius*, 15; *M. truncatula*, 13; *P. vulgaris*, 11; and *V. radiata*, 13. Protein-based gene prediction and CDS mapping revealed that 21 sequences were putatively non-functional, truncated remnants of ancient genes and as such were excluded from further analysis. In total, 118 sequences were classified as full length homologs of legume PEBP-family genes (see Additional file 8). The Bayesian phylogenetic inference of these sequences and five *A. thaliana* references facilitated their assignment to particular subclades and evaluation of gene copy number variation. The highest number of homologs was found in *G. max* (18), whereas the lowest in *L. japonicus* (6) (Table 4). The gene copy number in the *L. angustifolius* (12) was close to calculated mean value for the whole group of species (11.8). The most abundant genes across analyzed taxa were from *FTa*, *FTb* and *TFL1* subclades, whereas the rarest were those from *BFT* and *FTc*. For some subclades, the copy number varied greatly from one species to another. It should be noted that no single copy of *FTc* was found in *C. cajan* and *L. japonicus*, *FTb* in *L. angustifolius* or *BFT* in *L. japonicus*.

**Table 4:** The number of PEBP-family genes identified in ten legume genome assemblies

Species	FTa	FTb	FTc	MFT	TFL1	BFT	Total
*A. duranensis*	3	4	1	2	2	1	13
*A. ipaensis*	4	4	1	2	3	1	15
*C. cajan*	2	2	0	1	3	1	9
*C. arietinum*	3	1	1	1	5	1	12
*G. max*	4	4	2	1	5	2	18
*L. japonicus*	1	2	0	1	2	0	6
*L. angustifolius*	2	0	2	3	4	1	12
*M. truncatula*	3	2	1	1	3	1	11
*P. vulgaris*	2	2	1	1	3	1	10
*V. radiata*	2	4	1	1	3	1	12
Total	26	25	10	14	33	10	118

Some PEBP-family genes were found to be clustered: 17 regions carrying at least two PEBP genes within a distance below 1 Mbp were identified in the genomes of analyzed legume species (Table 5). One of such blocks was identified in *A. duranensis* and *C. cajan*, two in *A. ipaensis*, *C. arietinum*, *M. truncatula*, *P. vulgaris* and *V. radiata*, and five in *G. max*. The most frequent combinations of physically linked FT-family genes were *FTb* itself or one to two *FTa* accompanied by one *FTc*. Despite the presence in numerous copies (from 2 to 5 in analyzed species), only two *TFL1* homologs closely located to each other were identified. No evidence of close localization of *BFT* or *MFT* homologs was found.

**Table 5:** Clusters of PEBP-family genes identified in analyzed legume species

Legume chromosome	CDS positions (kb)	Spanning distance (kb)	PEBP homologs
Ad04	123229.4–123341.6	112.2	Ad_FTb1a, Ad_FTb1b, Ad_FTb1c
Ai04	133275.2–133310.8	35.7	Ai_FTb1a, Ai_FTb1b, Ai_FTb1c
Ai06	113909.7–114074.8	165.2	Ai_FTa2a, Ai_FTc
Ca01	14759.5–14777.3	17.8	Ca_TFL1a1, Ca_TFL1a2
Ca03	26393.9–26444.9	51.0	Ca_FTa2a, Ca_FTa2b, Ca_FTc
Cc07	19108.7–19335.8	227.1	Cc_FTb1a, Cc_FTb2a
Gm08	46616–47459.8	843.8	Gm_FTb1c, Gm_FTb2a
Gm16	4135.9–4164.8	28.9	Gm_FTc1, Gm_FTa2a
Gm16	31110–31151.8	41.8	Gm_FTa1a, Gm_FTa1b
Gm18	57653.7–57673	19.3	Gm_FTb1a, Gm_FTb1b
Gm19	36030.6–36051.9	21.2	Gm_FTa2b, Gm_FTc2
Mt07	774.5–817.9	43.4	Mt_FTb2a, Mt_FTb2b
Mt07	32843.6–32877.1	33.5	Mt_FTa2a, Mt_FTa2b, Mt_FTc
Pv01	21438.4–21457.8	19.5	Pv_FTa2a, Pv_FTc
Pv08	422.5–439.3	16.7	Pv_FTb1a, Pv_FTb2a
Vr_scaffold_7	3318.3–3334.5	16.1	Vr_FTa2a, Vr_FTc
Vr04	20473.1–20507.3	34.2	Vr_FTb1a, Vr_FTb1b, Vr_FTb1c, Vr_FTb2a

**Fig. 6:**
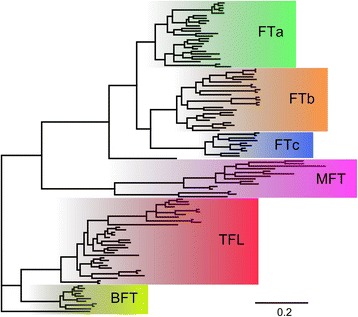
Schematic tree showing majority rule consensus of 2251 trees found in Bayesian analysis of 118 Papilionoid and 4 *A. thaliana* reference PEBP-family coding sequences. 753 nucleotide positions were included in the MAFFT alignment used for phylogenetic inference

**Fig. 7:**
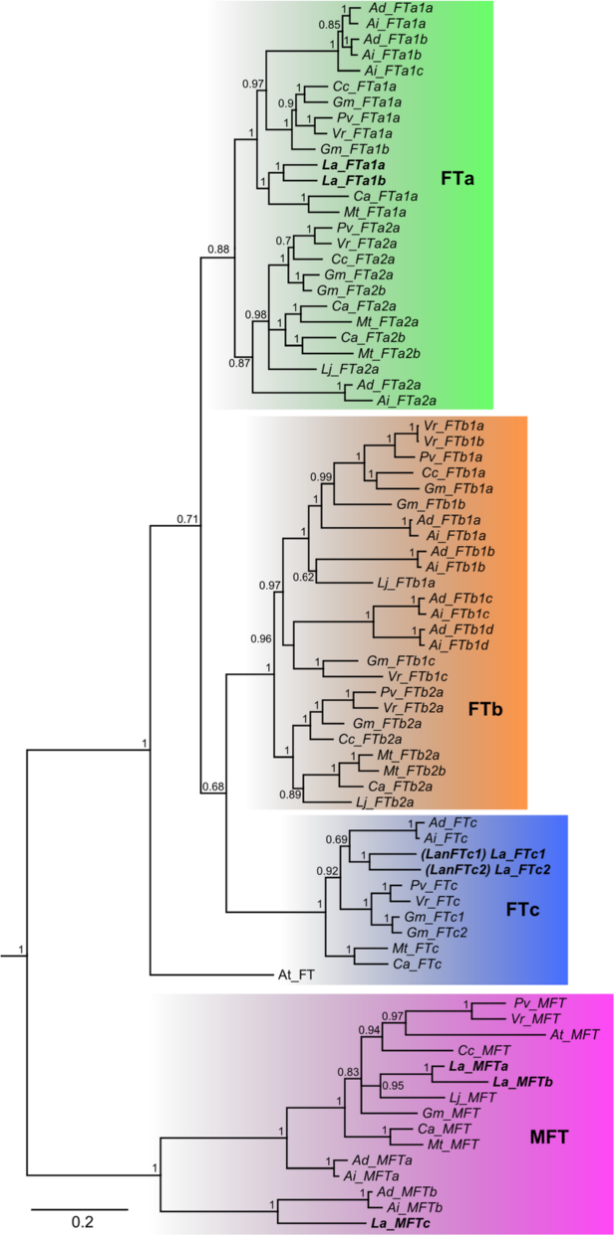
Majority rule consensus of 2251 trees found in Bayesian analysis of 118 Papilionoid and 4 *A. thaliana* reference ﻿PEBP-family coding sequences - *FT* and *MFT* subfamilies. Numbers are posterior probabilities. 753 nucleotide positions were included in the MAFFT alignment used for phylogenetic inference

**Fig. 8:**
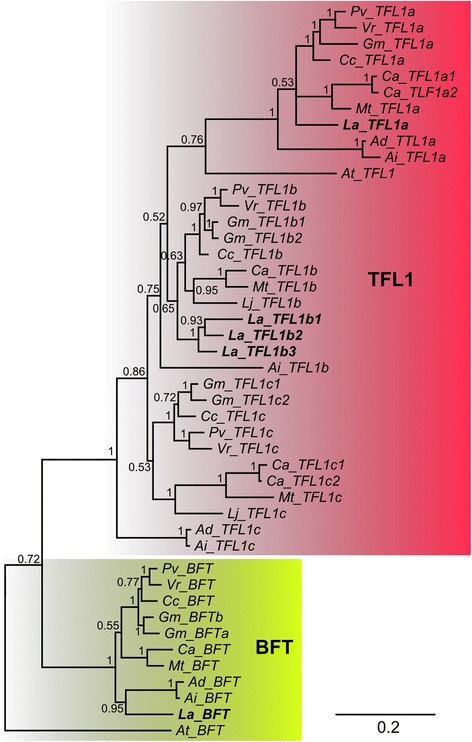
Majority rule consensus of 2251 trees found in Bayesian analysis of 118 Papilionoid and 4 *A. thaliana* reference ﻿﻿PEBP-family coding sequences - *TFL1* and *BFT* subfamilies. Numbers are posterior probabilities. 753 nucleotide positions were included in the MAFFT alignment used for phylogenetic inference

The majority rule consensus tree based on the alignment of 122 PEBP-family sequences (Figs. 6, 7 and 8) shed light on evidences of numerous duplications, visualized by sister branches of shared nodes. In lineages leading to the following species or clades such duplications were observed: *C. arietinum*: two duplications of *TFL1* genes, *G. max*: two *TFL1*, one *BFT*, *FTa* and *FTc*; *V. radiata*: one *FTb*; *M. truncatula*: one *FTb*; *L. angustifolius*: one *FTa*, *FTc* and *MFT*, and 2 *TFL1*, giving three closely related final copies. Duplications occurred also in genomes located at ancestral nodes linking closely related species, like the ancestor of *A. duranensis* and *A. ipaensis*: *FTa*, *FTb*, and *MFT*, and ancestor of *C. arietinum* and *M. truncatula*: *FTa*. The topology of the tree confirmed close relationships between sister *Arachis* species, between *Lotus*, *Medicago* and *Cicer*, as well as between *Glycine*, *Phaseolus*, *Cajanus* and *Vigna*. It also reflected directly more distant relations of early diverged *Arachis* and *Lupinus* to other succeeding clades. To facilitate further sequence analysis, gene names were assigned following the topology of the tree. As *M. truncatula* and *G. max* PEBP homologs had already common names in use [8] reference to those was provided (see Additional file 8).

### Promoter analysis of *FT* homologs

The presence of multiple copies of *FT* in regions with very well preserved sequence collinearity raised a question about the sequence conservation in regulatory regions of these homologs. *FT* upstream sequences of 8000 nt were extracted from all homologs except for *L. angustifolius FTa1a* and *FTa1b* (which are localized in short scaffolds). Annotation revealed no significant similarity to CORE1, CORE2 and S4 elements. Despite numerous alignments, no evidence for conserved collinearity of P2-S3-P1 blocks was identified. Conserved positions and orientations were identified for RE-alpha and CCAAT-box sequences.

### Assay of substitution rates and selective pressure of PEBP-family genes in legumes

According to the results of phylogenetic inference, PEBP sequences were grouped to six subclades, namely *BFT*, *FTa*, *FTb*, *FTc*, *MFT*, and *TFL1*. Based on the tree topology, 61 species-specific pairs of duplicated sequences were selected, including those located at sister and quasi-sister branches (i.e. paralogs) and those of the most recent common ancestor origin (i.e. orthologs). The nonsynonymous to synonymous substitution rate (Ka/Ks) ratio analysis revealed that all pairs but *C. arietinum TFL1d*/*TFL1e* were under strong purifying selection, with Ka/Ks values from 0 to 0.53 (see Additional file 9). The outlier obtained for *C. arietinum TFL1d*/*TFL1e* pair may result from the incorrect assembly of *C. arietinum TFL1e* gene as this sequence was derived from three short contigs (Cascaffold1275, Cascaffold127513210 and Cascaffold127511243) and such a construct may be artificial.

The survey of subgroup-averaged hypothetical divergence time placed *MFT* and *TFL1* as ancestral subfamilies with 157 and 117 million years ago (mya) of non-sister branch gene pair divergence time, respectively (Table 6). The expansion of non-sister branches in *FTa* and *FTb* clades occurred ~65–76 mya. Evolutionary history of legume species-specific paralogous PEBP copies was found to be relatively recent, as particular sister branch genes were dated from averagely 11 mya in *TFL1* to 29 mya in *FTb*.

**Table 6:** Substitutions in PEBP-family paralogous comparisons and estimated divergence time

Subfamily	Species	No. of pairs analyzed	Localization on sister branches	Divergence time (mya)	Ka	Ka/Ks
*BFT*	Gm	1	yes	15.9	0.01	0.08
	Ad	1	genus-specific	9.1	0.05	0.53
	Ad	1	no	94.5	0.21	0.21
	Ai	3	genus-specific	18.1	0.03	0.16
	Ai	1	no	110.8	0.21	0.18
	Ca	2	no	62.4	0.13	0.20
*FTa*	Cc	1	no	79.6	0.13	0.15
	Gm	2	no	47.3	0.08	0.16
	Gm	1	yes	11.0	0.03	0.22
	La	1	yes	32.6	0.06	0.18
	Mt	1	no	46.0	0.12	0.26
	Pv	1	no	90.8	0.13	0.14
	Vr	1	no	63.2	0.13	0.20
	Ad	2	no	69.0	0.14	0.21
	Ai	2	no	67.3	0.14	0.21
	Cc	1	no	56.5	0.16	0.28
	Gm	4	no	54.0	0.19	0.34
*FTb*	Lj	1	no	56.5	0.18	0.31
	Mt	1	yes	12.2	0.04	0.34
	Pv	1	no	73.2	0.19	0.25
	Vr	3	no	66.7	0.22	0.32
	Vr	1	yes	11.2	0.02	0.14
*FTc*	Gm	1	yes	7.2	0.02	0.21
	La	1	yes	41.4	0.11	0.26
	Ad	1	no	139.3	0.37	0.26
*MFT*	Ai	1	no	170.6	0.38	0.21
	La	1	no	162.2	0.37	0.22
	La	1	yes	28.0	0.09	0.32
	Ad	1	no	87.2	0.20	0.22
	Ai	2	no	114.3	0.24	0.22
	Ca	1	no	81.9	0.16	0.19
	Ca	2	yes	4.5	0.01	0.50
	Cc	2	no	119.5	0.14	0.12
	Gm	2	no	131.2	0.17	0.12
*TFL1*	Gm	2	yes	4.9	0.02	0.40
	La	1	no	112.0	0.15	0.13
	La	2	yes	25.0	0.04	0.17
	Lj	1	no	43.0	0.17	0.38
	Mt	2	no	112.4	0.19	0.16
	Pv	2	no	140.6	0.13	0.09
	Vr	2	no	152.8	0.15	0.10

## Discussion

### *FT*-like genes are located in regions of shared cross-genera legume synteny

The analysis based on linkage mapping, micro- and macrosynteny survey, and phylogenetic inference provided clear lines of evidence to support the hypothesis that *LanFTc1* gene, involved in vernalization pathway in *Lupinus angustifolius* L. [13, 18], is a direct descendant of an ancestral *FTc*, whereas *LanFTc2*, not responsive to vernalization, is a more recent duplicate.

The availability of *L. angustifolius* draft genome sequence [29] and reference sequences of nine legume species *A. ipaensis*, *A. duranensis* [30], *C. cajan* [31], *C. arietinum* [32], *G. max* [33], *L. japonicus* [34], *M. truncatula* [35], *P. vulgaris* [36] and *V. radiata* [37] greatly contributed to lupin molecular studies [23, 24, 26, 28]. The cross-clade comparative mapping of genome sequences provided significant improvements to the phylogeny reconstruction of *FT*-like and other genes from the PEBP family in legumes. Based on the model *A. thaliana* and the related crop species *Brassica rapa* it was demonstrated that synteny can be exploited to order gene models by homology providing alignment targets for mapping-by-sequencing [43]. It is a case of many species with missing full reference sequence but partial genome information available. In narrow-leafed lupin, synteny was harnessed to track the evolutionary history of duplicated chalcone isomerase like genes [26] as well as to select candidate gene family (*FT*-like) underlying vernalization insensitivity locus *Ku*, conferring early flowering phenotype [16]. The alignment of the first gene-based linkage map of *L. angustifolius* to the genome sequence of model legume *M. truncatula* revealed the existence of regions of conserved synteny in 14 lupin chromosomes [16]. The section of *L. angustifolius* linkage group NLL-10 containing the *Ku* locus was syntenic with a region of *M. truncatula* chromosome 7 containing three homologs of the *FT* gene. Marker development, followed by linkage mapping of *FT*-like genes identified by *Lupinus* vs *Medicago* synteny, turned out to be a successful approach, and revealed the tight linkage of one of *FTc*-derived markers with locus *Ku* [18]. Moreover, a synteny survey supported the identification of affinities within *FTa*, *FTb* and *FTc* genes in *M. truncatula* and *G. max* genomes [8]. A synteny based approach was also used to track remnants of ancient duplications in *G. max* and resulted in identification which collinear relationships among blocks containing *CO*-like and *FT*-like genes arose after the whole-genome duplication (WGD) events [44]. In the present study, comparative analysis revealed that despite a complex evolution pattern of PEBP family genes, arrays of collinearity in genome regions carrying *FT*-like genes were not substantially disrupted. The survey of sequence collinearity links surrounding PEBP genes considerably supported phylogenetic inference of these genes.

### Copy number of PEBP-family genes in legumes

The research revealed that a majority of PEBP-family genes are present in multiple copies in analyzed legume species. The PEBP family consists of six subclades, namely *FTa*, *FTb*, *FTc*, *MFT*, *TFL1*, and *BFT*. According to our knowledge, presented research is the first whole-genome survey of PEBP gene family across legume species representing several Papilionoideae lineages. It should be emphasized that the estimation of exact number of copies in particular species depends on two main constraints: the quality of the genome assembly and the availability of comprehensive reference transcriptome datasets. In the legume clade, the number of PEBP family genes has already been evaluated for *G. max*, *M. truncatula*, *Pisum sativum* and *P. vulgaris* genomes [8, 45, 46]. PEBP gene copy number was extensively studied in *M. truncatula*, and resulted in identification two *FTa* and *FTb* genes and one *FTc*, *TFL1*, *BFT* and *MFT* gene [11, 47–49]. With the use of the new assembly of the *M. truncatula* genome we were able to identify one more *FTa* sequence and two other copies of *TFL1*. Four *FTa*, four *FTb*, two *FTc*, four *TFL1*, two *BFT* and two *MFT* sequences were identified by BLAST searches of the *G. max* genome [8]. Those results mostly converge with ours but we found one more *TFL1* and one less *MFT* copies. The lacking *MFT* sequence, Gm08g05650, appeared only in the first genome assembly which contained also questionable *FTa* Gm02g07650), two *FTb* (Gm08g28470, Gm18g53670) [46]. Gm02g07650, Gm08g05650, Gm18g53670 and Gm08g28470 are putatively contig-assembly artefacts of the first draft *G. max* genome release because all soybean PEBP gene models but those Gm02g07650, Gm08g05650, Gm18g53670 have conserved structure, and all but Gm08g28470 have duplicated counterparts generated from the last WGD [46]. Moreover, other genome-wide screening resulted in detection of all *G. max* copies reported here except for *TFL1* (Gm13g39360) and *FTb* (Gm08g47820), the loci removed from the new genome assembly [45]. In the present study, novel homologs were extracted from *P. vulgaris* and *M. truncatula* genome sequences, which were not identified in previous research [46], namely *P. vulgaris FTb* Phvul.008G003700.1 and *M. truncatula FTa* (Medtr6g033040.1 and Medtr7g085020.1), *BFT* (Medtr0020s0120.1), and *TFL1* (Medtr1g060190.1). The molecular and *in silico* survey of *P. sativum* PEBP genes resulted in the deciphering of coding sequences of two *FTa*, two *FTb*, one *FTc*, and three *TFL1* homologs [8]. The revealed inconsistency in determination of precise number of PEBP homologs in legumes among recent reports is a minor issue which does not undermine the general conclusion that *FTa*, *FTb* and *TFL1* are present in higher copy number than *FTc*, *MFT* and *BFT*.

### Conserved motifs of FT promoter sequences

Typically, promoters in *A. thaliana* are short with an average length of ~500 bp [50]. However, the *FT* promoter is much longer and contains four major blocks: A, located from ~0.4 kb to the first codon; B, at ~ -2 kb; ID, at ~ -3.7 kb; and C, at ~ -5.5 kb. Block A contains conserved sequences: *CO*-responsive elements (CORE1 and CORE2) directly bound by CO, and S1, P1, S3, P2, S4 with unknown functions [51, 52]. Block B carries E-box binding site for basic-helix-loop-helix proteins promoting *FT* expression under blue light [1, 51]. Block ID has no annotated element, however, it shows length variation in *Arabidopsis* natural populations caused by at least two independent indel events. There were some differences between these types in fitness of plants grown over winter but not in flowering time [53]. Taking into consideration the position of a large indel in the *LanFTc1* promoter (~-2.8 to ~ -4.2 kb) [13], it may correspond to Arabidopsis block ID, however, it shows lack of sequence conservation. Block C contains RE-alpha and CCAAT boxes. CCAAT box is a binding site for the NUCLEAR FACTOR Y (NFY) complex participating in making long distance DNA loop and activation of *FT* by bringing CO to CORE1 and CORE2 [52]. In general, RE-alpha and CCAAT boxes were found to be present in legume *FT* promoters at expected positions (at ~ -5 kb to ~ -7 kb in legumes, ~-5.3 kb in *A. thaliana*) indicating that *FT* promoter lengths in legumes are at least as big as in *A. thaliana*. It may also indicate that the *NFY*-mediated mechanism is preserved in legumes.

The lack of annotation of several important blocks, like CORE1 and CORE2, may relate to evolutionary sequence divergence rather than a true absence. However, it is well known that vernalization pathways evolved in temperate Cenozoic when global cooling occurred and differ between plant families which were already separated at that time [54–56]. As *FT* is a vernalization-responsive gene, different regulatory elements in *FT* promoter sequence might be present in legume clade. It was evidenced for *A. thaliana* that vernalization causes the accumulation of repressive histone modification marks at *FLC* locus, like H3K27me3, silences this gene and, therefore, unlocks *FT* expression [57]. Differences in vernalization requirement between winter and summer *A. thaliana* accessions are conferred by allelic variation at *FRIGIDA* and *FLC* loci, causing loss or considerable reduction of function [58–60]. In *L. angustifolius* different mechanism of vernalization independence exists, based on large deletion in the promoter of *FT*, a target gene for *FLC* [13].

### Duplicated genes as traces of ancient WGD events

In the present research, novel patterns of species-specific PEBP family gene duplications in all but *L. japonicus* genomes were identified, as well as remnants of preceding ancient duplications, which are thought to have occurred at ancestral nodes shared by several genera. Are these multiple copies traces of ancient WGDs or appeared independently to these events?

Episodes of WGDs have been frequent through angiosperm history [61]. It was proposed that a WGD event occurred in the common ancestor of all extant seed plants (dated ~319 million years ago, mya), which was later followed by the another WGD in the common ancestor of all extant angiosperms (~192 mya) [62]. Moreover, a phylogenomic approach harnessed to investigate the timing of gene set duplications located on syntenic gamma blocks resulted in identification of traces of old genome triplication (~117 mya), associated with early diversification of the core eudicots [63]. It is anticipated that ancestral WGDs facilitated the diversification of regulatory genes important to seed and flower development and therefore ultimately contributed to major innovations which promoted the rise and eventual dominance of seed plants [64]. Taking into consideration all newly discovered and those well-documented and widely accepted genome duplications, even the relatively small genome of *Arabidopsis* should carry the traces of at least five WGDs [64]. However, gene pairs formed by duplication usually have a relatively short life span, as some copies may be lost, others pseudogenized, and only a limited number survive after duplication [46]. In the legume clade, an ancient WGD occurred in the progenitor line of papilionoids, the remnants of which can be seen in extant clades including the genistoids (e.g. *L. angustifolius*), dalbergioids (*Arachis* spp.), millettioids (*P. vulgaris*, *G. max*, *C. cajan*, *V. radiata*), galegoids (*M. truncatula*, *L. japonicus*, *C. arietinum*), Xanthocercis and Cladrastis [40, 65–68]. This event was dated to about 44–65 mya and directly predated the divergence of ancient lineages of Papilionoideae [32, 40, 65, 69, 70]. It should be noted that evidence was found for several independent WGDs which occurred near the base of other important legume lineages, i.e. Mimosoideae-Cassiinae-Caesalpinieae, Detarieae, Cercideae and *Lupinus* clades, dated roughly ~30–55 mya [40]. Lupin WGD is believed to have occurred before the divergence of New World and Old World clades [26, 40]. Some legume WGD events occurred in evolutionary recent times, like 13 mya in *G. max* and several mya in *Arachis* [33, 40]. The Ks analysis of 110 duplicated *C. arietinum* genome blocks showing synteny to four legume (*M. truncatula*, *L. japonicus*, *G. max*, and *C. cajan*) and two non-legume dicot (*A. thaliana* and *Vitis vinifera*) genomes indicated a papilionoid divergence time of 58 mya ago [32]. Galegoid and millettioid clades separated ~54 mya [70]. The survey of galegoid genome sequences using genetic distance–transversion rates at fourfold degenerate sites dated the divergence of *C. arietinum* from *L. japonicus* ~20–30 mya and from *M. truncatula* ~10–20 mya [32]. Estimated dates of major WGD events converges not only with the divergence of the particular lineages but also with the appearance of new PEBP gene copies. Analysis of synonymous substitution rates in soybean genome indicated that the most of PEBP duplicated gene pairs originated from two WGD episodes, dated as 59 and 13 mya, and only few sequences appeared relatively recently, about 3–7 mya [45]. The survey of collinear relationships of homologous *G. max* blocks containing *FT*-like genes revealed that *FTa* and *FTc* all experienced WGDs as well as tandem duplications [44]. In general, the *G. max* genome has a very high number of retained duplicate genes, including terminal duplicates (visualized as sister genes in the gene trees), derived from the WGD event. Similar observations were made for *Arachis* and *Lupinus* genomes [40].

### Contribution of duplications to the evolution of PEBP genes

It is anticipated that the basal clade among PEBP genes are *MFT*-like genes, which are present in angiosperms, gymnosperms, lycophytes and bryophytes [71]. The most likely scenario is that the ancestral *FT*/*TFL1* genes originated from a duplication of an *MFT*-like gene after divergence of the basal plant lineage, lycophytes [46] and could have contributed to the radiation of seed plants [72]. The second duplication resulting in the production of *FT* and *TFL1* clades, encoding the proteins conferring antagonistic functions, likely occurred with the evolution of flowering plants and contributed to the development of angiosperm lineage [44, 71]. The *TFL1* ancestor underwent two separate duplication episodes in the common ancestor of angiosperms, which created *BFT* and *TFL1*lineages, followed by another *TFL1* duplication in the lineage leading to the Papilionoideae [46]. The pattern of *FT*-like duplications is more complex: they were at least three subsequent and two parallel duplications of *FT* genes during the evolution of legumes (including ancient Eurosids I specific tandem duplication) [46]. It may be summarized that all these duplications resulted in the origin of subclades *FTa*, *FTb* and *FTc* as well as in the multiplication of gene copy number in *FTa* and *FTb* clades.

In the present study numerous lineage-specific duplications of legume PEBP-family genes were revealed (Figs. 6, 7 and 8). Gene pair divergence timing based on Ka/Ks analysis presented here (Table 6) strongly evidenced that major *MFT* and *TFL1* lineages originated before the well-documented WGD event around the time of the papilionoid diversification (~157 and ~117 mya vs ~48–65 mya). The estimated date of expansion of legume-specific orthologous copies in *FTa* and *FTb* converges with the papilionoid ancestor WGD date (~76 and ~65 mya vs ~48–65 mya). The value of 76 mya obtained for *FTa* resulted putatively from the existence of remnants of ancient Eurosids I specific tandem duplication in this subclade. The present study provided also novel evidence of lineage-specific WGDs, demonstrated by orchestrated convergence of averaged sister-branch gene pair duplication dates for *FTa*, *FTb* and *FTc*, evaluated as 24, 29 and 24 mya, respectively. Taking into consideration the total number of WGD events in the history of legumes and their ancestral lines, evolutionarily ancient *MFT* and *BFT* subclades were subjected to at least three WGDs. Such observations considerably complement the knowledge on the complex evolutionary scheme of PEBP family in legumes and validates the conclusion that the WGD events were major mechanisms launching the divergence of two large PEBP subfamilies, *FT* and *TFL1*, and to a lesser extent, *BFT* and *MFT*. Thus, despite so many WGD episodes, evolutionary old *MFT* and *BFT* subclades did not accumulate as many legume paralogs as *FT* and *TFL1*. Indeed, the vast majority of hypothetical WGD-derived gene copies must have been completely annihilated in these subfamilies.

### Functional divergence of duplicated PEBP genes

Different evolutionary patterns are possible when duplicated gene copies appear in the genome. Homologous copies of a particular gene may acquire different functions, by several possible mechanisms: pseudogenization, subfunctionalization and neo-functionalization, which can lead to the evolutionary divergence [9, 10, 73]. Indeed, functional diversification of the surviving duplicated genes is a major feature of the long-term evolution of polyploids [9]. Genes from the PEBP family, as accumulated in numerous copies in legume genomes, may be preferential targets of natural selection. Insights from patterns of molecular adaptation based on nucleotide variation among large subset of *M. truncatula* accessions from natural populations justified the conclusion that existing polymorphism of *FT* genes in this species has probably been shaped by recent or ongoing positive selection [74]. However, our study provided well-supported evidence of strong purifying selection of PEBP genes in legumes.

Mutations are considered to play key roles in coding sequence evolution. Mutations are powerful mechanisms to facilitate the evolution of new functions as by providing the increased genetic diversity to drive the divergence of the duplicated proteins and creating discrete folding pathways contributing to the conformational diversity of newly-emerged proteins [75]. To exemplify, *A. thaliana TFL1* and *FT* are key controllers of flowering but have opposite functions: *TFL1* is a repressor, *FT* is an activator [76]. Although these sequences are less than 60 % identical, just the substitution of single amino acid (Tyr85/Gln140 in *FT* and His88/Asp144 in *TLF1*) is sufficient to functionally convert *TFL1* to *FT* and *vice versa* [77, 78]. However, analysis of legume and other *FT* genes revealed that although these sequences are highly conserved in rosid *FT* genes, the legume *FT* genes differ from the consensus at 3 to 6 positions, with *FTc* proteins having a His rather than Gln at 140 [11]. The complete set of amino acids that drove functional divergence of all PEBP subfamilies has not yet been identified. Up to now, 46 amino acid residues that exhibited high conservation with 95 % identity in the alignment encompassing *FTa*, *FTb*, *FTc*, *BFT*, *TFL1* and *MFT* subclades were identified [46]. About 20 % of these residues have been shown to cause loss of function when mutated [76, 79–81]. Such observations may suggest that these conserved amino acids are the prerequisites of maintaining basic functions of PEBP proteins.

## Conclusions

WGD events together with tandem duplications were major mechanisms driving the divergence of two large PEBP subfamilies, *FT* and *TFL1*, and to a lesser extent, *BFT* and *MFT*.

Numerous lineage-specific duplications of PEBP-family genes occurred during the evolution of Papilionoideae. WGD resulted in the origin of subclades *FTa*, *FTb* and *FTc* as well as in the multiplication of gene copy number in *FTa* and *FTb* clades. Duplicated legume PEBP genes were subjected to strong purifying selection.

Two *L. angustifolius LanFTc* genes are paralogs. *LanFTc1* gene, involved in vernalization pathway is a direct descendant of ancestral *FTc*, whereas *LanFTc2*, not responsive to vernalization, appeared by subsequent duplication.

*LanFTc1* gene is located in the genome region showing synteny to corresponding *FT*-like gene regions in numerous legume species, representing all main lineages of Papilionoideae. Despite a complex evolution pattern of PEBP family genes, arrays of collinearity in genome regions carrying *FT*-like genes were not substantially affected.

## Methods

### Chromosome localization of *LanFTc* genes (BAC-FISH)

DNA from clones selected from the cv. Sonet BAC library by *LanFTc* probe hybridization was isolated using the QIAprep Spin Miniprep Kit (Qiagen, Velno, Nederlands) and verified by PCR using insert DNA template and *LanFTc1* and *LanFTc2* gene-based primers [13]. Agarose gel (1 %) electrophoresis was performed for 3 h at 9 V/cm to determine the quality of DNA inserts. Size marker GeneRuler™ 1 kb Plus (Fermentas Waltham, MA, USA) was used.

Cytological preparations of mitotic metaphase chromosomes were made from dissected root tip meristematic tissue. Chromosome squashes and the BAC-FISH procedure were performed according to the protocol [25], with few minor modifications. These changes included: probe labelling with digoxygenin-11-dUTP and tetramethyl-rhodamine-5-dUTP by incubation at 15 °C for 110 min followed by inactivation at 65 °C for 15 min using Sensoquest Labcycler (Göttingen, Germany) and hybridization at 37 °C for 22 h.

### Linkage mapping of sequence-defined markers

The mapping population comprised 89 F_8_ recombinant inbred lines (RILs) developed from the cross combination 83A:476 (domestic) × P27255 (wild-type) *L. angustifolius* [15]. The reference genetic map of *L. angustifolius* [17] containing skeleton and redundant markers was imported to MapManager v. QTXb20 [82]. Based on the segregation pattern, new markers were distributed in the existing linkage groups (map function Kosambi, linkage criterion 1e-4). MapChart software [83] was used to draw the *Lupinus* linkage groups. The approximate positions of attached markers were calculated by linear interpolation of adjacent markers.

### Anchoring genome sequence to linkage map

Marker sequences from linkage map groups NLL-10 and NLL-17 were used to screen the collection of *L. angustifolius* whole-genome shotgun contigs and scaffolds [29] obtained from the NCBI sequence database (Project No. PRJNA179231, assembly version GCA_000338175.1, accessions AOCW01000001–AOCW01191454). The BLAST algorithm was optimized for highly similar sequences (word size, 28; match/mismatch scores, 1/-2; and gap costs, linear). Scaffolds producing alignments with sequence identity value above 99 % were integrated to the map at corresponding marker positions. Alignments with sequence identity value of 95–99 % were manually checked for the possibility of sequencing or assembly errors and appropriate scaffolds were placed on map in case of positive verification.

### Assembly of superscaffolds carrying *LanFTc* genes

BAC-end sequences (BESs) from *LanFTc* contigs, 24 for contig 1 and 10 for contig 2 [13], and BES-derived marker sequences were aligned to sequenced *LanFTc* BAC clones, 133N08 and 137O24 (accessions LN851864 and LN851865, respectively) as well as to *L. angustifolius* whole-genome shotgun assembly [29]. The BLAST (https://blast.ncbi.nlm.nih.gov/Blast.cgi) algorithm [84] was optimized for highly similar sequences (word size, 28; match/mismatch scores, 1/-2; and gap costs, linear). Scaffolds producing alignments with sequence identity value above 99 % to particular BACs or BESs were used to assembly longer, consensus sequences of *LanFTc1* and *LanFTc2* regions. Based on the alignments, BESs were positioned in the constructed superscaffolds.

### Functional annotation of superscaffolds

Repetitive elements were annotated and masked using RepeatMasker Web Server version 4.0.3 with implemented repeat libraries RMLib 20140131 and Dfam 1.4 (A.F.A. Smit, R. Hubley & P. Green, unpublished data, http://www.repeatmasker.orgo). The following options were selected: search engine, cross_match; speed/sensitivity, slow; DNA source, *Arabidopsis thaliana*. Preliminary masked DNA sequences were then compared to a database of transposable element encoded proteins. A third round of masking was performed using Censor maintained at Genetic Information Research Institute [85] with the following settings selected: sequence source, Viridiplantae; force translated search; mask pseudogenes. The annotation of the genetic elements was based on comparative analyses with known sequences. Integrated, non-redundant, sequences of genomic DNA, transcripts, and proteins in the RefSeq database (http://www.ncbi.nlm.nih.gov/refseq) were examined by BLAST. Moreover, scaffolds were subjected to sequence homology searches against the transcriptome sequences of *L. luteus* [42], *L. albus* [41], and *L. angustifolius* [17]. The following sequence repositories were used: *L. albus*, http://comparative-legumes.org (gene index LAGI 1.0), *L. luteus*, http://sra.dnanexus.com/studies/SRP014198/runs (project PRJNA170318, archive SRX159101), *L. angustifolius* (NCBI accession: GBRP00000000.1). CoGe BLAST algorithm [86] was used with the following parameters: e-value cut-off, 1e-10; word size, 8; gap existence cost, 5; gap elongation cost, 2; nucleotide match/mismatch scores, 1/-2. Sequences producing alignments with the lowest e-values were selected as references for protein-based Hidden Markov Model gene prediction in Fgenesh + [87]. Annotation data were exported to European Molecular Biology Laboratory (EMBL) feature table format and visualised in Geneious v8.1.5 [88].

### Micro- and macrosynteny survey

Both superscaffold sequences, masked for repetitive contents and low-complexity regions, were aligned to the following genome sequences: *A. duranensis* (accession V14167) and *A. ipaensis* (accession K30076) (Peanut Genome Project), *C. cajan* [31] (project PRJNA72815, v1.0), *C. arietinum* [32] (v1.0 unmasked, http://comparative-legumes.org), *G. max* [33] (Phytozome v9.0 unmasked, http://www.phytozome.net), *L. japonicus* [34] (pseudomolecules v2.5 unmasked, http://www.kazusa.or.jp), *M. truncatula* [35] (strain A17, JCVI v4.0 unmasked, http://www.jcvi.org/medicago/), *P. vulgaris* [36] (v1.0, https://phytozome.jgi.doe.gov/pz/portal.html), and *V. radiata* [37] (project PRJNA243847). The CoGe BLAST algorithm [86] was used to make sequence similarity analyses with the following parameters: e-value cut-off, 1e-10; word size, 8; gap existence cost, 5; gap elongation cost, 2; nucleotide match/mismatch scores, 1/-2. Sequences producing alignments to numerous loci dispersed over many chromosomes were marked as repetitive and excluded from further analysis. Sequence collinearity blocks were visualized using the Web-based Genome Synteny Viewer [89] and Circos [90].

All marker sequences from linkage groups NLL-10 and NLL-17 [17, 23, 24] were repeat-masked by RepeatMasker and Censor [85]. Masked sequences were then aligned to the *L. angustifolius* whole-genome shotgun assembly [29] using BLAST algorithm optimized for highly similar sequences. Alignments with sequence identity value below 95 % were discarded, whereas alignments with sequence identity value equal or above 95 % were manually checked for the distribution of mismatches, and if accepted, appropriate markers were replaced by scaffolds. Obtained set of markers and scaffolds with assigned genetic distance positions was subjected to RepeatMasker and Censor [85] masking, followed by CoGe BLAST [86] mapping to sequences of nine sequenced legumes (as described).

### Identification of PEBP-family homologs in sequenced legume genomes

Selected *A. thaliana* reference sequences (*FT* (AT1G65480); *TWIN SISTER OF FT*, *TSF* (NM_118156.1), *BROTHER OF FT*, *BFT* (AT5G62040), *MOTHER OF FT*, *MFT* (AT1G18100), and *TERMINAL FLOWER 1*, *TFL1* (AT5G03840) were aligned to the *G. max* genome sequence by custom BLAST implemented in Geneious [88] under the following parameters: e-value cut-off, 1e-6; word size, 7; gap existence cost, 5; gap elongation cost, 2; nucleotide match/mismatch scores, 2/-3. Genome sequence regions, extended by 10000 nt in both directions from alignment locus, were extracted and submitted to Fgenesh + [87] gene prediction using original *A. thaliana* protein sequences as references. Coding (CDS) and protein sequences obtained from Fgenesh + were compared with those deposited in Phytozome *G. max* annotated genome assembly at appropriate nucleotide positions (https://phytozome.jgi.doe.gov/pz/portal.html). To facilitate further tracking of these sequences, Phytozome accession numbers were added to sequence names. The set of *G. max* PEBP-family protein sequences was subsequently used to screen the genome assemblies of *L. angustifolius* [29] and eight other legume species using the same settings as those applied for *G. max*. The transcriptome unigene assembly of *L. angustifolius* was also searched [17]. Fgenesh + gene predictions were made using *G. max* protein sequences as references. Predicted PEBP-family protein sequences were compared with annotation data deposited at PeanutBase http://www.peanutbase.org (*A. duranensis* and *A. ipaensis*) and LegumeIP database [91] (*C. cajan*, *C. arietinum*, *L. japonicus*, *M. truncatula*, *P. vulgaris*). Accession numbers from these databases were assigned to predicted sequences if applicable.

### Phylogenetic inference of PEBP-family sequences

The set of 122 PEBP-family protein coding sequences, including 4 reference *A. thaliana* sequences, was selected (see Additional file 10). Multiple translation sequence alignment was achieved in MAFFT v7.017 [92], using the following parameters: standard genetic code, BLOSUM 62 substitution matrix, gap open penalty 1.25. Two algorithms were tested, G-INS optimized for global homology, and E-INS for multiple conserved domains and long gaps. Bayesian inference of phylogeny was performed in MrBayes 3.2.2 [93]. A phylogenetic tree was drawn in Geneious [88] using specified parameters and settings (see Additional file 11). Based on the topology of the tree, paralogous and orthologous pairs of sequences were selected. Pairwise translation sequence alignments were done in MAFFT v7.017, using E-INS, BLOSUM 80 and gap open penalty 1.25. Selection pressure parameters, Ka (the number of nonsynonymous substitutions per nonsynonymous site), Ks (the number of synonymous substitutions per synonymous site), and Ka/Ks ratios were calculated in DnaSP 5 [94]. The estimation of dates of duplication events was based on the Ks parameter [45].

### Identification of conserved motifs in *FT* promoter sequences

Upstream regions counting 8000 nt from the first triplet of *FT* homolog CDS sequence were extracted from the genome assemblies. Selection of reference motifs was based on recently published data [51] (Table 7). Annotation was done in Geneious [88] using 100 % nucleotide identity threshold for short motifs (up to 8 nt in length) and minimum length of 13 nt for longer motifs.

**Table 7:** Conserved motifs used for *FT* promoter screening

Motif	Sequence
CCAAT-box	ATTGGA
CORE2	GATTGTGGTTATGATTT
E-box	ACAAGTGG
I-box	TTATCAA
P1	ACCACA
P2	GTGTGGT
RE-alpha	TTGGTTG
S1	TAGAT
S2_CORE1	CAATGTGTGATGTACGTAG
S3	TTGGAA
S4	ATAATTTGGAATATT
TATA_box	TATAA

## Additional files

Additional file 1: Segregation data for markers in *L. angustifolius* linkage groups NLL-10 and NLL-17. (XLS 132 kb)
Additional file 2: Centimorgan positions and alignment statistics for *L. angustifolius* genome scaffolds anchored to the updated reference linkage map (groups NLL-10 and NLL-17). (XLS 49 kb)
Additional file 3: Reference accessions assigned to genes predicted in superscaffolds carrying *LanFTc1* and *LanFTc2* genes. (XLS 34 kb)
Additional file 4: Transcriptome sequences assigned to genes predicted in superscaffolds carrying *LanFTc1* and *LanFTc2* genes. (XLS 48 kb)
Additional file 5: Microsyntenic links identified between superscaffold-1 and legume genome regions. Sequences producing numerous links dispersed over many chromosomes were denoted as “repetitive”. (XLS 837 kb)
Additional file 6: Microsyntenic links identified between superscaffold-2 and legume genome regions. Sequences producing numerous links dispersed over many chromosomes were denoted as “repetitive”. (XLS 259 kb)
Additional file 7: Sequence homology links identified between linkage groups NLL-10 and NLL-17 and legume genomes. (DOC 3709 kb)
Additional file 8: PEBP-family reference sequences and homologs identified in the genomes of ten legume species. (XLS 74 kb)
Additional file 9: Substitution rates, selective pressure and estimated divergence time of PEBP-family genes in legumes. (XLS 56 kb)
Additional file 10: The set of 123 PEBP-family protein coding sequences used for phylogenetic inference. (DOC 95 kb)
Additional file 11: Settings applied to perform MrBayes inference of phylogeny. (DOC 31 kb)

## Abbreviations

Ad: *Arachis duranensis*
Ai: *Arachis ipaensis*
BAC: Bacterial artificial chromosome
BES: BAC-end sequence
*BFT*: *Brother of FT and TFL1*
Ca: *Cicer arietinum*
Cc: *Cajanus cajan*
CDS: Coding sequence
FISH: Fluorescent *in situ* hybridization
*FLC*: *Flowering locus C*
*FT*: *Flowering locus T*
Gm: *Glycine max*
La: *Lupinus angustifolius*
*LanFTc1*: *Lupinus angustifolius FTc1*
*LanFTc2*: *Lupinus angustifolius FTc2*
Lj: *Lotus japonicus*
*MFT*: *Mother of FT and TFL1*
Mt: *Medicago truncatula*
NLL: Narrow-leafed lupin linkage group
PEBP: Phosphatidylethanolamine binding protein
Pv: *Phaseolus vulgaris*
*TFL1*: *Terminal flower 1*
*TSF*: *Twin sister of FT*
Vr: *Vigna radiata*
