# Supplementary material for: Expansion of the phosphatidylethanolamine binding protein family in legumes: a case study of Lupinus angustifolius L. FLOWERING LOCUS T homologs, LanFTc1 and LanFTc2
Source: BMC Genomics. 2016 Oct 21;17:820. doi: 10.1186/s12864-016-3150-z (PMC5073747; doi:10.1186/s12864-016-3150-z)

**Sequence homology links identified between linkage groups NLL-10 and NLL-17 and sequenced legume genomes**

NLL-10 - *L. angustifolius* linkage group 10;

NLL-17 - *L. angustifolius* linkage group 17;

Ad - *A. duranensis*;

Ai - *A. ipaensis*;

Ca - *C. arietinum;*

Cc - *C. cajan*;

Gm - *G. max*;

Lj - *L. japonicus;*

Mt - *M. truncatula*;

Pv - *P. vulgaris*;

Vr - *V. radiata*;


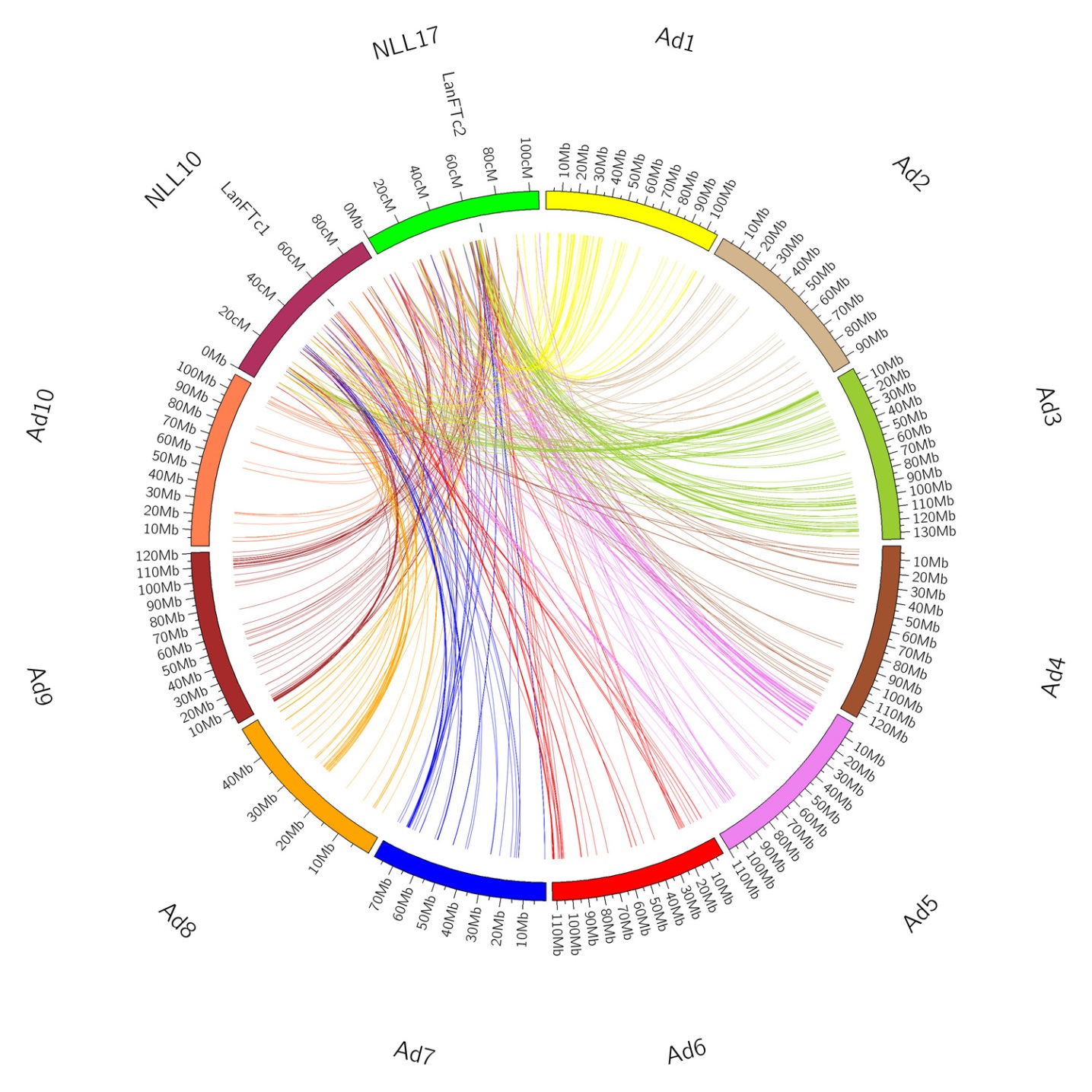


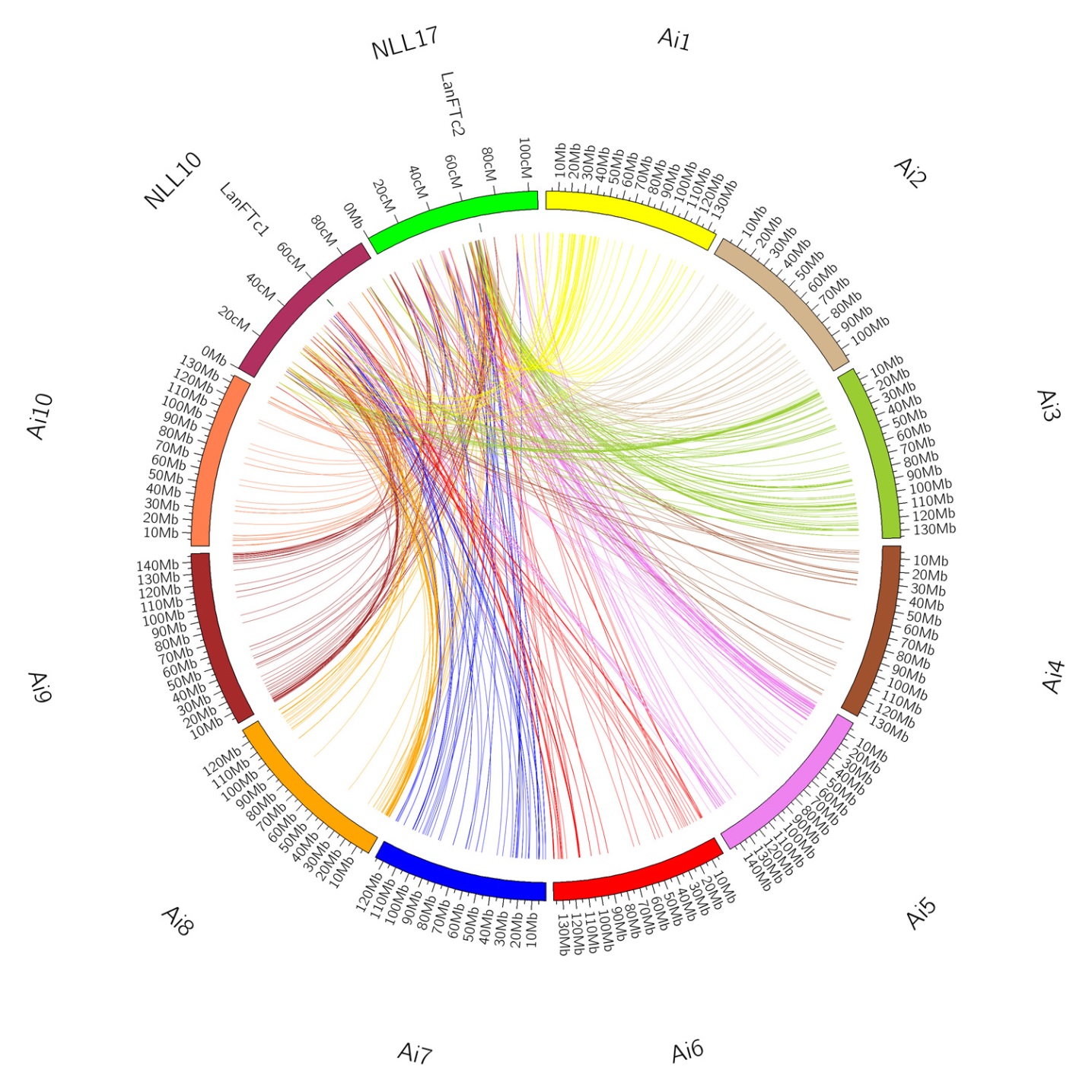


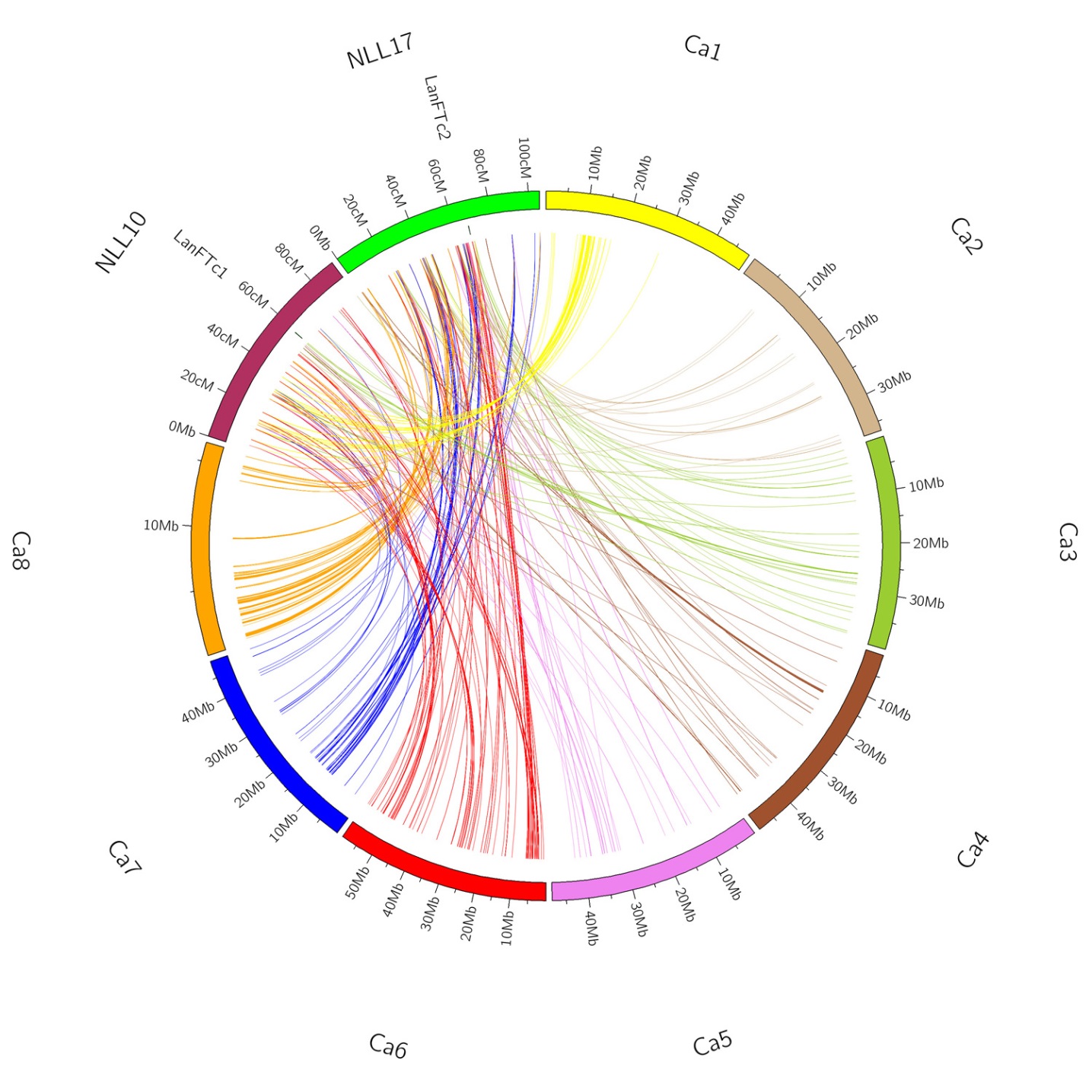


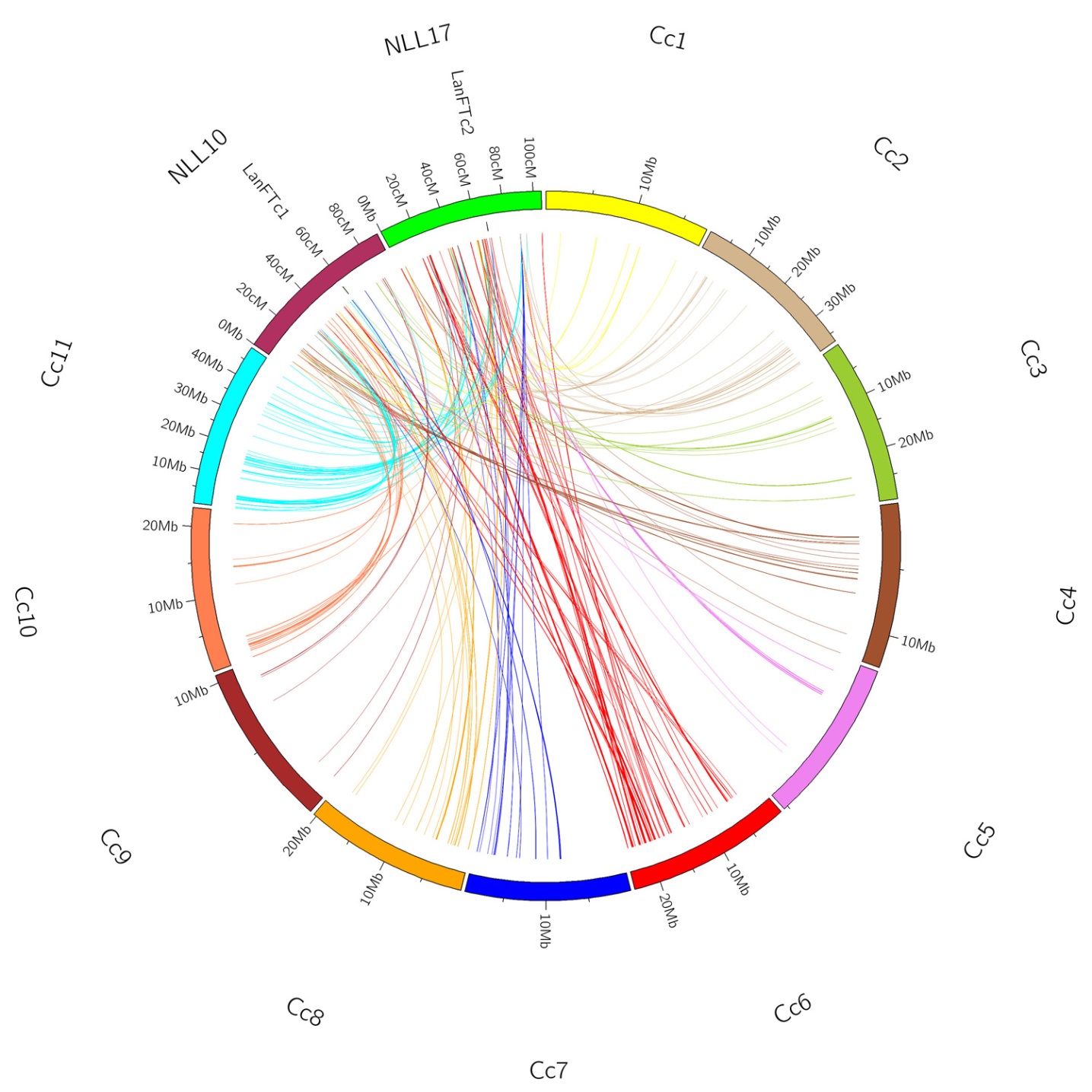


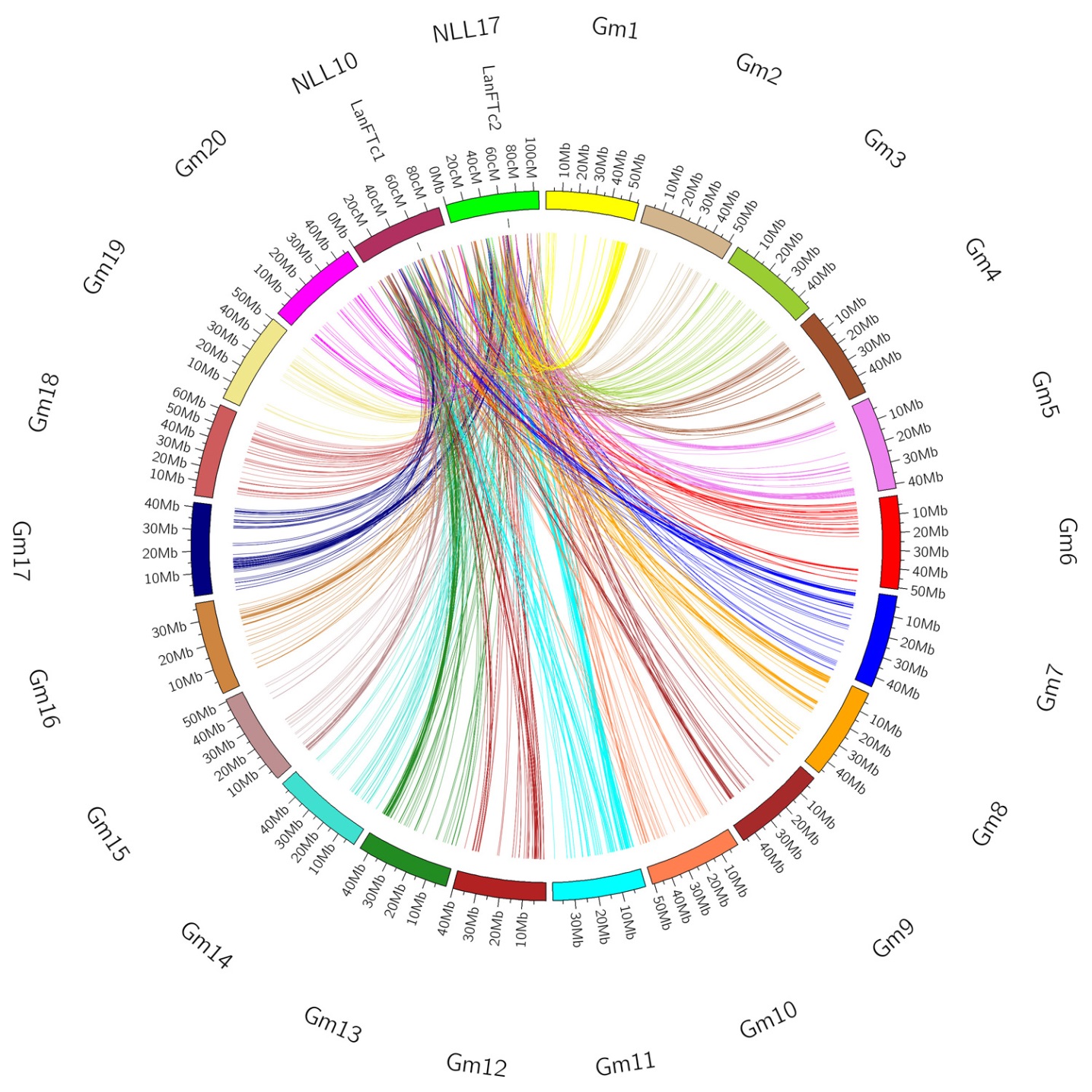


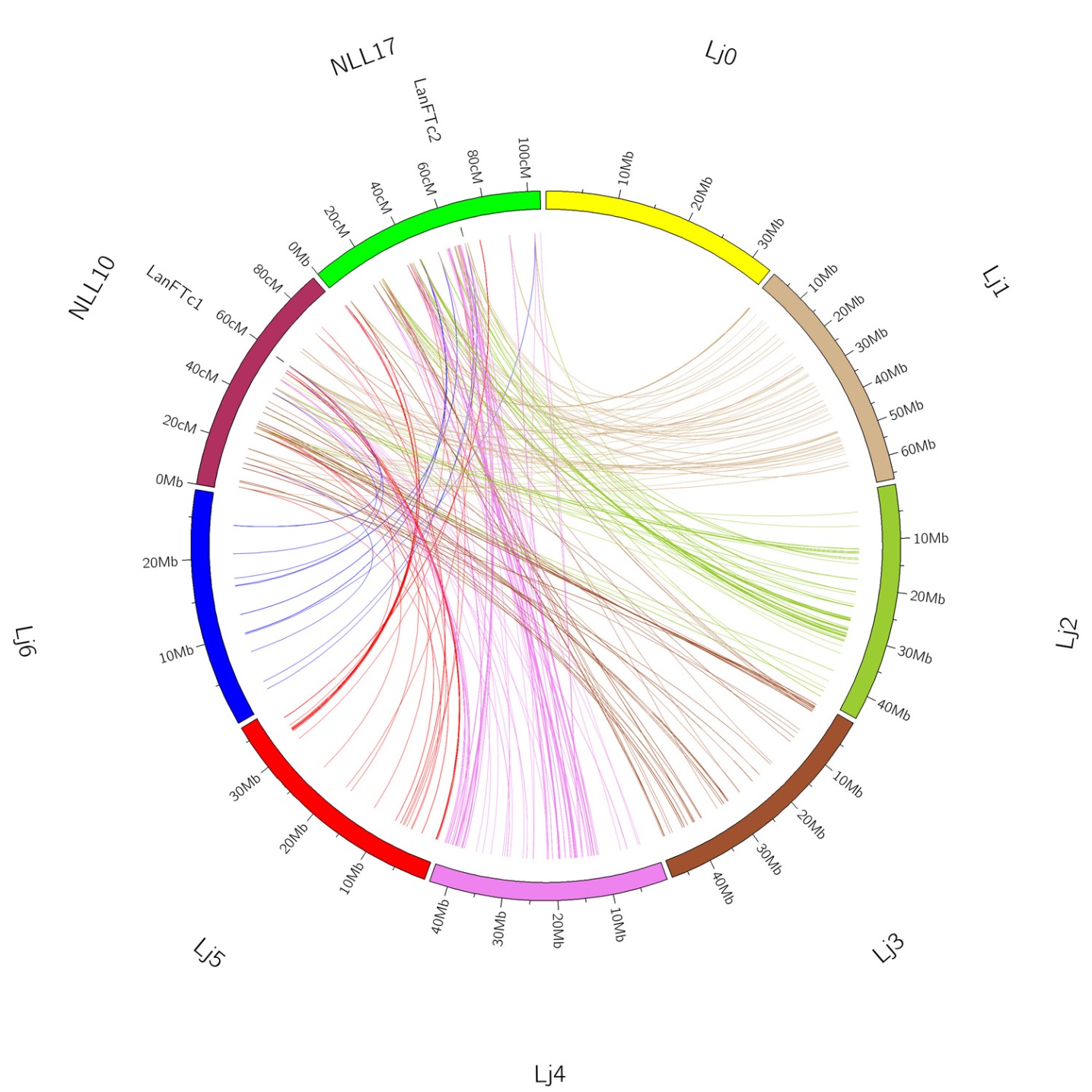


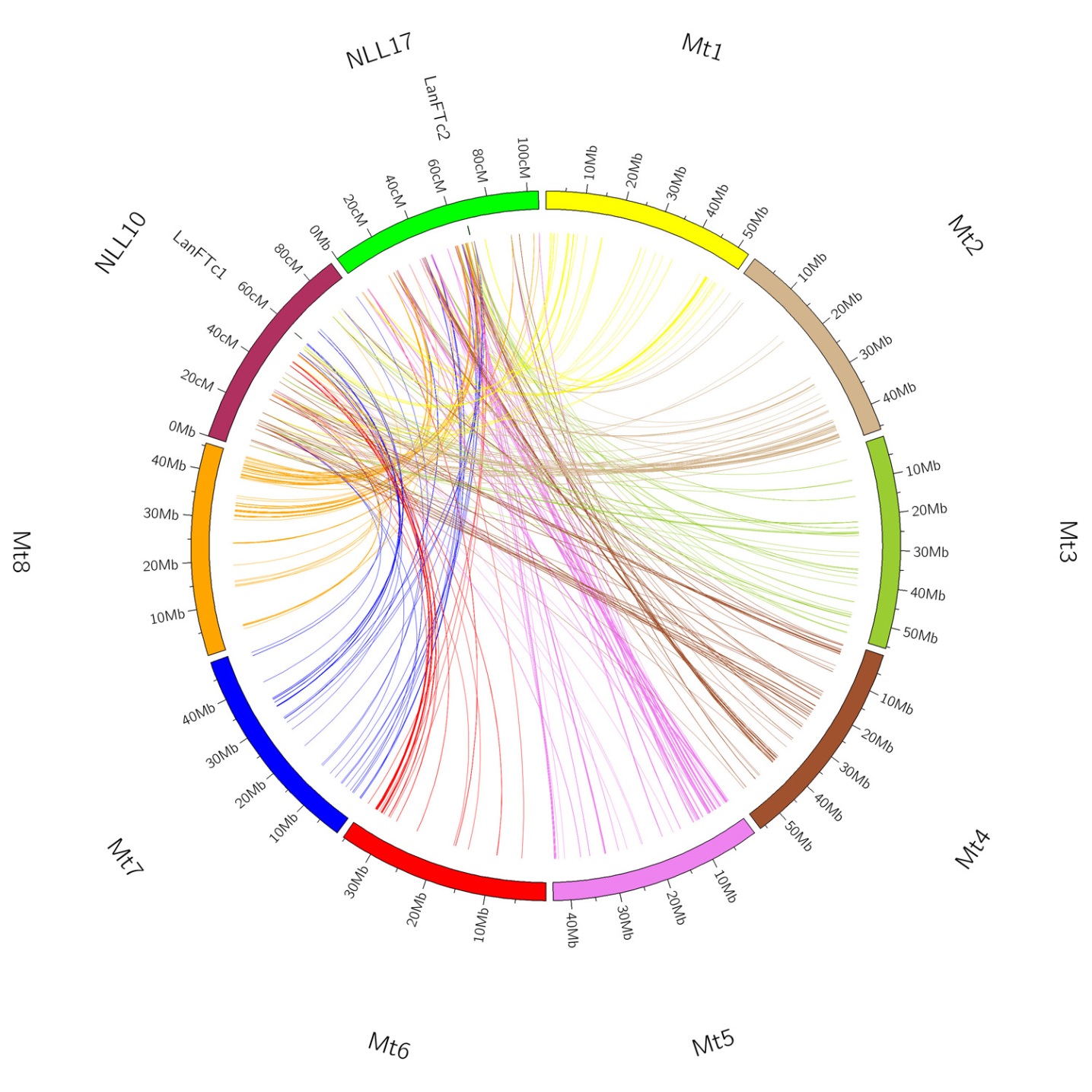


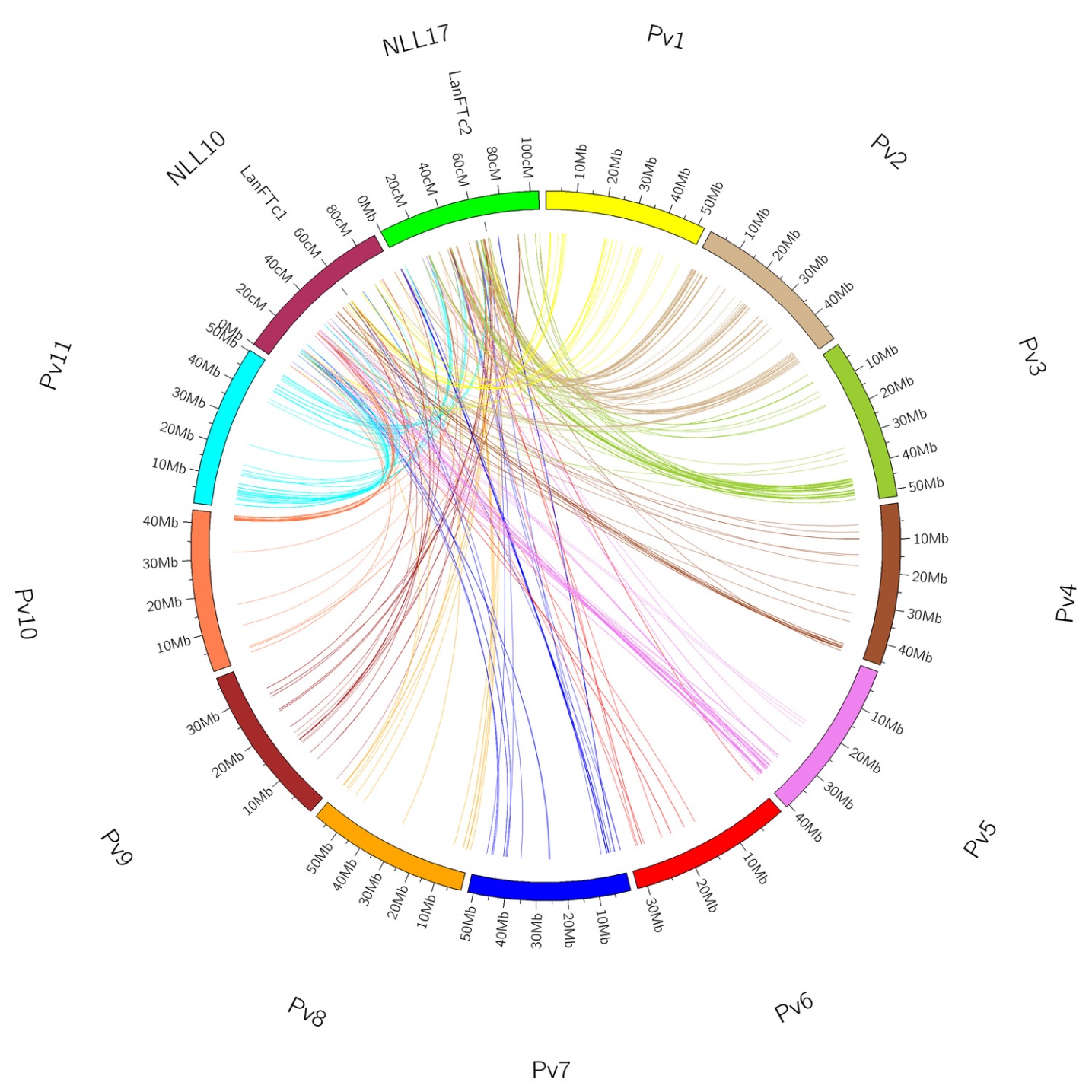


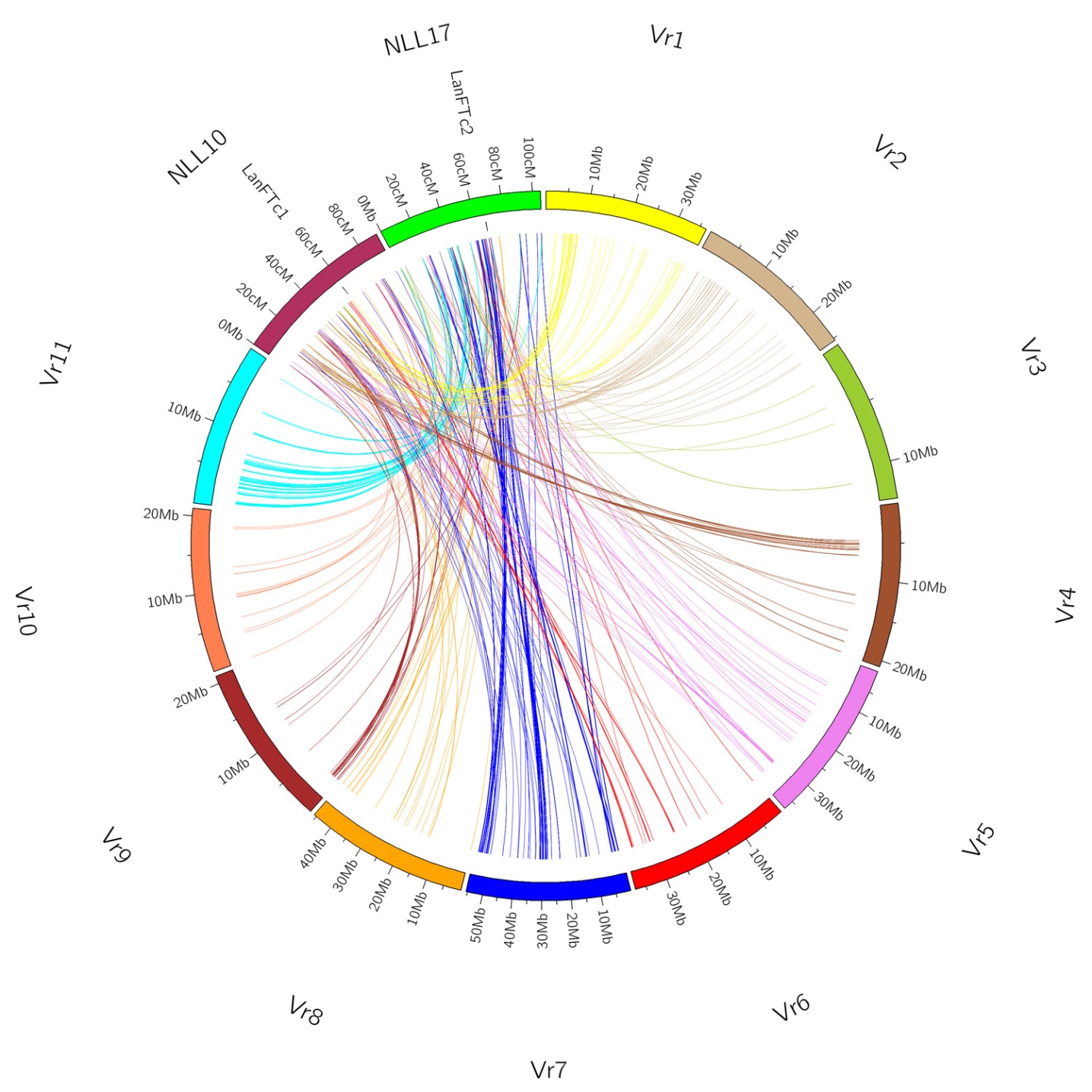

Supplement: Additional file 7: — Sequence homology links identified between linkage groups NLL-10 and NLL-17 and legume genomes. (DOC 3709 kb) [file 12864_2016_3150_MOESM7_ESM.doc]
