# Supplementary material for: Expansion of the phosphatidylethanolamine binding protein family in legumes: a case study of Lupinus angustifolius L. FLOWERING LOCUS T homologs, LanFTc1 and LanFTc2
Source: BMC Genomics. 2016 Oct 21;17:820. doi: 10.1186/s12864-016-3150-z (PMC5073747; doi:10.1186/s12864-016-3150-z)
Supplement: Additional file 10: — The set of 123 PEBP-family protein coding sequences used for phylogenetic inference. (DOC 95 kb) [file 12864_2016_3150_MOESM10_ESM.doc]

The set of 123 PEBP-family protein coding sequences used for phylogenetic inference

>Ad02_511142_516089_EHZ9Y.1_Ad_FTa1a ATGGGAGTTACTTACAATAATAGAGATGTTTGCAATGGCTGTGAATTCAAGCCTTCTCAAGTTGTCCACCAACCAAGAGTAGCCATTGGTGGAGTTGACCTCAGGAGCCTCTACACTCTGGTTGCTGTGAATCCAGATGCCCCTAGCCCCAGCAACCCTAGTATGAGGGAACACCTACATTGGCTGGTAACTGATATTCCCGCCCCAACTGGACCTAGTTTTGGTAACGAAGTGGTAGCATATGAAAGTCCACGACCAACATCAGGGATTCATCGAATAGTGTTGGTGTTATTTTGTCAATTGGGTAGGGAGAAGGTTTATGCCCCTGGTTGGCGCCAAAACTTCAACACCAGAGAATTTGCGGAACTATACAATCTTGGTTCGCCTGTTGCTGCTCTCTATTATAACATCCAAAGGGAGAATGGCTCTGGTGGCAGGAGGTTATAT

>Ad02_92805248_92807229_F950M.1_Ad_TFL1c ATGGCAAAGATGTCATCTTCAGATCCTTTAGTTCTTGGAAGAGTGGTTGGAGATGTCATCCACTCTTTCAATCCAAGTGTTCAAATGTCTGTCACTTACAACACCAAACAAGTCTTCAATGGCCATGAGTTCTTCCCTTCTGCTCTTACCACTAGGCCTAAGGTTGCCATTGATGGTGGTGACATGAGGACTTTTTACACACTGATCATGACAGATCCTGATGTTCCTGGCCCAAGTGATCCTTATCTGAGAGAACATTTGCACTGGATGGTTACAGACATTCCTGGCACAACAGATGCCACATTTGGTAGGCAGAGTGTAACCCCACCAAGTTCAAGGGATCACTTCAACACTATCAACTTCTCTGCTCACTCTGACCTTTCTCTTCCTGTTGCTGCTGTCTACTTCAATGCTCAGAGAGAAACCGCCGCTAGAAGACGC

>Ad03_127988156_127986875_Z8JSI.1_Ad_MFTa ATGGGAGCTTCTGTTGATCCTCTGGTAGTAGGGCGGGTGATTGGGGACGTGATAGACATGTTCGTTCCGTCTGTAGGGATGTCCGTCTACTACGGCTCAAAGCACGTGACCAATGGGTGCGACATCAAGCCTTCCATGGCTACAAGCCCACCCAAGGTTACTCTCACTGGCGGCAACATTCACAGCCTCTACACCCTCGTTATGACTGATCCAGATGCACCAAGTCCTAGTGAACCAACCATGCGCGAGTGGCTTCATTGGGTCGTTGTTGACATTCCTGGCGGTACAAATCCCACCCAAGGAAAGGAGATAGTAGCTTATATGGGACCTCGGCCACCTGTGGGAATTCACCGATACATACTGATATTGTTTGAGCAGAAGGAAGTTTTAGGGGGAGTGGAACAACCAGCTGCACGCGCGAGTTTCAACACTCGCTATTTTGCCAGACAATTCAATTTAGGACTTCCGGTCGCCACAGTTTATTTCAACTCCCAGAAAGAGCCTGCTTCTAAGAGACGT

>Ad04_123229387_123230945_A9H9T.1_Ad_FTb1b ATGTCCTCGTCATCTTCAAGAGCAAGAGCAAGTAGTTTATCATTAGGTCATAATGATCCTCTTGTTCTAGGCCGTGTAATTGGTGATGTTTTAGATCCCTTTACAAGTTCTGTCAACATGAGGGTCGTTTACGGCAATAACATTCAAGAGGTTATCAATTGTTGTGAATTAAGACCCTCCCAGATCATCAATAAACCAAGAGTTGAAGTTGGTGGACATGACCTTAGGACATTTTACACTCTGATCATGGTGGATCCTGATGCACCTAGTCCATGTAATCCAAGCGAGAGAGAATATTTGCACTGGCTGGTAATCAATATTCCCGAAACTACAGAAGCAAACTTTGGAGAAGAGATAGTGCCGTACGAAAGTCCACGACCAACAGCAGGGATTCATCGTATCGTGTTTGTGCTGTTCCGTCAGACTGGGAGACAGAGCGTTCATGGTCCTGGGTGGCGTCAAAATTTCAACACTCGAGATTTTGCTGAGTTCTATAATCTTGGATTGCCAGTTTCAGCTTTGTATTTCATCTGTAAACGA

>Ad04_123255004_123255997_1I7E9.1_Ad_FTb1a ATGCCTAGGTCAACGAACCCTCTAGTTATTTCACGTGTAGTAGGAGATGTTTTGGAGCCATTCACAAGTTCTGTTGGTATGAGGATTGTCTATGAAGGTAACGCTGAAGTTGTTAACTGTTCGGAGCTCAAGCCATCCCAAATTATCAACCAACCAAGAGTTGACGTTGGTGGAGATGATCTTAGAACCCTTTACACTCTGATCCTTGTGGACCCTGATGCACCTAGCCCAGGTGATCCAAATATGAGAGAATATTTGCACTGGATGGTAACAAATATTCCCGCAACAACAGGAGCAACCTATGGAGAAGAGATTGTGGCGTATGAGAATCCACGACCCGTAGTAGGGATTCATCGCTTAGTGTTTGTGCTGTTTCGGCAAATGGGGAGGCAAACTATATATGCTCCGGGATGGCGGCAAAGCTTCAACACCAGAGACTTTGCGGAGCTTTACAACCTGGGATCACCTGTTGCTGCCATATTTTTCAACTGCAAACGAGAAAACTCTCCTACTGGATCTTCAAGGAGGAGA

>Ad04_123341559_123244346_A4ISL.1_Ad_FTb1c ATGGATCCTCTTACTGTTGGAGGGGTAATCGGAGATGTTGTTGATCCTTTTGTAAGTTGTGTTTCTCTGAGGATTGTATATCACAATAACTCAGAAGTTGTTAATAGTTTGGAGTTGAAGCCATCCCAAATTATCAACCAACCAAGAGTTGACGTTGGTGGTGATGATTTTAGAACTTTTTACACTCTGATAATGGTGGACCCTGATGCACCTAGTCCTACTCATCCAAATATGAGAGAGTATTTGCACTGGTTGGTAATGAATATCCCAGGGACGACAGGAACGAGCTTCGGACAAGAGATAGTTGGATACGAAAGTCCAAGACCAATAGCAGGAATACATAGAATGGTGTTTGTGTTGTTCAAACAAATGAGGAGGCAAGAAGTGATACACCCTCCAAGATGGCGTAACAACTTCAACACTCGTGACTTCGCACAAGTGTACAATCTTGGATCTCCTGTTGCTGCTCTCTACTTCAATTGCCAGCGGGAGAGCGGTTGGGGTGGAAGGAGAATCATCATT

>Ad05_106417716_106416731_P7QP3.1_Ad_BFT ATGTCTAGGAATATGATGGAACCACTTGCTGTGGGAAGAGTGATAGGAGATGTGGTTGATGTATTCAACCCAAGTGTGAGAATGAATGTGATTTATTCCACTAAGCAAGTTGCTAATGGTCATGAGCTTATGCCTTCAACCCTTGTGCCCAAGCCTAGAGTTGAGATTGGTGGTCATGACTTAAGGGATGCTTATACCTTGATTATGACAGACCCGGATGCTCCAAGTCCTAGTGATCCATACTTGAGGGAACATCTTCACTGGATGGTTGTAGATATTCCTGGCACCACTGATGCATCTTTTGGGAGAGAGATAGTGGAGTATGAGAATCCAAGGCCAGTGATAGGAATACACAGATATGTGTTCATATTGTTGAAGCAGCATAGAGGGAGACAAACAGTGAGTCCCCCGGCTTCGAGAGAACAATTCAACACAAGAATCTTCTCTCAGGAAAATGGCCTTGGCCTACCTGTTGCTGCTGTTTACTTCAATGCTCAAAGAGAAACTGCTGCTAGGAGAAGG

>Ad06_91254893_91252935_80YRY.1_Ad_FTa2a ATGACTAGTGGTGGTAGCAGAAACTCTCTTGTTGTTGGAAGAGTGATTGGGGATGTGTTGGACCCTTTTCAAAGCACAATCCCTGTGAGAGTATCGTACGGTAATAGAGATGTAACAAATGGCTGTGAGCTTAAGCCTTCTCATGTATTCAACCAGCCTAGAGTTACTATTGGTGGAGATGACCTCAGGAATTTCTACACCCTGGTTATGGTTGATCCAGATGCACCTAGTCCAAGTAATCCTAATTGTAGAGAGTACCTCCATTGGTTGGTGACCGATATTCCAGCAACAACAGGAGTTAATTTTGGTAATGTGATAGTGAACTATGAAAAACCACTACCAAGCATGGGGATTCATCGTTTTGTGTTTGTGTTGTTTCGACAACCGGGAAGACAAACAGTATATGCTCCTGGATGGAGACAAAACTTCAACACAAGAGAGTTTGCTGAGCTTTATAATCTTAACTCGCCTGTTGCTGCTCTCTTCTTTAACTGTCAAAGGGAGTCTGGATCTGGTGGAAGAACAATGATGAAT

>Ad08_28294197_28293232_RJP5K.1_Ad_TFL1a ATGGCTTCATCAAATGCATTTTCATCATCAGATCCACTTACTATAGGAAGGGTGATCGGAGATGTAGTCGATGATTTCACAGAAACTGTGAAAATGACAGTCACTTACAACAACAAGCATGTCTACAATGGCTATGAGTTCTTCCCTTCTTCAGTTTCCACTAAACCTAGGGTTCATATTCATGGAGGCGACTTGAGATCCTCTTTCACTCTGATTATGACAGATCCAGATGTTCCTGGTCCTAGTGACCCATACTTAAGAGAACACTTACACTGGATGGTGACTGATATCCCAGGCACAACGGATGCAACATTTGGAAAGGAGGTGATAAAGTATGAGATGCCAAAGCCAAACATAGGAATACATAGATTTGTTTTGGTGCTTTACAAGCAGAAGAGGAGGCAGACGGTGAACAAGATTCCCAGTTCAAGGGACCTCTTCAACACAAGGAAATTCGCAGTTGAAAATGATCTTGGCACTCCTGTGGCTGCTGTCTTCTTCAATGCTCAAAGAGAAACCGCTTGCAGAAAGCGT

>Ad08_479244_476377_A6WCN.1_Ad_FTb1d ATGGACCCTCTTACTCTTGGAAGGTTGTTAGGGGATGTTGTTGATCCCTTTACAAGTTCTGTATCTTTGAGGATTGCGTATAACAATAACTCAGAAGTAGTCAATTGTTCTGACTTCAAGCCCTCCCAAATTACCAAACAACCAAGAGTTGATGTTGGTGGTGATGATTTTAGGACTTTTTACACTCTGATTATGGTAGACCCTGATGCACCTAGTCCTACTAATCCCAATATGAGGGAGTATTTGCATTGGTTGGTAATTAACATTCCAGGAACTACAGGGGCGAGCTTCGGGCAAGAGATAATGAAGTATGAGAGTCCAGAACCAACGGCAGGAATACATAGAATAGTGTTTGTGCTGTACAAGCAAATGGTGGGGAGGCAACTGATACACGCTCCGACCTGGCGTAACAACTTTAACACTCGCGACTTTGCTCAAGTTTACAATCTCGGATCTCCTGTTGCTGCTGTGTACTTCAATTGCCAGCGGGAGAGTGGTTGTGGTGGAAGGAGAATGATC

>Ad10_107219392_107221052_YY72S.1_Ad_FTa1b ATGCCACTTGTTAGTAAGGACCCTCTTGTAGTTGGGCGTGTGATTGGGGATGTTTTAGACCCCTTTGAAACTTCTATTTCAATTAGAGTTTCTTACAATAATAGAGATGTTTGCAATGGCTGTGAATTCAAGCCTTCTCAAGTTATCCACCAACCAAGAGTAGTCATTGGTGGAGATGACCTCAGGAACCTCTACACTCTGGTTGCAGTGAATCCAGATGCCCCTAGCCCAAGCGACCCTAGTTTGAGGGAATATCTACATTGGCTGGTAAGTAATGAGGTGGTAGCATATGAAAGTCCACGACCAACATCTGGGATTCATCGAATCGTGTTTGTGTTATTTCGTCAATTGGGTAAGGAGAAGGTTTATGCCCCTGGTTGGCGCCAAAACTTCAACACCAGAGAATTTGCCGAACTATACAATCGTGGTTCGCCTGTTGCTGCTCTCTATTATAACATCCAAAGGGAGAATGGTTCTGGTGGCAGGAGGTTATAT

>Ad10_3680794_3682428_QIZ46.1_Ad_MFTb ATGGGTAGGTCAACTGGGGCACTGTCCGTCGGCAAGGTCATTGGAGAAGTTGTCGACCCCTTCACTCCGACCGCTGAAATCACAGTTATCTACGGCTCCAAACAGGTGGCTAACGGTTGTGAAATCAAACCTTCTCTAGCAGCTGATAAACCCCATGTTCAAATTCTAGGTTCCTCTAATAATGCTGCCCATCTCTACACTCTGGTAATGGTGGATCCTGATGCTCCAAGTCCTAGTGAACCATCATACAGAGAGTGGCTCCACTGGATGGTTGTGGATATCCCTAATGGTTCTGATGTCACTCAAGGGAAGGAGTTGGTGACGTACACAGGACCATGTCCACCGGTGGGAATTCACCGCTACGTTTTTGCAGTATTTAATCAAAGTAATGAAGGCCCAACAACTCAAATTGGGCCTCCAAATGGACGGCCCAATTTCAATACTCGCCAGTTTGCTTCCCAACACTATCTTGGGCCTCCGGTTGCCGCTATATACTTCAATTCTCAGAAGGAACCTAAGACTAGGAAACGTCAA

>Ad10_73502142_73499326_G0NJW.1_Ad_FTc ATGGCATCATCAAGGGATTACGGAGACCCTCTTCTTGTTGGTGGTATAATTGGGGATGTTCTGAACCCTTTTACGAGCACAGTTTCTCTTAAGGTTCTCATTAACAACAGAGAGATTAACAATGGCTGTGAACTGAGGCCCTCTCATGTTGTTAACCGCCCTAGGATTACTATTGGTGGTGAAGACCTCAGGACCTTCTACACACTGGTTATGGTGGATGCTGATGCACCTAGCCCTAGTAACCCTTTCTTGAGGGAATACTTGCACTGGATGGTGACAGATATTCCGGCTACCACAAACACCACCTTCGGGATTCACCGATTTATAATTGTATTATTCAAGCAACTGGGAAGAGACACTGTTTTCCCCCCTGAATGGCGTCACAATTTCAACACCAGAGATTTTGCTTGCAACAATAGCTTGGCTCCTGTTGCTGCAGTTTATTTCAACTGTCAAAGGGAGCGTGGTTGTGGTGGAAGAAGGATGGAA

>Ai02_107500650_107502159_344D4.1_Ai_TFL1c ATGGCAAAGATGTCATCTTCAGATCCTTTAGTTCTTGGAAGAGTGGTTGGAGATGTCATCCACTCTTTCAATCCAAGTGTCCAAATGTCTGTCACTTACAACACCAAACAAGTCTTCAATGGCCATGAGTTCTTCCCTTCTGCTCTTACCACTAGGCCTAAGGTTGCAATTGATGGTGGTGACATGAGGACTTTCTACACACTGATCATGACAGATCCTGATGTTCCTGGCCCAAGTGATCCTTATCTGAGAGAACATTTGCACTGGTATGTGTTTGTGTTGTTGAAGCAAAAGAGAAGGCAGAGTGTAACCCCACCAAGTTCAAGGGATCACTTCAACACTATCAACTTCTCTGCTCACTCTGACCTTTCTCTTCCTGTTGCTGCTGTCTATTTCAATGCTCAGAGAGAAACCGCCGCTAGAAGACGC

>Ai03_128603664_128602352_WF9GZ.1_Ai_MFTa ATGGGAGCTTCGGTTGATCCTCTGGTAGTAGGGCGGGTGATTGGAGACGTGATAGACATGTTCGTTCCATCTGTAGGGATGTCTGTCTACTACGGCTCAAAGCACGTGACCAATGGTTGCGACATCAAGCCTTCCATGGCTACAAACCCACCCAAGGTTACTCTCACCGGCGGCAACATTCACAGCCTCTACACCCTCGTTATGACTGATCCAGATGCACCAAGTCCTAGTGAACCAACCATGCGCGAGTGGCTTCATTGGGTCGTTGTTGACATTCCTGGCGGTGCAAATCCCAACCAAGGAAAGGAGATAGTAGCTTATATGGGACCTCGGCCACCTGTGGGAATTCACCGATACATACTGATATTGTTTGAGCAGAAGGGAGTTTTGGGAGGAGTGGAACAACCAGCTACACGCGCGAACTTCAACACTCGCTATTTTGCCAGACAATTCAATTTAGGACTTCCGGTGGCTACAGTTTATTTCAACTCCCAGAAAGAGCCTGCTTCTAAGAGACGT

>Ai04_133275154_133276586_PC28F.1_Ai_FTb1b ATGTCCTCGTCATCATCAAGAGCAAGATCAAGTAGTTTATCATTAGGTCATAATGATCCTCTTGTTCTAGGCCGTGTAATTGGGGATGTTTTGGATCCCTTTACAAGTTCTGTCAACATGAGGGTTGTTTACGGCAATAACATTCAAGAGGTTATCAATTGCTGTGAATTCAGACCCTCCCAGATCATCAATAAACCAAGAGTTGAAGTTGGTGGACATGACCTTAGGACATTTTACACTCTGATCATGGTGGATCCTGATGCACCTAGTCCATGTAATCCAAACGAGAGAGAATATTTGCACTGGCTGGTAATCAATATTCCCGAAACTACAGAAGCAAACTTTGGAGAAGAGATAGTGCCGTACGAAAGTCCACGACCAACAGCAGGGATTCATCGTATTGTGTTTGTGCTGTTCCGTCAGATTGGGAGACATAGCGTTCATGGTCCTGGGTGGCGTCAAAATTTCAACACTCGAGATTTTGCTGAGTTCTATAATCTTGGATTGCCAGTTTCAGCTTTGTATTTCATCTGTAAACGA

>Ai04_133292440_133295229_U9HL1.1_Ai_FTb1c ATGGACCCTCTTGCTGTTGGAGGGGTAATCGGAGATGTTGTTGATCCTTTTGTAAGTTGTGTTTCTCTGAGGATTGTATATCACAATAACTCAGAAGTTGTTAATAGTTTGGAGTTGAAGCCATCCCAAATTATCAATCAACCAAGAGTTGACGTTGGTGGTGATGATTTTAGAACTTTTTACACTCTGATAATGGTGGACCCTGATGCACCTAGTCCTACTAATCCAAATATGAGAGAGTATTTACACTGGTTGGTAATGAATATTCCAGGGACGACAGGAACGAGCTTCGGACAAGAGATAGTTGGATACGAAAGCCCAAGACCAATAGCAGGAATACATAGGATGGTGTTTGTGTTGTACAAACAAATGAGGAGGCAAGAAGTGATACACCCTCCAAGATGGCGTAACAACTTCAACACTCGTGACTTCGCACAAGTCTACAATCTTGGAACTCCTGTTGCTGCTCTCTACTTCAATTGCCAGCGGGAGAGCGGTTGGGGTGGAAGGAGAATCATCATT

>Ai04_133309691_133310808_T1SIZ.1_Ai_FTb1a ATGCCTAGGTCAACGAACCCTCTAGTTATTTCACGTGTAGTAGGAGATGTTTTGGAGCCATTCACAAGTTCTGTGGGTATGAGGATTGTCTATGAAGGTAACGTTGAAGTTGTTAACTGTTCCGAGCTCAAGCCATCCCAAATTATCAACCAACCAAGAGTTGACGTTGGTGGAGATGATCTTAGAACCCTTTACACTCTGATCCTTGTGGACCCAGATGCACCTAGCCCAGGTGATCCAAATATGAGAGAATATTTGCACTGGATGGTAACAAATATTCCCGCAACAACAGGGGCAACCTATGGAGAAGAGGTTGTGGCGTATGAGAATCCACGACCCGTAGTAGGGATTCATCGCTTGGTGTTTGTGCTGTTTCGGCAAATGGGGAGGCAAACTATATATGCTCCGGGATGGCGGCAAAGCTTCAACACCAGAGACTTTGCAGAGCTTTACAACCTGGGATCACCCGTTGCTGCCATATTTTTCAACTGCAAACGAGAAAACTCTCCTACTGGATCTTCAAGGAGGAGA

>Ai05_133863102_133869395_YA5YU.1_Ai_FTa1c ATGCCAATTGTTAGTAAGGACCCTCTTGTAGTTGGGCGCGTGATCGGGGATGTTTTAGACCCCTTTGAAAGTTGTATTCCAATTAGAGTTACTTACAATAATAGAGATGTTTGCAATGGCTGTGAGTTCAAGCCTTCTCAAGTTGTCCACCAACCCAGAGTAGCCATTGGTGGAGATGACCTCAGGATCCTCTACACTCTGGTTGCTGTGAACCCAGATGCCCCTAGCCCTAGTGACCCTAGTTTGAGGGAATATCTACATTGGCTGATAACCGATATTCCCGCCACAACTGGACCTAGTTTTGGTAGCGAGGTGGTAGCATATGAAAGTCCACGACCAACTTTAGGAATTCATCGAATCGTGTTTGTGTTATTTCGTCAAATGGGTAGAGAGAAGATTTATGCCCCAGGTTGGCGCCAAAACTTTAACACAAGAGAATTTGCCGAACTATATAATCTTAGCTCGCCTGTTGCTGCTCTCTATTATAACATCCAGAGGGAGAATGGCTCTGGTGGCAGGAGGTTATAT

>Ai05_146699379_146698388_A5PDN.1_Ai_BFT ATGTCTAGGAGTATGATGGAACCACTTGCTGTGGGAAGAGTGATAGGAGATGTGGTTGATGTATTCAACCCAAGTGTGAGAATGAATGTGATTTATTCCACTAAGCAAGTTGCTAATGGTCATGAGCTTATGCCTTCAACCCTTGTGCCCAAGCCTAGAGTTGAGATTGGTGGTCATGACTTAAGGGATGCTTATACCTTGATTATGACAGACCCAGATGCTCCAAGTCCTAGTGATCCATATTTGAGGGAACATCTTCACTGGATGGTTGTAGATATTCCTGGCACCACTGATGCATCTTTTGGGAGAGAGATAGTGGAGTATGAGAGTCCAAGGCCAATGATAGGAATACACAGATATGTGTTCATATTGTTGAAGCAGCATAGAGGGAGACAAACAGTGAGTCCCCCTGCTTCGAGAGAGCAATTCAACACAAGAATCTTCTCACAGGAAAATGGCCTTGGCCTACCTGTTGCTGCTGTTTACTTCAATGCTCAAAGAGAAACTGCTGCTAGGAGAAGG

>Ai06_113913752_113909661_ZJ9GZ.1_Ai_FTc ATGGCATCATCAAGGGATTACGGGGACCCTCTTCTTGTTGGTGGTATAATTGGGGATGTTCTGAACCCTTTTACTAGCTCAGTTTCTCTTAAGGTTCTCATTAACAACAGAGAGATTAACAATGGCTGTGAACTGAGGCCCTCTCATGTTGTTAACCGCCCTAGGATTACTATTGGTGGTGAAGACCTCAGGACCTTCTACACACTGGTTATGGTGGATGCTGATGCACCTAGCCCTAGTAACCCTTTCTTGAGGGAATACTTGCACTGGATGGTGACAGATATTCCGGCTACCACAAACACCACCTTCGGGATTCACCGATTTATAGTTGTATTATTCAAGCAACTGGGAAGAGACACTGTTTTTCCCCCTGAATGGCGTCACAATTTCAATACCAGAGATTTTGCTTGCAATAATAGCTTGGCTCCTGTTGCCGCAGTTTATTTCAACTGTCAAAGGGAGCGTGGTTGTGGTGGAAGAAGAATGGAA

>Ai06_114073428_114074831_V23ZE.1_Ai_FTa2a ATGCCTAGTGGTGGTAGCAGGAACTCTCTTGTTGTTGGAAGAGTGATTGGGGATGTGTTGGACCCTTTTCAAAGCACAATGCCTATGAGAATATCATACGGTAATAGAGATGTAACCAATGGCTGTGAGCTTAAGCCTTCTCATGTAGTCAACCAGCCTAGAGTTACTATTGGTGGAGATGACCTCAGGAATTTCTACACCCTGGTAAGAACAATGGTCATAACAATATTTTGTAATGTGATAGTGAACTACGAAAAGCCACTACCAACCATGGGGATTCATCGTTTTGTGTTTGTGTTGTTTCGACAACCGGGAAGACAAACAGTATATGCTCCTGGATGGAGACAAAACTTCAACACAAGAGAGTTTGCTGAGCTTTATAATCTTAGTTCACCTGTTGCTGCTCTCTTCTTTAACTGTCAAAGGGAGTCTGGATCTGGTGGAAGAACAATGATGAAT

>Ai06_86558154_86556848_4V81G.1_Ai_TFL1b ATGTCATCAGAGCCTTTAATTGTAGGGAGAGTGGTAGGAGATGTTCTTGATCCATTCAATGCAAGCATAAGGATGAGTGTTAGTTATGGGAACAGGCAAGTGTACAATGGCCATGAGTTCTTCCCCTCCACAGTCACCTTCAAGCCCAAGGTTGAGATAGGTGGAACTGAATTGAGGCCCTTCTTTACACTGGTCATGACTGATCCGGATGTTCCTGGCCCTAGTGATCCTTATCTCAGAGAGCACTTGCATTGGATAGTGACAGACATTCCAGGCACAACAGATGCCACATTTGGTAGGTCTCTCATTCCTCTTTTGATATCTATGGATACTACAAGACTGAGAAATAATAATAATGTTGTTGATCCTAACATTGGGATCCATAGGTATGTTTTTGTGCTCTTCAAGCAAAAACGAAGGCAGTGTGTTACCACCATTCCTTCTTGCAGGGATCACTTCAACACCCGCACTTTCGCCGCCGACAACGACCTCGGCCTCCCTGTCGCTGCCGTCTACTTCAATGCTCAGAGGGAAACCGCTGCCAGGAGGCGT

>Ai07_107562219_107564533_03WUR.1_Ai_FTb1d ATGGACCCTCTTACTCTTGGAAGGTTGTTAGGAGATGTTGTTGATCCCTTTACAAGTTCTGTGTCTTTGAGGATTGCGTATAACAATAACTCAGAAGTAGTCAATTGTTCTGACTTCAAACCCTCCCAAATTACCAAACAACCAAGAGTTGATGTTGGTGGTGATGATTTTAGGACCTTTTACACTCTGATTATGGTAGACCCTGATGCACCTAGTCCTACTAATCCCAATATGAGGGAGTATTTGCATTGGTTGGTAATTAACATTCCAGGAACTACAGGGGCGAGCTTCGGGCAAGAGATAATGAAGTACGAGAGTCCAGAACCAACGGCAGGAATACATAGAATAGTGTTTGTGCTGTACAAGCAAATGGTGGGGAGGCAACTGATACACGCTCCGACGTGGCGTAACAACTTTAACACTCGCGACTTTGCTCAAGTTTACAATCTCGGATCTCCTGTTGCTGCTGTGTACTTCAATTGCCACCGGGAGAGTGGTTGTGGTGGAAGGAGAATGATC

>Ai08_5523249_5522289_T6XJY.1_Ai_TFL1a ATGGCTTCATCAAATGCATTTTCATCATCAGATCCACTTACTATAGGGAGGGTGATTGGAGATGTAGTCGATGATTTCACAGAAACCGTGAAAATGACAGTCACTTACAACAACAAGCATGTCTACAATGGCTTTGAGTTCTTCCCTTCTTCAATTTCCGCTAAGCCTAGGGTTCACATTCATGGAGGCGACTTGAGATCCTCTTTCACTCTGATTATGACAGATCCAGATGTTCCTGGTCCTAGTGACCCATACTTAAGAGAACACTTACACTGGATGGTGACTGATATCCCAGGGACAACGGATGCAACATTTGGAAAGGAGGTGATAAAGTATGAGATGCCAAAGCCAAACATAGGAATACATAGATTTGTTTTGGTGCTTTACAAGCAGAAGAGGAGGCAAACAGTGAGCAAGGTTCCCAATTCAAGGGACCTCTTCAACACAAGGAAATTCGTAGTTGAAAATGATCTTGGAACTCCTGTGGCTGCTGTCTTCTTCAATGCTCAAAGAGAAACCGCTTGTAGGAAGCGC

>Ai09_12053430_12050862_WWI38.1_Ai_FTa1a ATGCCAATTGTTAGTAAGGACCCTCTTGTAGTTGGGCGCGTGATTGGGGATGTTTTAGACCCCTTTGAAAGTTCTATTCCAATTAGAGTTACTTACAATAATAGAGATGTTTGCAATGGCTGTGAATTCAAGCCTTCTCAAGTTGTCCACCAACCAAGAGTAGCCATTGGTGGAGTTGACCTCAGAAACCTCTACACTCTGGTTGCTGTGAATCCAGATGCCCCTAGCCCCAGCAACCCTAGTTTGAGGGAACACCTACATTGGCTGGTAACTGATATTCCTGCCACAACTGGACCTAGTTTTGGTAACGAAGTGGTAGCATATGAAAGTCCACGACCAACGTCAGGAATTCATCGAATCGTGTTTGTATTATTTCGTCAATTGGGTAGGGAGAAGGTTTATGCCCCTGGTTGGCGCCAAAATTTCAACACGAGAGAATTTGCGGAACTATACAATCTTGGTTCGCCTGTTGCTGCTCTCTATTATAACATCCAAAGGGAGAATGGCTCTGGTGGCAGGAGATTATATTCATCATCATCA

>Ai10_133990482_133992025_VEP8T.1_Ai_FTa1b ATGCCACTTGTTAGTAAGGACCCTCTTGTAGTTGGGCGTGTGATTGGGGATGTTTTAGACCCCTTTGAAAGTTCTATTTCAATTAGAGTTAGTTACAATAATAGAGATGTTTGCAATGGCTGTGAATTCAAGCCTTCTCAAGTTGTCCACCAACCAAGAGTAGCCATTGGTGGAGATGACCTCAGGAACCTCTACACTCTGGTTGCAGTGAACCCAGATGCCCCTAGCCCAAGCGACCCTAGTTTGAGGGAATATCTACATTGGCTGGTAACTGATATTCCCGCCACAACTGGACCTAATTTTGGTAATGAGGTGGTAGCATATGAAAGTCCACGACCAACATCTGGGATTCATCGAATCGTATTCGTGTTATTTCGTCAATTGGGTAAGGAGAAGGTTTATGCCCCTGGTTGGCGCCAAAACTTCAACACCAGAGAATTTGCCGAACTATACAATCGTGGTTCGCCTGTTGCTGCTCTCTATTATAACATCCAAAGGGAGAATGGCTCTGGTGGAAGGAGGTTATAT

>Ai10_5493124_5494822_A8S33.1_Ai_MFTb ATGGGTAGGTCAACTGGGGCACTGTCCGTCGGCAAGGTCATCGGAGAAGTTGTCGACCCCTTCACTCCGATCGCTGAAATCACAGTGATCTACGACTCCAAACAGGTGGCTAACGGTTGTCAAATCAAACCTTCTCTAACAGCTGATAAACCCTATGTTCGAATTCTAGGTCCCTCTAATAATGCTGCCCATCTCTACACTCTGGTAATGGTGGATCCTGATGCTCCAAGTCCTAGCGAACCAACATATAGAGAGTGGCTCCACTGGATGGTTGTGGATATCCCTAATGGTTCTGATGTCACTCAAGGGAAGGAGTTGGTGACTTACACAGGACCATGTCCACCGGTGGGAATTCACCGCTACGTGTTTGCGGCATTTAATCAAAGTAATGAAGGCCCAACAACTCAAATTGGGCCTCCAAATGGTCGGCCCAATTTCAATACTCGCCAGTTTGCTTCCCAACACTATCTTGGGCCTCCGGTTGCTGCTATATACTTCAATTCTCAGAAGGAACCTAAGACTAGGAAACGTCAA

>AtBFT.AT5G62040_At_BFT ATGTCAAGAGAAATAGAGCCACTAATAGTGGGAAGAGTGATAGGAGATGTACTCGAAATGTTTAATCCAAGTGTGACAATGAGAGTCACTTTCAATTCCAACACAATCGTATCCAATGGTCACGAGCTCGCGCCTTCTCTTCTCCTCTCTAAGCCTCGCGTTGAGATCGGTGGCCAAGATCTTCGTTCCTTCTTCACCTTAATCATGATGGACCCCGATGCCCCGAGTCCTAGTAATCCTTATATGCGTGAATATCTGCATTGGATGGTGACAGATATTCCCGGGACAACCGATGCTTCTTTTGGGAGAGAGATAGTGAGATATGAGACGCCTAAACCGGTGGCGGGAATACACAGATACGTCTTTGCGCTATTCAAACAGAGAGGGAGGCAAGCTGTGAAGGCAGCGCCGGAAACTAGAGAGTGTTTCAACACAAACGCTTTCTCTTCTTACTTTGGTCTTTCTCAACCTGTTGCTGCTGTTTACTTCAACGCCCAACGTGAAACTGCTCCTCGACGACGTCCTTCTTAT

>AtFT.AT1G65480_At_FT ATGTCTATAAATATAAGAGACCCTCTTATAGTAAGCAGAGTTGTTGGAGACGTTCTTGATCCGTTTAATAGATCAATCACTCTAAAGGTTACTTATGGCCAAAGAGAGGTGACTAATGGCTTGGATCTAAGGCCTTCTCAGGTTCAAAACAAGCCAAGAGTTGAGATTGGTGGAGAAGACCTCAGGAACTTCTATACTTTGGTTATGGTGGATCCAGATGTTCCAAGTCCTAGCAACCCTCACCTCCGAGAATATCTCCATTGGTTGGTGACTGATATCCCTGCTACAACTGGAACAACCTTTGGCAATGAGATTGTGTGTTACGAAAATCCAAGTCCCACTGCAGGAATTCATCGTGTCGTGTTTATATTGTTTCGACAGCTTGGCAGGCAAACAGTGTATGCACCAGGGTGGCGCCAGAACTTCAACACTCGCGAGTTTGCTGAGATCTACAATCTCGGCCTTCCCGTGGCCGCAGTTTTCTACAATTGTCAGAGGGAGAGTGGCTGCGGAGGAAGAAGACTT

>AtMFT.AT1G18100_At_MFT ATGGCGGCTTCTGTTGATCCTTTGGTGGTCGGAAGAGTGATCGGAGATGTGTTGGACATGTTCATCCCAACCGCCAATATGTCTGTCTACTTTGGCCCCAAACACATCACTAACGGCTGCGAGATCAAACCCTCCACCGCAGTCAATCCTCCAAAAGTCAACATCTCGGGCCATTCCGATGAGCTTTACACTCTCGTGATGACTGACCCGGACGCACCTAGCCCAAGCGAGCCGAACATGAGAGAATGGGTCCACTGGATTGTCGTGGATATTCCCGGAGGCACAAATCCCTCAAGAGGAAAAGAGATACTTCCATACATGGAACCAAGGCCACCAGTGGGGATTCACCGTTACATATTGGTACTTTTCCGGCAAAACTCACCGGTGGGTCTGATGGTGCAGCAGCCTCCATCACGAGCCAATTTCAGCACACGAATGTTCGCTGGACATTTCGATCTTGGTCTACCTGTGGCCACTGTCTATTTCAACGCCCAAAAGGAACCTGCTTCACGCAGACGC

>AtTFL1_AT5G03840_At_TFL1 ATGGAGAATATGGGAACTAGAGTGATAGAGCCATTGATAATGGGGAGAGTGGTAGGAGATGTTCTTGATTTCTTCACTCCAACAACTAAGATGAATGTTAGTTATAACAAGAAGCAAGTCTCCAATGGCCATGAGCTCTTTCCTTCTTCTGTTTCCTCCAAGCCTAGGGTTGAGATCCATGGTGGTGATCTCAGATCCTTCTTCACTTTGGTGATGATAGACCCAGATGTTCCAGGTCCTAGTGACCCCTTTCTAAAAGAACACCTGCACTGGATCGTTACAAACATTCCCGGCACAACAGATGCTACGTTTGGCAAAGAGGTGGTGAGCTATGAATTGCCAAGGCCAAGCATAGGGATACATAGGTTTGTGTTTGTTCTGTTCAGGCAGAAGCAAAGACGTGTTATCTTTCCTAATATCCCTTCGAGAGATCACTTCAACACTCGTAAATTTGCGGTCGAGTATGATCTTGGTCTCCCTGTCGCGGCCGTCTTCTTTAACGCACAAAGAGAAACCGCTGCACGCAAACGC

>AtTSF_NM_118156.1_At_TSF ATGTCTTTAAGTCGTAGAGATCCTCTTGTGGTCGGCAGTGTTGTTGGAGATGTTCTTGATCCTTTCACGAGGTTGGTCTCTCTTAAGGTCACTTATGGCCATAGAGAGGTTACTAATGGCTTGGATCTAAGGCCTTCTCAAGTTCTGAACAAACCAATAGTGGAGATTGGAGGAGACGACTTCAGAAATTTCTACACCTTGGTTATGGTGGATCCAGATGTGCCGAGTCCAAGCAACCCTCACCAACGAGAATATCTCCACTGGTTGGTGACTGATATACCTGCCACCACTGGAAATGCCTTTGGCAATGAGGTGGTGTGCTACGAGAGTCCACGTCCCCCCTCGGGAATTCATCGTATTGTGTTGGTATTGTTCCGGCAACTCGGAAGACAAACGGTTTATGCACCGGGGTGGCGCCAACAGTTCAACACTCGTGAGTTTGCTGAGATCTACAATCTTGGTCTTCCTGTGGCTGCCTCTTACTTCAACTGCCAGAGGGAGAATGGCTGTGGGGGAAGAAGAACG

>Ca01_14759516_14761088_Ca_07054_Ca_TFL1a1 ATGAATATTGTTGTATTAGCAGATCCTCTTGTAATAGGAAGAGTGGTGGGAGATGTAGTTGATTATTTCAACACAACTGTGAAAATGTCTGTCACTTACAACAACACTAAGCAAGTTTACAATGGTCATGAGTTTTTTCCTTCTTCTCTTACCATTAAGCCTAAGGTTCATATTCATGGTGGTGATATGAGATCTTTCTTCACTTTGATCATGACAGATCCAGATGTCCCTGGCCCTAGTGATCCATACTTGAAGGAACACTTACACTGGATAGTCACAGATATACCGGGCACAACAGATGCTACATTTGGGAAGGAAGTGATAAAGTATGAAATGCCACGTCCAAACATAGGAATACATAGGTTTGTGTTCATCCTGTACAAACAAAAGCGAAGACAGACAGTGATGAAAATACCAACATCAAGAGATCATTTCAACACTAAGAAATTTGCAGAAGACAATGACCTTGGACCTCCTGTCGCTGCTGTTTTTTTCAATGCTCAAAGAGAAACTGCTGCTAGAAGACGT

>Ca01_14775699_14777349_Ca_07052_Ca_TFL1a2 ATGAATATTGTTGTATTAGCAGATCCTCTTGTAATAGGAAGAGTGGTGGGAGATGTAGTTGATTATTTCAACACAACTGTGAAAATGTCTGTCACTTACAACAACACTAAGCAAGTTTACAATGGTCATGAGTTTTTTCCTTCTTCTCTTACCATTAAGCCTAAGGTTCATATTCATGGTGGTGATATGAGATCTTTCTTCACTTTGATCATGACAGATCCAGATGTCCCTGGCCCTAGTGATCCATACTTGAAGGAACACTTACACTGGATAGTTACAGATATACCAGGTACAACGGATGCCACATTCGGGAAAGAAGTGATAAAGTACGAAATGCCACGTCCAAACATAGGAATACATAGGTTTGTGTTCATCCTGTACAAACAAAAGCGAAGACAGACAGTGATGAAAATACCAACATCAAGAGATCATTTCAACACTAAGAAATTTGCAGAAGACAATGACCTTGGTCCTCCTGTCGCTGCTGTTTTTTTCAATGCTCAAAGAGAAACTGCTGCTAGAAGACGT

>Ca02_6939948_6937956_Ca_19141_Ca_FTa1a ATGCCTAGTGGTAGTACTAGTAGAGACCCTCTTGTTGTTGGGGGTGTAATTGGTGATGTATTGGACCTCTTTCAAACTTCTATTCCTATAAGAGTCACATATAATGGTAAAGATGTTACCAATGGTTGTGAATTCAAACCTTCACAAGTTGTTAATCAACCAAGAGTTAGTGTTGGTGGAGATGACCTCAGAAACTTCTACACACTGATCATGGTGGATCCAGATGCACCAAGCCCTAGCAATCCAAATTTGAGAGAGTATCTTCATTGGTTGGTGACTGATATTCCTGCAACTACTGGACCTACTTTTGGTAATGAGGTTGTAACTTACGAAAATCCGCGGCCATTTATGGGAATTCATCGAATAATCTTCGTGGTTTTTCAACAACTTGGTAGAGAGACTGTGTATGCTCCGGGATGGCGCCAAAATTTCAACACAAGAGAATTTGCAGAACTTTACAATTTGGGATTACCAGTTTCTTCTGTTTATTATAACATTCAAAGGGAAGCTGGCTCTGGTGGAAGAAGGTTATGC

>Ca02_776679_779528_Ca_17002_Ca_FTb2a ATGAACCCTCTAGTTGTTGGTCGTGTAATTGGAGATGTTTTGGATAACTTTACAGATTCTGTGTCTCTAAGGGTTATTTATGACAATAACAAAGAAGTCATCAACAGTGGTGAGCTCAAACCCTCCCAAATTGTCAACCCACCAAGAGTTCAAGTTGGTGGAAATGACTTCAGGACTCTATACACTCTGGTCATGGTAGACCCTGATGCACCAAGCCCTAGTGACCCAAATATGAGGGAATACCTGTATTGGATGGTGACCAACATTCCAGCGACTACAGGGACAACATTCGGTCAAGAGATAGTGAGCTATGAAAATCCAAGACCAACATCAGGGATTCATCGTGTGGTTTTTGTGTTGTTTCGACAACCTTGTAGACACACAATATTAGCTCCAGGGTGGCGCCAGAATTTCGTCACTAGAGATTTTGCTGAAGTTTATAATCTTGGTTTACCTGTTGCTGCTCTCTATTTCAATGTTCAACGAGAAACTGGTTCTGGTGGAAGGAGGATGATCATA

>Ca03_26393854_26397368_Ca_FTa2a ATGGCCAGTGGTAGCCGAAATCCTCTTGTTGTTGGGCGTGTAATAGGGGATGTATTAGACAACTTTGAAAATTCAATTCCTCTTCGAGTCACCTATGGTAATAGGGAGGTTAATAATGGTTGTGAGCTTAAACCTTCTCAAGTTGCCAATCAACCCAGAGCGAGTGTTGGTGGAAATGACATGAGGAACTTCTACACCCTAGTTTTGGTGGATCCTGATTCACCTAGCCCAAGTAACCCCACTTTTAGAGAGTACCTTCATTGGTTGGTGACTGATATTCCAGCAACTACTGGGGTTAGTTTCGGTAATGAGGTTGTAGGTTATGAAAGGCCACGACCCACCTCTGGAATTCATCGTTTTGTGTTTGTATTGTTTCGTCAACAATGCAGACAAAGAGTGTATGCTCCAGGATGGAGACAAAATTTTAATACAAGAGAATTTGCTGAACTCTACAACTTAGGATTGCCTGTTGCAGCTGTTTTCTTCAATTGTCAAAGGGAGAGTGGCTCTGGTGGAAGAACATTTAGA

>Ca03_26409508_26417785_Ca_FTa2b ATGGCCAGTGGTAGCAGAAATCCTCTTGTTGTTGGGCGTGTAATAGGGGATGTATTAGACCCCTTTGAAAGTTCTATTCGTCTCCTAATCACCTACGGTAATAGGAATGTTAACAATGGTTGTGAGCTTAAACCTTCCCAAGTAGCCAAACAACCTCAAGTGAGTATAGGCGGAAACGATCTCAGGATCTTCTACACCCTGGTTTTAGTAGATCCAGATGCACCTAGTCCAAGTAACCCCAGTTTTAGGGAGTACCTTCATTGGTTGGTGACTGATATTCCTTCAACTGCTGGGGCTAGTTTTGGTAATGAAGTTGTATGTTACGAAAAACCACGACCCAACTTGGGGATACATCGTTACGTGTTTGTATTATTTCGTCAACAGTGTCAACAAGTAGTGTTTGCTCCTGGCTGGCGACAGAATTTCAATACAAGAGAATTCGCTGAACTTTACGATCTTGAATTGCCAGTTGCTGCTGTGTTCTTCAATTGTCAAAGGGAGACTGGCTCTGGGGGAAGAACCTTTAGA

>Ca03_26437711_26444880_Ca_08264_Ca_FTc ATGCCACGAAATAATGGGGGGGTCGACCCTCTTGTTGTAGGGGGTGTGATAGGAGATGTTTTGAATCCTTTTACAAACTCTGTATCTTTGAGTGTTGTCACCAATAACAAAGAGATTAGTAATGGCTGTGTGCTCAAGCCCTCTCAAGTAGTTAACCGCCCAAGGGCTAGTGTTGGTGGTGAAGATCTAAGGACTTTCTACACGCTTGTTATGGTGGACGCAGATGCCCCTAGCCCTAGTAACCCTGTTTTGAGGGAATACTTGCATTTGGACACTGTTTTTGCCCCAGAATGGCGTCATAATTTCAAGACTAGAAACTTTGCAGAAATTAACAATTTGGTCATTGTTGCATCGGTTTATTTCAATTGTCAAAGAGAGCGTGGTTGCGGTGGAAGGAGAAGC

>Ca03_35175096_35174282_Ca_00790_Ca_TFL1b ATGGCAAGAATGTCTCAAGAACCACTACTTGTTGGGAGAGTTATAGGAGAAGTTCTTGATTCATTTACCACAAGTATGAAAATGACTGTGAGTTACAACAAGAAGCAAGTCTTCAATGGCCATGAGTTCTTCCCTTCCACTATCAATATCAAACCTAAAGTTGAGATTGATGGTGGTGACATGAGATCCTTCTTTACATTGGTGATGACAGACCCTGATGTTCCTGGCCCTAGTGATCCTTATCTTAGAGAACACTTGCACTGGATTGTGACAGATATTCCAGGAACAACAGATGCCACATTTGGTAAAGAGTTGGTGAGCTATGAAATACCAAAACCAAACATAGGGATACACAGGTTTGTGTTTGTTCTGTTCAAACAAAAAAGCAGAGAATCAGTTATGACAACACCATCTTCAAGGGATCATTTCAACACACGCAATTTCGCTTCACAGAATGATCTTGGTCTTCCTGTTGCTGCTGTTTACTTCAATGCTCAGAGAGAAACCGCTGCAAGAAGACGC

>Ca06_2350077_2351609_Ca_10366_Ca_MFT ATGGCTGCCTCGGTTGATCCTTTGGTTGTTGGTCGTGTGATCGGAGATGTTGTTGACATGTTCATTCCATCTGTTGGCATGTCTGTTTATTTTGGTCCTAAACATGTCACTAATGGATGTGACATTAAGCCATCCATTGCTATCAACCCACCCAGAGTCACTCTCACTGGAAACATCGATAACCTCTACACTCTGGTTATGACTGATCCTGATGCACCAAGCCCCAGTGAACCAAGCATGCGCGAGTTGATACATTGGATCGTGGTTGACATCCCTGGAGGAACAAACCCAAAGCGAGGGAAGGAGATTCTGCCATACATAGGGCCAAAACCACCTGTGGGGATTCATCGATTTATTTTGGTTCTGTTTAAGCAGAAGGGACCAATGGGACTGGTGGAACAACCAACAAGTCGAGTGAGTTTCAACACTCGTTATTTTGCAAGTCAATTGGACCTTGGCCTTCCAGTAGCTACGGTCTACTTCAACTCTCAGAAGGAACCTCAGGCTAAGAGGCGT

>Ca08_12990114_12992073_Ca_20095_Ca_TFL1c1 ATGACTGATATATCTTTAGAACCTCTAGTTCTTGGGAAAGTGATAGGAGATGTTATTGATAATTTCACCCCAAGCATTAAAATGATTGTAACTTACAACAACAAAGAAATCTTCAATGGTTATGAGCCCTTTCCTTCTACAGTTAGCACAAGGCCAAGGGTTGAGATTCAAGGAGGGGACATGAGGTCCCTATTTACACTGATCATGATAGACCCGGACGTTCCTGGCCCAAGTGATCCTTATATGAGAGAACACTTGCACTGGATGGTGACTGACATTCCAGGCACAACAGATTCCACATTTGGTAAAGAGTTGACAAGCTATGAAATACCAAAGCCTAACATAGGGATCCATAGGTATGTGTTCGTCCTTTTCAAGCAAAAGAAAAAGCACTCAATTACTACTCCTTCTTCAAGGGATCACTTCAACACAAGAAGTTTTTCAATGCAAAATGACCTTGGTGTCCCTGTTGCTGCTGCTTATTTCAACGCAAGGAGGCCAACTGCTGCTAGAAAACCTACCTATATA

>Cascaffold1275_13210_11243_Ca_27934_Ca_TFL1c2 ATGGGAAGTATATCTTTAGATCCTCTAGTTCTTGGGAAAGTGATAGGAGATGTTATTGATAATTTCACCCCAAGCATTAAAATGATTGTAACTTACAACAACAAAGAAATCTTCAATGGTTATGAGCCCTTTCCTTCTACAGTTAGCACAAGGCCAAGGGTTGAGATTCAAGGAGGGGACATGAGGTCCCTATTTACACTGATCATGATAGACCCGGACGTTCCTGGCCCAAGTGATCCTTATATGAGAGAACACTTGCACTGGATGGTGACTGACATTCCAGGCACAACAGATTCCACATTTGGTAAAGAGTTGACAAGCTATGAAATACCAAAGCCTAACATAGGGATCCATAGGTATGTGTTCGTCCTTTTCAAGCAAAAGAAAAAGCACTCAATTACTACTCCTTCTTCAAGGGATCACTTCAACACAAGAAGTTTTTCAATGCAAAATGACCTTGGTGTCCCTGTTGCTGCTGCTTATTTCAATGCAAGGAGGCCAACTGCTGGTAGAAAACCTACCTATATA

>Cascaffold1324_486920_489093_Ca_19775_Ca_BFT ATGTCTAGGTCATTGGAACCACTTTCTATGGGAAGGGTGATAGGAGAAGTGGTTGATATATTTAATCCAAGTGTTAGAATGAATGTGACATATTCCACTAAGCAAGTTGCAAATGGTCATGAGTTATTGCCTTCTATTGTTATGAACAAACCAAGGGTTGAGATAGGTGGTGATGACTTGAGGACTGCTTATACTTTGATCATGACAGATCCAGATGCTCCTAGTCCTAGTGATCCATATTTAAGGGAACATCTCCATTGGATGGTTACAGATATTCCAGGTACCACAGATGTCTCTTTTGGGAAAGAAATTGTGGAGTATGAGAATCCAAAACCAGTTATAGGAATCCATAGATATGTCTTCATATTGTTCAAGCAAAGAGGGAGACAAACAGTGAGATCCCCAACTTTTAGAGACAATTTCAACACAAGGAGCTTTTCACAACAAAATAGCCTTGGCTTACCTGTTGCTGCAGTTTACTTCAATGCTCAAAGAGAAACTGCAGCAAGAAGAAGG

>Cc03_20698853_20697530_C.cajan_10074_Cc_TFL1b ATGGCAAGAATGCCTATAGAGCCTCTAATAGTGGGAAGAGTCATAGGAGAAGTTCTTGATTCTTTCACCACAAGCATAAAAATGACTGTGAGTTACAACAAGAAGCAAGTCTACAATGGCCATGAGCTCTTCCCTTCCACTGTCAACACCATACCCAAGGTTGAGATTGATGGTGGTGATATGAGGTCCTTCTTCACACTGATCATGACAGACCCGGATGTTCCTGGCCCTAGTGATCCTTATCTGAGAGAGCACTTGCACTGGATAGTGACAGATATTCCAGGCACAACAGATGCCACATTTGAGTTGGTGAGCTATGAGATCCCAAAGCCTAATATTGGGATCCATAGGTTTGTGTTTGTCCTGTTCAAGCAAAAGCGTAGGCAGTGTGTTACTCCACCTGCTTCAAGGGATCACTTCAACACACGCAATTTCGCCGCACAGAATGACCTTGGCCTCCCTGTGGCTGCTGTCTACTTCAATGCACAGAGGGAAACGGCTGCAAGAAGACGC

>Cc05_645708_647483_C.cajan_23132_Cc_MFT ATGGCTGCTTCCGTTGATCCCCTTGTGGTTGGTCGGGTTATTGGTGATGTGGTGGACATGTTTATCCCTTCAGTGAACATGTCTTTGTACTTTGGCTCCAAGCATGTCACCAATGGCTGTGACATCAAACCCTCCATAGCAATTAGCCCTCCCAAGATCACTCTCACAGGGAATATGGATAACCTTTATACCCTGGTTATGACCGATCCCGATGCACCAAGCCCCAGTGAACCAAGCATGCGGGAGTTGATTCATTGGATCGTGGTCAACATACCTGGAGGCACCAACCCCACAAGGGGTAAAGAGATTTTACCCTACTTGGGACCAAAGCCACCGGTAGGGATCCACCGCTTCATCTTTGTTCTGTTTCAGCAAAAGGGACCTTTGGCTGCTGTGGAGCAGCCAACAACTCGTGCGGGTTTCAACACTCGCTACTTCGCCAGCCAATTGGACTTGGGCCTTCCTGTGGCCACTGTCTATTTCAATGCTCAGAAAGAGCCTAAGAGGCGT

>Cc07_19111257_19108717_C.cajan_19134_Cc_FTb1a ATGCCTAGATCATTGGATCCTCTTGTTATTGGACGTGTAATAGGAGATGTCTTGGATCCTTTTACATGTTGTGTCTCTATGAGGATAATCTATAATAATAGCCATGAAGTTATCAACTGTTGTGAGCTCAAACCCTCACAAATCATCAACCAACCAAGAGTTGAAGTTGGTGGAGATGACCTCAGGAACTGTTACACCCTGGTCATGGTGGATCCTGATGCACCTAGTCCAGGAAATCCAAATCAGAGGGAATATTTGCACTGGTTAGTAGCCAACATTCCAGGAACCACAGGAGCAAACTTTGGTGAAGAAGTTGTTGGTTACGAAAGTCCAAGACCTATGTCGGGGATTCATCGTATTGTTTTCATACTGTTTCGTCAGACTGGAAGACAAACTATATATGCTCCTGGATGGCGCCAAAATTTCAACACCAGAGATTTTAGCGAGCTTTATAATCTTGGATTACCAGTTGCAGCAACCTACTTCAACTGTAAACGTCAAAATGTTTCTGCCAGAGATGGAACTGGAAGAGCCAGA

>Cc07_19330754_19335783_C.cajan_19156_Cc_FTb2a ATGCCTAGCTCAATGAACCCTCTTGTTGTTGGACGTGTAATAGGAGATGTTTTAGAGCCTTTTGCAAGTTCTGTCTCTATGAGAGTTGTCTACAACAATAACAAAGATGTCATGAACAGTGCTGAGCTCAAACCCTCCCAAATAATCAACCCTCCAAGAGTCGAGGTTGGTGGAAATGACCTCAGGACCCTTTACACCCTGGTCATGGTGGACCCTGATGCACCAAGCCCAAGTGACCCAAATATGAGGGAATATCTACACTGGTTGGTAACCAATATTCCAGCGACTACAGGGGCAACCTTTGGAGAAGAGGTTGTGAGCTATGAAAGTCCAAAACCAACATCAGGGATTCATCGTATAATTTTTGTGTTGTTTCGTCAACCGTGTAGACAACCTATACCTGCACCAGGATGGCGTCAAAATTTCATCACTAGAGATTTTGCAGAGTTTTACAATCTTGGATTACCAGTTGCAGCTGTCTATTTCAATTGTCAACGACAAGGTGGTTCTGGGGGAAGGAGAATTATG

>Cc08_7632709_7633785_C.cajan_16048_Cc_BFT ATGTCTAGACTCATGGAACCACTTGCTGTGGGAAGAGTGATAGGGGAAGTGGTTGACATTTTCACCCCAAGTGTGAGAATGAATGTGACATATTCCACCAAGCAAGTTGCTAATGGCCATGAGTTAATGCCTTCTACTATCGTGGCCAAGCCACGCGTAGAGATTGGTGGTGATGACATGAGGACTGCTTATACCTTGATCATGACAGACCCAGATGCTCCCAGTCCTAGTGATCCATATCTAAGGGAACATCTTCACTGGATGGTTACAGATATCCCTGGCACCACAGATGTCTCTTTTGGAAAAGAGATTGTGGGCTATGAGAGTCCAAAGCCAGTGATAGGAATCCACAGATATGTATTCATCCTGTTCAAGCAGAGAGGAAGACAAACAGTGAGACCTCCATCTTCAAGAGACTATTTCAACACGAGGAGGTTCTCAGAGGAGAATGGCCTTGGCCTACCAGTTGCTGCTGTTTACTTCAATGCTCAAAGAGAGACTGCTGCAAGGAGGAGG

>CcScaffold000409_635796_634148_C.cajan_26153_Cc_FTa2a ATGGCTAGTGGTAGCAGAAATCCTCTTGTTGTTGGGCGTGTTATTGGAGAAGTAATAGACCCCTTTGAAAGTTCTATTCCTTTTAGGGTCACCTATGGCAATAGAGAAGTGAGCAATGGTTGTGAGTTTAAACCTTCACAAGTTGTCAAGCAACCCAGAGTGAGTGTTGGCGGAGATGACTTCAGGAACTTCCACACTATGGTCTTAGTGGATCCTGATGCTCCTAGTCCAAGTAATCCCAATTGCAGGGAGTACCTTCATTGGTTGGTGACTGATATTCCAGCGACCACAGGGGCTAGTTTCGGTAACGAGGTTGTAAGTTATGAAAGTCCACGACCCACGATGGGGATTCATCGTTTCGTATTTGTGTTGTTTCGTCAACAATATCGACAGAGGGTGTATGCTCCTGGATGGAGACAAAATTTCAATACTAGAGAATTTGCTGAACTTTACAATCTCGGATTACCAGTTGCTGCTGTCTTCTTCAACTGTCAGAGGGAAGCTGGCTCCGGTGGTAGGACATTT

>CcScaffold132593_212899_214116_C.cajan_31760_Cc_TFL1a ATGAACATGATATCATCAGATCCTCTTGTTATTGGTAGGGTGATCGGAGATGTAGTGGATTATTTCACTCCAACTGTGAAAATGACTGTCACCTTCAACAATAAGCAGGTCTACAACGGTCATGAGTTTTTTCCCTCCTCAGTAACTACTAAGCCTAAGGTTCAGATTCATGAAGGCGATATGAGATCCTTCTTCACTCTGGTCATGACAGATCCAGATGTCCCTGGCCCTAGTGATCCATACCTGAGGGAACACTTACACTGGATGGTCACAGATATCCCAGGCACAACGGACGCCACATTTGGAACTGAGGTGGTGAAGTATGAAAATCCAAGGCCGAACATAGGGATCCATAGGTTTGTGTTCCTCCTTTTCAAGCAGAAGCGCAGGCAGGGAGTGATGAAAATACCAAGTTCAAGGGACCTCTTTAACGCGAGAAACTTTGCAGAGGAGAATGACCTGGGGCCTCCTGTGGCTGCTGTGTTTTTCAATGCTCAAAGGGAAACTGCTGCCAGAAGACGT

>CcScaffold137112_28219_23820_C.cajan_42877_Cc_FTa1a ATGCCTGGAGGAAGTAGGGACCCTCTGGTTGTAGCGCGTGTAATCGGGGATGTTTTGGACCCTTTTGAATGTTCTATTCCTATGAGGGTCACGTACGGTAATAAAGATGTCAGCAATGGATGTGAATTCAAACCCTCACAAGTTGGCAACCAACCAAGAATAAATATCGGTGGAGATGACCTCAGGAACTTCTACACTTTGATCGCTGTTGATCCTGATGCACCTAGCCCAAGTGACCCCTATTTGAGAGAATACCTCCATTGGTTGGTGACTGATATTCCAGCAACAACGGGGCCTAGTTTCGGTCATGAGGTTGTAACATATGAAAGTCCGCGACCTACGATGGGGATCCATCGTGTAGTCTTTGTGTTATTTCGGAAACTGGGCAGGGAGACAGTGTATGCACCTGGGTGGCGCCAGAATTTCAATACCAAAGAATTTGCTGAACTTTACAATCTTGGATTGCCAGTTGCTGCTGTCTATTTTAACATTCAGAGAGAATCTGGTTCTGGTGGAAGAAGGTTATAT

>CcScaffold137665_134613_135917_C.cajan_36529_Cc_TFL1c ATGGCAAGGGTGTCGACAGATTCTCTAATTATTGGGAGAGTCATAGGAGATGTTCTTGAATCTTTCACCCCAACCATAAAAATGACTGTAACTTTCAATAAAAAGCAACAAGTCTACAATGGCCATGAGTTCTTCCCTTCCACAGTCACCACAAGGCCAAGGGTTGAGATTGGAGGAGGTGATATGAGATCCTTCTTTACACTGATTATGACAGACCCGGATGTCCCTGGACCTAGTGATCCTTATCTGAGAGAGCATTTGCACTGGATGGTGACAGACATTCCAGGCACCACAAATGCCTCATTTGGCAATGTGTTGGTGAGCTATGAAGTGCCAAAGCCTAACATAGGGATACACAGGTATGTGTTTGTCCTGTTCAAGCAAAAACATAGGCAGTGTATAACGACTCCACCTTCTTCAAGGGATCACTTCAACACACGCAAATTCTCAGCTGAGAATGACCTTGGCCTCCCTGTTGCTGCTGTCTACTTCAATGCACAGAGGGAAACGGCTGCTAGAAGACGC

>GmBFTa.Glyma09g26550.Glyma.09G143500_Gm_BFTa ATGTCTAGGCTAATGGAACAACCACTTGTTGTGGGAAGAGTGATAGGAGAAGTGGTTGACATTTTCAGCCCAAGTGTAAGAATGAATGTTACATATTCCACTAAGCAAGTTGCTAATGGTCATGAGTTAATGCCTTCTACTATTATGGCCAAGCCACGCGTTGAGATTGGTGGTGATGACATGAGGACTGCTTATACCTTGATCATGACAGACCCAGATGCTCCAAGTCCTAGTGATCCACATCTGAGGGAACATCTCCACTGGACGGTTACAGATATCCCTGGCACCACAGATGTCTCTTTTGGTAAAGAGATAGTGGGCTATGAGAGTCCAAAACCAGTAATAGGAATCCACAGGTATGTGTTCATTTTGTTCAAGCAGAGAGGAAGACAGACAGTCAGGCCTCCTTCTTCAAGAGACCATTTCAACACAAGGAGGTTCTCAGAAGAGAATGGCCTTGGCCTACCAGTTGCTGTAGTTTACTTCAATGCTCAAAGAGAGACTGCCGCAAGAAGGAGG

>GmBFTb.Glyma16g32080.Glyma.16G196300_Gm_BFTb ATGTCTAGGCTCATGGAACCACTTGTTGTGGGAAGAGTGATAGGAGAAGTGGTCGACATTTTCAGCCCAAGTGTAAAAATGAATGTGACATATTCCACCAAGCAAGTTGCCAATGGTCATGAGTTAATGCCTTCTACTATTATGGCCAAGCCACGCGTTGAGATTGGTGGTGATGACATGAGGACTGCTTATACCTTGATCATGACTGACCCAGATGCTCCAAGTCCTAGTGACCCATGTCTAAGGGAACATCTCCACTGGATGGTTACAGATATCCCTGGCACCACAGATGTCTCTTTTGGAAAAGAGATTGTAGGCTATGAGAGTCCAAAGCCAGTAATAGGAATCCACAGGTATGTGTTCATCTTGTTCAAGCAGAGAGGAAGACAAACAGTGAGGCCTCCATCTTCAAGAGACCACTTCAACACAAGGAGGTTCTCAGAAGAGAATGGCCTTGGCCTACCAGTTGCTGCAGTTTACTTCAATGCTCAAAGAGAGACTGCTGCAAGAAGGAGG

>GmFTa1.Glyma16g04840.Glyma.16G044200_Gm_FTa2a ATGCCTAGTGGTAGTAGGAACCCTCTTGTTGTTGGGCGTGTTATAGGGGAAGTAATAGACCCCTTTGAAAGTTCTATTCCTTTCAGGGTGACCTATGGTAATAAAGAAGTGGGCAATGGTTGTGAGCTTAAACCTTCTCAAGTTCCCAACCAACCTAGAGTGAGTATTGGTGGAGATGATCTCAGGAAATTCTACACTATGGTCATGGTGGATCCTGATGCTCCTAGCCCAAGTAACCCTAATTTCAGAGAGTATCTTCATTGGTTGGTGACTGATATTCCCGAAACTACAGGGCCTAATTTCGGTAACGAGATCGTAAGCTATGAAAGCCCGCGACCCACGATGGGGATTCATCGTTTCGTGTTTGTGTTATTCCGTCAACAGTTTAGACAGAGGGTGTATGCTCCTGGATGGCGACAAAATTTCAATACTAGAGAATTTGCTGAACTTTACAACCTTGGATTGCCGGTTGCTGCTGTCTTCTTCAACTGTCAGAGGGAAACTGGCTCTGGTGGTAGAACATTT

>GmFTa2.Glyma19g28390.Glyma.19G108100_Gm_FTa2b ATGCCTGGCGGTAGTAGGAACCCTCTTGTTGTTGGGCGTGTTATAGGGGAAGTAATAGATCCCTTTGAAATTTCTATTCCTTTCAGGGTCACCTATGGTAATAGAGAAGTGGGCAATGGTTGTGAGCTTAAACCTTCCCAAGTTGCCAACCAACCCAGAGTGAGTGTTGGTGGAGATGACCTCAGGAACTTCTACACTATGGTCCTGGTGGATCCTGATGCTCCTAGCCCAAGTAACCCTAATTTCAGGGAGTACCTTCATTGGTTGGTGACTGATATTCCAGAAACTACAGGGCCTAATTTCGGTAACGAGGTTGTAAGCTATGAAAGCCCACGACCCACGATGGGGATTCATCGGTTGGTGTTTGTGTTATTCCGTCAACAGTTTAGACAGAGGGTGTATGCTCCTGGATGGCGACAAAATTTCAATACCAGAGAATTTGCTGAACTTTACAACCTTGGATTGCCGGTTGCTGCTGTCTTCTTCAACTGTCAGAGGGAAAGTGGCTCTGGTGGTAGAACATTT

>GmFTa3.Glyma16g26660.Glyma.16G150700_Gm_FTa1b ATGCCTAGTGGAAGTAGGGATCCTCTCGTTGTTGGGGGAGTAATTGGGGATGTATTGGATCCTTTTGAATATTCTATTCCTATGAGGGTTACCTACAATAACAGAGATGTCAGCAATGGATGTGAATTCAAACCCTCACAAGTTGTCAACCAACCAAGGGTAAATATCGGTGGTGATGACCTCAGGAACTTCTATACTTTGATTGCGGTTGATCCCGATGCACCTAGCCCAAGTGACCCCAATTTGAGAGAATACCTCCATTGGTTGGTGACTGATATCCCAGCAACAACAGGGGCTAGTTTCGGCCATGAGGTTGTAACATATGAAAGTCCAAGACCAATGATGGGGATTCATCGTTTGGTGTTTGTGTTATTTCGTCAACTGGGTAGGGAGACCGTGTATGCACCAGGATGGCGCCAGAATTTCAACACTAAAGAATTTGCTGAACTTTACAACCTTGGATTGCCAGTTGCTGCTGTCTATTTCAACATTCAGAGGGAATCTGGTTCTGGTGGAAGGAGGTTATAC

>GmFTa4.Glyma16g26690.Glyma.16G151000_Gm_FTa1a ATGCCTCGTGGAAGTAGGGACCCTCTAGTTGTTGGGCGTGTGATTGGGGATGTATTGGACCCTTTTGAATGTTCTATTCCTATGAGGGTCACCTACAATAACAAAGATGTCAGCAATGGATGTGAATTCAAACCCTCACAAGTTGTCAACCAACCAAGAATAAATATCGGTGGTGATGATTTCAGGAACTTCTACACTTTGATCGCGGTTGATCCTGATGCACCTAGCCCAAGTGATCCCAATTTCAGAGAATACCTCCATTGGTTAGTAACTGACATTCCAGCAACAACGGGGCCTACTTTCGGTCATGAGGTTGTAACATATGAAAATCCACGACCCATGATGGGGATCCATCGTATAGTCTTTGTGTTATTTCGTCAACAGGGTAGAGAGACAGTGTATGCACCAGGATGGCGCCAAAATTTCATTACTAGAGAATTTGCTGAACTTTACAATCTTGGATTGCCAGTTGCTGCTGTCTATTTTAACATCCAGAGAGAATCTGGTTGTGGTGGAAGAAGGCTATGT

>GmFTb1.Glyma08g47820.locusdeleted_Gm_FTb2a ATGGCTATTACAACGAACCCTCTTGTTGTTGGACGTGTAATAGGAGATGTTCTGGAGCCATTTGCAAGTTCTATCCCTTTGAGAGTTGTTTACAACAATAACAAAGAAGTCATCAACAGTGGAGAGCTCAAACCCTCCCAAATAATCAACCCTCCACGAGTTGAGGTTGGTGGTGATGACCTCAGGACCCTCTACACTCTGGTCATGGTGGACCCTGATGCACCCAGCCCAAGTGACCCAAATATGAGGGAATATCTGCACTGGTTGGTAACCAATATTCCAGCGACTACAAGTGCAAGCTTTGGACAAGAGGTTGTGAGCTATGAAAGTCCACGACCAACATCAGGGATTCATCGTTTCATATTTGTGTTGTTTCGTCAACCGAGAAGAATGTCTATACCTGCTCCAGGATGGCGCCAAAATTTCATCACTAGAGATTTTGCAGAGTATTATAATCTTGGCTTACCAGTTGCTGCTGTCTATTTCAATTGTCAACGACAAGGTGGTTCTGGGGGAAGGAGGCTAATGTTA

>GmFTb2.Glyma08g47810.Glyma.08G363100_Gm_FTb1c ATGCCTATATCAATGGACCCCCTTGTTCTTGGACGTATAATAGGAGATATTTTGGATCCCTTCACGAGTTCTGTTTCTCTGAGGGTTGTTTATAACAACCAGTCATCGGTTATCAATAGTTGTGAGTTCAAACCTTCCCAAATCGTCAACAAACCAAGAATTAATATTCGTGGAAATGACCTAGGGATCTTTTACACTCTGATAATGGTGAACCCCGATGCTCCTAGCCCAAGTGACCCACATATGAAGGAATATTTGCATTGGTTGGTAACCAATATTCCAGCATCTACAGGGGCAACCACTGGAGAAGAGATTGTGGAGTATGAAAGTCCACGACCAACTTCTGGGATTCATCGGATTGCTTTTGTGTTGTTTCGTCAATTTGATAGACAAATTGTGCATGCTCCAAGATGGCGCCAAAATTTCAACACTAGAGATTTTGCTGAGGTTTACAACCTTGGATCACCAGTTGCTGCTGTGTATTTCAACTGTCAACGTGAAGGTGGTTGGGGTGGACGAAGGAGA

>GmFTb3.Glyma18g53680.Glyma.18G298900_Gm_FTb1b ATGCCTAGATCAACGGACCCTCTTGTTATTGGAGGTGTAATAGGAGATGTTTTGGAGCCTTTCACAAGTAGCGTTTCTATGGGGATAGTCTATAATAATTGCCCTCAAGTTATCAACTGCTGTGAGCTCAAACCCTCCAAAATCCTCAATCGACCAAGAATTGAAATTGGTGGAGATGACCTCAGGACCTTTTACACCCTGGTTATGGTGGATCCTGATGCACCTAGCCCAGGCAATCCAACCCAGAGAGAATATTTGCACTGGTTGATAACCAATATTCCTGCAACTACAGGGGCAAACTTCGGAGAAGAGATTGTCTCCTATGAAAGTCCACGTCCAATAGTAGGGATTCATCGAATAGTTTTTGTGTTATTTCGTCAGCTGCGTAGACTAACTCTGCAACCTCCAGGCTGGCGCCAGAATTTCAACACTAGAGACTTTGCTGAGATTTATAATCTTGGATTACCAGTAGCGGCCATGTACTTCAACTGTAAACGAGAAAATGATCAAAGCAGTGGAAGAAGAAGA

>GmFTb4.Glyma18g53690.Glyma.18G299000_Gm_FTb1a ATGGACCCTCTTGTCATTGGACGTGTAGTAGGAGATGTTTTGGAGCCTTTCACTAGTTGCGTCTCTCTTAGGATTCTATATGACAGCTGCTCCGAAGTTATCAACTGCTGTGAGCTCAAACCCTTCCAAATCATCAACCAACCTAGAGTTGAAGTTGGTGGTGATGACTTCAGGACCTTTTACACCCTGGTAATGGTGGATCCTGATGCACCTAGCCCAGGAAATCCAAATCAGAGGGAATATTTGCACTGGTTGGTAACCAATATTCCAGGAACTACAGGAGCAAACTTCGGTGAAGAGGTTGTGAGCTATGAGAGTCCACGACCGATGATGGGAATTCATCGTATTATTTTCATATTATTTCGTCAGTCAGGTAGACAAACTATATATGCTCCAGGATGGCGTCAAAATTTCAACACGAGAGATTTCAGCGAGGTTTATAATCTTGGATTACCAGTGGCAGCAACCTACTTCAACTGTAAACGTCAAAATAATTCCGCAAGAGATGGAAGAAGGACA

>GmFTc1.Glyma16g04830.Glyma.16G044100_Gm_FTc1 ATGGCACGGGAGAACCCTCTTGTTATTGGTGGTGTGATTGGGGATGTTCTCAACCCTTTTACAAGCTCCGTTTCTTTGACTGTTTCAATCAATAATAGGGCGATTAGCAATGGCTTGGAACTCAGGCCCTCTCAAGTTGTTAATCGCCCTAGGGTTACTGTTGGTGGTGAAGACCTAAGGACCTTCTACACTCTGGTTATGGTGGATGCAGATGCACCTAGCCCTAGCAACCCTGTCTTGAGGGAATACCTTCACTGGATGGTGACAGATATTCCAGCTACCACAAATGCAAGCTTTGGGAGAGAGGTTGTGTTTTATGAGAGCCCGAACCCTTCAGTAGGGATTCATCGAATCGTGTTCGTATTGTTCCAGCAATTGGGCAGAGACACTGTCATCACCCCAGAATGGCGCCATAATTTCAATTCCAGAAACTTTGCTGAAATTAATAACCTTGCACCTGTTGCAGCAGCTTATGCCAACTGCCAAAGAGAGCGTGGTTGCGGTGGAAGGAGATAT

>GmFTc2.Glyma19g28400.Glyma.19G108200_Gm_FTc2 ATGGCACGGGAGAACCCTCTTGTTATTGGGGGTGTGATTGGGGATGTTCTCAATCCTTTTACAATCTCCGTTTCTTTTACTATTTCAATCAATAATAGGGCGATTAGCAATGGCTTGGAACTGAGGCCCTCTCAAGTTGTTAATCGCCCTAGAGTCACTGTTGGTGGTGAAGACCTAAGGACCTTCTACACACTGGTTATGGTGGATGCAGATGCACCTAGCCCTAGCAACCCTGTCTTGAGGGAATACCTTCACTGGATGGTGACAGATATTCCAGCTACCACAAATGCAAGCTTTGGGAGAGAGGTTGTGTTTTATGAGAGCCCGAACCCTTCAGCAGGGATTCATCGACTTGTGTTCATATTATTCCAGCAACTGGGCAGAGACACTGTCATCACCCCAGAATGGCGCCATAATTTCAATTCCAGAAACTTTGCTGAAATTAATAACCTTGCACCTGTTGCAGCAGCTTATGCCAACTGCCAAAGAGAGCGTGGTTGCGGTGGAAGGAGATAT

>GmMFTb.Glyma05g34030.Glyma.05G244100_Gm_MFT ATGCGTTATTTGTCTCTGAGTACTTTTTCTCTCTTGTGCATTACCTTTGTGGTCATGGCAGCCTCCGTGGATCCCCTAGTGGTTGGTCGCGTGATCGGCGATGTGGTAGACATGTTCATTCCTTCAGTCAACATGTCCGTTTACTTTGGGTCGAAGCACGTCACAAATGGCTGTGACATCAAGCCATCCATTGCCATCAGCCCTCCTAAGCTCACCCTCACCGGCAACATGGATAACCTCTACACACTGGTTATGACTGATCCTGACGCACCTAGCCCCAGTGAACCAAGCATGCGCGAGTGGATACATTGGATCTTAGTTGACATACCTGGAGGAACAAACCCATTTCGCGGAAAAGAGATTGTTTCATATGTGGGACCAAGACCACCTATTGGAATACATCGCTATATCTTTGTGTTGTTTCAACAGAAAGGACCTTTAGGTCTTGTGGAGCAACCACCAACTCGAGCAAGCTTCAACACTCGTTATTTTGCCAGGCAATTGGACTTGGGACTTCCAGTGGCCACTGTCTACTTCAACTCTCAAAAAGAACCTGCTGTTAAGAGGCGC

>GmTFLa.Glyma03g35250.Glyma.03G194700_Gm_TFL1b1 ATGGCAAGAATGCCTTTAGAGCCTCTAATAGTGGGGAGAGTCATAGGAGAAGTTCTTGATTCTTTTACCACAAGCACAAAAATGATTGTGAGTTACAACAAGAATCAAGTCTACAATGGCCATGAACTCTTCCCTTCCACTGTCAACACCAAGCCCAAGGTTGAGATTGAGGGTGGTGATATGAGGTCCTTCTTTACACTGATCATGACTGACCCTGATGTTCCTGGCCCTAGTGACCCTTATCTGAGAGAGCACTTGCACTGGATAGTGACAGATATTCCAGGCACAACAGATGCCACATTTGGGAAAGAGTTGGTGAGCTATGAGATCCCAAAGCCTAATATTGGGATCCATAGGTTTGTGTTTGTCCTGTTCAAGCAAAAGCGTAGACAGTGTGTTACTCCACCCACTTCAAGGGACCACTTCAACACACGCAAATTCGCAGCAGAGAACGACCTTGCCCTCCCTGTGGCTGCTGTCTACTTCAATGCACAGAGGGAAACGGCTGCAAGAAGACGC

>GmTFLb.Glyma10g08340.Glyma.10G071400_Gm_TFL1c1 ATGGCAAGGATGTCGACAGATCCTCTAATTATTGGGAGAGTCATAGGAGATGTTCTTGGCTCTTTCACCCCAACCATAAAAATGACCGTAACTTACAATAAGAAGCAAGTCTACAATGGGTACGAGTTCTTCCCTTCCACAATTACCACAAGGCCAAGGGTTGAGATTGGTGGAGGAGATATGAGGTCCTTCTATACACTGATTATGACAGACCCGGATGTCCCTGGCCCTAGTGATCCTTACCTGAGAGAGCATTTGCACTGGATGGTGACAGACATTCCAGGCACAACAAATGCCTCATTTGGGAAAGTGTTGGTGAGCTATGAGATGCCAAACCCTAACATTGGGATACACAGGTATGTGTTTGTCCTGTTGAAGCAAAAACGTAGGCAGTGTGTAACTCGTCCACCTTCTTCAAGGGATCACTTCAACACTCGCAAATTCTCAGCTGAGAATGACCTTGGCCTCCCTGTTGCTGCTGTCTACTTCAATGCACAGAGGGAAACTGCTGCAAGAAGACGC

>GmTFLc_Glyma13g22030.Glyma.11G209500_Gm_TFL1c2 ATGGCAAAGATGTGGACAGATCCTCTATTTATTGGGAGAGTTATAGGAGATGTTCTTGACTCTTTCACCCCAACCATAAAAATGACCGTAACTTACAAGAAGCAAGTCTACAATGGGCACGAGTTCTTCCCTTCCACAATTACCACAAGGCCGAAGGTTGAGATTGGTGGAGGAGATATGAGGTCATTCTATACACTGATTATGACTGACCCGGATGTCCCTGGTCCTAGTGATCCTTACCTGAGAGAGCATTTGCACTGGATGGTGACAGACATTCCAGGCACAACAAATGCCTCATTTGGGAATGTGTTGGTGAGCTATGAGATGCCAAAGCCTAACATAGGGATACACAGGTATGTGTTTGTCCTGTTTAAGCAAAAACGTAGGCAGTGTGTAACTCGTCCACCTTCTTCAAGGGATCACTTCAGCACTCGCAAATTCTCAGCTGAGAATGACCTTGGCCTCCCTGTCGCTTCTGTCTACTTCAATGCACAGAGAGAAACTGCTGCAAGAAGACGC

>GmTFLd.Glyma13g39360.Glyma.13G317100_Gm_TFL1a ATGGCGGCGAAGGAACTATATCCTCTTGTTATTGGGAGGGTGATCGGAGATGTAGTGGATCATTTCACTCCAACTGTGAAAATCACTGTCTCCTACAACAATAAGCAGGTCTATAATGGTCATGAGTTTTTCCTTTCCTCAGTAACCACTAAGCCTCAGGTTCAGATTCATGGAGGCGATATGAGATCGTTCTTCACTCTGGTCATGACAGATCCAGACGTTCCTGGCCCTAGTGATCCATATCTGAGGGAACACTTACACTGGATGGTCACAGATATCCCAGGCACAACGGACGCCACATTTGGAAATGAGGTGGTGGAGTACGAAATTCTAAGGCCAAACATAGGGATACATAGGTTTGTGTTTCTGGTTTTCAAGCAGAAGCGAAGGGGGAAGGAGAATGAGCTGGGGCCTCTTGTGGCTGCTGTGTTTTTCAATGCTCAAAGGGAAACCGCTGCCAGAAGACAT

>GmTFLe.Glyma19g37890.Glyma.19G194300_Gm_TFL1b2 ATGGCAAAAATGCCTTTAGAGCCTCTAATAGTGGGGAGAGTCATAGGAGAAGTTCTTGATTCTTTCACCACAAGCACAAAAATGACTGTGAGTTACAACAAGAAGCAAGTTTACAATGGCCATGAGCTCTTCCCTTCCACTGTCAACACCAAGCCCAAGGTTGAGATTGAGGGTGGTGATATGAGGTCCTTCTTTACACTGATCATGACTGACCCTGATGTTCCTGGCCCTAGTGACCCTTATCTGAGAGAGCACTTGCACTGGATAGTGACAGATATTCCAGGCACAACAGATGCCACATTTGGGAAAGAGTTGGTGAGCTATGAGGTCCCAAAGCCTAATATTGGAATCCATAGGTTTGTGTTTGTCCTGTTCAAGCAAAAGCGTAGACAGTGTGTTACTCCACCCACTTCAAGGGACCACTTCAACACACGCAAATTCGCAGCAGAGAACGACCTTGGCCTCCCTGTGGCTGCTGTCTACTTCAATGCACAGAGGGAAACGGCTGCAAGAAGACGC

>La_133n08_LanFTc1_La_FTc1 ATGGCAAGGGAAGGAAACCCTTTAGTTCTTGGGGGTGTAATAGGGGATGTCCTAAACCCCTTTACAACCTTAGTTTCTCTTAGGGTTTGTTTCAGTAACAGAGAAATTAGCAATGGTTATGAACTTAGGCCATCTCATGTTGTTAATCGTCCAAGAGTTTCTGTTGGTGGTGATGATCTCAGAACCTTCTACACACTGGTTATGGTGGATGCAGATGCACCTAGCCCCAGTAACCCTTTCTTGAGGGAGTACTTGCACTGGATGGTTACAGATATCCCAGCTACCACAAGTGCAGTGTTTGGAAGAGATGTCATGTTTTATGAGAGCCCACAGCCTTCAGAAGGAATTCATCGACTTGTGTTTGTATTATTCAAGCAATTGGGCAGAGACACTGTCTTTGCTCCAGAATGGCGTCAAAATTTTAACAGCAGAAGCTTTGCTGAAATTAATAATCTGATACCAGCTGCAGCAGTTTATTTCAACTGCCAAAGAGAGCGTGGTTGTGGTGGAAGAAGAACAGAG

>La_137o24_LanFTc2_La_FTc2 ATGGCACATGATGATGAAAACCCTCTTGTTCTTGGGGGTGTAATAGAAGATATCCTGTACTCTTTTACAAGCTCAGTTTCTCTTCTGGTTTTCATCAATAACAGAGAGATTAGCAATGGTTGTGAACTCAGGCCCTCTCACCTTGTTAAACGCCCAAGAGTTTCTGTTGGTGGTGAAGACCTCAGGACCTTTTACACACTGGTTCTGGTGGATGCAGATGCACCTAGCCCTAGTAATCCTTTCTTGAAGGAATACTTACACTGGATGGTGACTGATATTCCAGCTACCACAAGTGCAGTATTTGGGAAGGAGGTTATGTTTTATGAGAGGCCAGAACCTTCAGCAGGGATTCATCGCAATGTTTTGATATTATTCAAACAACTTGGCAGAGACACTGTTATCACTCCACAATGGCGTCAAAATTTTAAAAGCAGAAGCTTCGCTGAAAGTAACAATCTCGTTCCTGTTGCAGCTGCTTATTTCAACTGCCAAAGAGAGCATGGTTGTGGTGGAAGAAGATCAGAA

>La_scaffold11929_La_MFTc ATGATATGTAGCAAACATGTGTCTAATGGTTATGATATCAAACCATCTTTAGCAAGTGAAAGACCCCATGTTAAAATTGTTGGTGCCTCTAATGCTGCCAACCTCTACACTTTGGTAATGGTGGATCCTGATGCTCCATCTCCAAGTGAACCAATTCATAGAGAGTGGCTCCATTGGATTGTTGTAGACATTCCAGAGGGATCAGATGCCACTCAAGGGAGGGAATTGGTGAAGTATATGGGGCCATGCCCAGCAGTGGGAATTCATCGCTACGTCTTTGTAGCATTTAAGCAAACAGGTGGTGGACTAGTTAAAGTTGAGGCACCATTACAAGGTTGTCAAAATTTTAACACTCGCCAATTTGCTTCACTAAACAACCTTGGTCTTCCAGTTGCTGCTATTTACTTCAATTCACACAAAGAACCTAAAAACACAAAATTTATT

>La_scaffold4378_La_TFL1b1 ATGGCAAGAATGTCATTAGAGCCTCTAGTTCTTGGAAGAGTCATAGGAGAAGTTCTTGAATCTTTTACAACCACCATGGAAATGACTGTTACTTATAACAACAAGCTAGTCTTCAATGGCCATGAGCTATTACCTTCCACTGTCACAAACAAACCTAGGGTTGAGATTGGTGGTGCTGATTTCAGGTCCTTCTTTACACTGGTTATGACAGACCCTGATGTTCCTGGCCCTAGTGATCCTTATCTGAGGGAGCACTTGCACTGGATTGTGACTGACATTCCAGGCACAACAGATGCCACATTTGGGAAAGACTTGGTGAGTTATGAGATTCCAAATCCAAATATTGGGATTCATAGGTTTGTGTTTGTTTTGTTCAAGCAAAAACGTAGGCAATGTGTTACTCAACCATCTTCAAGGGAACACTTCAATACTAAGAATTTTGCACTAGACAATGACCTAGGGCTCCCTGTTGCTGCTGTCTACTTCAATGCACAGAGAGAAACTGCTGCAAGAAGACACTTATTAGCTGGAACCTATAAG

>La_scaffold49815_La_BFT ATGTCTAGACTCATGGAACCACTTGCTGTGGGAAGAGTAATAGGAGAGGTGATAGACATGTTCACCCCAAGTGTGAGAATGAATGTGATATATTCCACAAAGCAAGTTGCTAATGGTCATGAACTAATGCCTTCTATTGTTATGGCCAAACCAAGAGTTGAGATTGGTGGTGATGATTTGAGAGCTGCTTATACCTTGATTATGACAGACCCAGATGCACCAAGTCCCAGTGATCCATATTTAAGGGAATATCTCCACTGGATGGTTGTAGATATTCCTGGTACCACAGATGCTTCTTTTGGTAATGAGATTATGGGGTATGAGAGTCCAAAACCTGTAATAGGGATCCACAGATACGTACTAATCTTGTTCAAACAAAGAGGAAGAAAAACAGTGAGACCCCCAACTTCTAGACATAATTTCAACACAAGGAGGTTCTCAGAAGAAAATTGTCTTGGTCTACCTGTTGCTGTTGTTTACTTCAATGCCCAAAGAGAAACTGCTGCAAGAAGAAGA

>La_scaffold53720_La_TFL1b3 ATGGCAAGAATATCATCAGAACCTCTAATTGTTGGGAGAGTCATAGGAGATGTTCTTGATTCTTTTACCACTAGCATGAAAATGACTGTGAGTTATAACAACAAGCAAGTTTTCAATGGGCATGAGCTCTTCCCTTCCACTGTTACCAACAAGCCTAGGGTTGAAATTGATGGTGCTGATTTGAGGTCCTTCTTTACACTGGTCATGACAGACCCTGATGTTCCTGGCCCTAGTGATCCTTATATGAGAGAGCACCTGCACTGGATTGTGACTGACATTCCAGGCACAACAGATGCCACATTTGGGAAAGAGTTGGTGAACTATGAGATTCCAAAGCCAAATATTGGGATCCATAGGTTTGTGTTTGTACTGTTCAAGCAAAAACGTAGGCAATGTGTTACTCAACCTTCTTCAAGGGATCACTTCAATACTATGAATTTTGCATCAGAAAATGACCTTGGTCTTCCTGTTGCTGCTGTCTACTTCAATGCACAGAGGGAGACCGCTGCAAGAAGGCGC

>La_scaffold66207_La_TFL1a ATGGCAGTGAATATGAAATCAGATCCCCTTGCAATTGGGAGGGTGATTGGAGATGTGGTTGATTATTTCACCCCAAATGTGAAAATGAGTGTCACATACAACCACAATAAGCAAGTCTACAATGGTTTTGAGTTTTTTCCTTCCTCAGTTTCCACTAAGCCTAAGGTTCAGGTTCATGGAGGTGACATGAGATCCTTCTTCACTCTGGTGATGACAGATCCAGATGTCCCTGGCCCTAGTGATCCATATCTGAGGGAACACTTACACTGGATAGTGACAGATATCCCAGGCACAACGGATGCCACATTCGGAAAAGAGGTTGTGAAATATGAAATGCCAAGACCAAACATAGGGATACATAGGTTTGTGTTCCTTCTATTCAAGCAGAAGGGCAGGCAAACAGTAAATAAAATACCAAGTTCAATGGACCTCTTTAATGCAAGGAACTTTGCACATGTCAATGACCTTGGTCCTCCTGTGGCTGCTGTTTTTTTCAATGCTCAAAGGGAAACCGCTGCCAGAAACCGT

>La_scaffold66814_La_FTa1a ATGGTGAGTCGTAGTAGAAACCCTCTTGTTGTTGGACGTGTAATTGGGGATGTTTTAGATCCCTTTGAAAGTTCCATTACTATGAGAGTGAGTTACAACAATAGAGAAGTTAGCAATGGTTGTGAGTTCAAACCTTCACATGTTGTTAATCAACCAAGGGTGAGTGTTGGTGGAGATGATCTTAGGAACTTCTATACTCTGGTTGTGGTGGACCCAGATGCACCTAGTCCAAGTGACCCCAATTTGAGGGAATACCTTCACTGGTTGGTGACTGATATTCCAGCAACTACTGGGCCTAGTTTCGGTCATGAGGTTGTAAGTTATGAAAATCCAAGACCCTTGATGGGAATTCATCGATGGCGCCAAAATTTCAACACAAGAGAATTTGCTGAACTTTACAATCTTGGATCACCAGTTTCAGCTGTTTATTTTAACATTCAGAGAGAATCTGGTTCTGGTGGAAGA

>La_scaffold84569_La_MFTa ATGGCCACTTCGGTTGATCCTCTCATAGTTGGTCGTGTCATTGGTGATGTGGTTGACATGTTTGTTCCTTCTGTGAACATGTCTGTGTACTTTGGTACTAAGCATGTTACTAATGGTTGTGACATCAAACCTTCCATTGCTCTTGATCCACCAAGAGTTACACTTACTGGTAACATGGACAACCTCTACACCCTTGTTATGACAGACCCAGATGCACCAAGCCCCAGTGAACCAAGTCATCGCGAGTGGATACATTGGATTGTGACTGACATACCTGGAGGAACAAACCCACACCGCGGAAAGGAGATCCTTCCTTATGTTGGACCAAGGCCACCAGTGGGGATACACCGCTACATCCTGGTGCTTTTTCAGCAGAAGGGGCCACTGGGTCTGGTGGAGCAGCCACCGTCTCGTGCAAGCTTCAACACTCGCTACTTTGCTAGACAATTGAACTTGGGACTCCCTGTTGCAACTGTCTACTTCAACTCTCAGAAGGAACCTGCATCTAGGAGGCGCACA

>La_scaffold88544_La_TFL1b2 ATGGCAAGAATGTCATTAGAACCTTTAATTGTAGGGAGAGTCATAGGAGATGTTCTTGATTCTTTTACCAGTACCATGAAAATGACTGTGAGTTACAATAACAAGCAAGTCTTCAATGGCCATGAACTCTTCCCTTCAACTATCACCAACAAGCCAAGGGTTGAGATTGATGGTGGTGATTTGAGGTCCTTCTTTACACTGGTTATGACAGACCCTGATGTTCCTGGCCCTAGTGATCCTTATCTGAGAGAGCATTTGCACTGGATGGTGACTGATATTCCAGGTACAACAGATGCCACATTTGGGAAAGACTTGGTGAGCTATGAGATTCCAAAGCCAAATATTGGGATCCATAGGTTTGTGTTTGTCTTGTTCAAGCAAAAAAGGAGGCAATGTGTTACTCAACCATCTTCAAGGGATCACTTCAATACTAAGAATTTTGCAGCAGAAAATGACCTTGGTCTTCCTGTTGCTGCTGTTTACTTCAATGCACAGAGAGAAACTGCTGCAAGAAGGCGC

>La_scaffold99417_La_FTa1b ATGAGTAGTAGTCGTAGTAGGAACCCTCTAGTTGTAGGACGTGTAATTGGTGATGTATTGGACCCATTTGAAAGTTCTATTCCTATGAGAATCACTTACAACAATAGAGATGTTAGCAATGGCTCTGAGTTCAAACCTTCCCATGTTGTTAATCAACCAAGAGTGACTATTGGTGGCGATGACCTCAGGAACTTGTACACTCTGGTTGTGGTGAACCCGGATGCACCTAGTCCAAGTGACCCTAATTTTAGGGAATACCTTCACTGGTTGGTGACTAATATTCCAGCAACTACTGGCCCTACTTTCGGTAATGAGGTTGTAAATTATGAAAGTCCACGACCCTCGATGGGAATTCATCGTATAGTATTTGTGTTATTCCATCAACTTGGTAGGGAAATAGTGTATGCTCCAGGATGGCGCCAAAATTTCATTACTAGAGAATTTGCTGAACTATACAATCTTGGTTCACCAGTTGCTGCTGTTTATTTCAACATTCAGAGGGAATCTGGCTCTGGTGGAAGAAGGTTATGT

>La_UWAscaffold13887_La_MFTb ATGGCCACCTCCGTTGATCCCCTCATAATTGGTCGCGTCATAGGTGACGTAGTTGACATGTTTGTTCCTTCTGTGAACATGTCTGTGTACTTTGGTACCAAGCATGTCACTAATGGTTGTCACATCAAACCTTCCATGGCTGTCGACCCACCCAGAATTACACTCACTGGTAACATTGATAACCTCTACACTCTGGTCATGACGGACCCAGATGCACCAAGCCCGAGTGAACCAATTCACCGCGAGTTGATACATTGTGCACACAGGCCCGAATATCTCTTTTGCTATATAGAGCAGAACCATGATTTTAAGCTGTGGCAAATTGTTGGCTGCAATATTGTAGTTGGCGGAAATGAGATCCTTCCATATGGTGGACCAAGGCCACCATTGGGGATACACCGCTACATCCTGGTGCTTTTTGAGCAGAAAGGACCACTGGATCTGGTGGAGCAGCCACCCTCTCGTGCAAGCTTCAACACTCGTTACTTTGCTAGACAATTGAACTTGGGACTCCCTGTTGCAACTGTCTACTTCAACTCTCAGAAGGAACCTGCATATAGGAGGCGCACA

>Lj01_39575559_39577375_chr1.CM0017.410.r2.a_Lj_FTb2a ATGCCTGGTAAAACAATAAACCCTCTTGTTGTTGGACGTGTGATAGGAGATGTTTTGGACCCCTTCACAAGCTCTATCTCTATGAGGGTTCTTTATAACAATAACAAAGAAGTCATCAACAGTGGTGAGTTCAAACCTTCCCAAATAATTAACCAACCAAGAGTTGATATTGGTGGAGATGACCTCAGGACCCTTTACACTCTGATCATGGTGGATCCTGATGCACCAAGCCCAAGTGACCCAAATATGAGGGAATATCTACACTGGTTGGTGACCAATATTCCAGCAACTACTGGATCAGGGTTTGGACAAGAGATTGTGAGCTACGAAAATCCACGACCAACATCAGGGATTCATCGTTTTGTTTTTGCCTTGTTTCACCAGCCTTGTAGGCAAACCATACTTGCTCCTGGGTGGCGCCAAAACTTCATTACTAGAGATTTTGCAGAGGTTTATAATCTTGGATTACCAGTTGCAGCTATGTATTTCCATTGTCAACGAGAAAGTGGTTCCGGCGGAAGGAGGATCATG

>Lj01_39616127_39617933_chr1.CM0017.450.r2.a_Lj_FTb1a ATGCCTAGATCAACTGATCCTCTTGTTGTTGGAGGTGTAATAGGAGAAGTCTTAGATCCTTTTACAAGTTCTATCTCTCTAAGGGTTGTCTATAACAATACCTCACAAGTTATCAACTGTTGTGAGCTTAGGCCTTCCCAAATCGCCAGCCAACCAAGAGTTGAAGTTGGTGGTCATGACCTCAGGACCTTTTACACTCTGATCATGGTGGATCCTGATTCACCTAGCCCAGGTGATCCACATCAAAGGGAATATTTGCATTGGTTAGTAACCAATATTCCAGCAACTACAGGAGCAAACTTTGGAGAAGAGATTGTATCTTATGAAAGTCCACGACCAACAGCAGGGATTCATCGTATAGTTTTTGTGCTGTTTCGTCAGTTGGGGGGTAGACAAACACTACATGCACCTGGGTGGCGCCAAAATTTTCACACTAGAGACTTTGCCGAGGTTTACAATCTTGGATCACCTGTCGCTGCAACATACTTCAACTGTCAACGAGATGCTAGGAGTACTGCAAGGAACAGCAGGAGA

>Lj01_49026887_49028547_chr1.CM0104.1670.r2.m_Lj_FTa2a ATGCCAAATAGCAGAAATCCTCTTGTTGTTGGGCGGGTAATAGGTGATGTATTAGACCCCTTTGAAAGCTCAATTCCTCTAAGAGTCACATATGGTAGTAGAGATGTCACCAATGGCTGTGAGTTTAAACCTTCCCAAGTTCCCAATCAACCCAGAGTGTCGGTCGGCGGAGATGACCTCAGAACCCTCTACACTTTGGTCTTAGTTGATCCTGATGCACCTAGCCCAAGTAACCCCAGCTTCAGGGAGTACCTTCACTGGTTGGTGACTGATATTCCAGCGACTACAGGGGCAAGTTTCGGTAATGAGATTGTAGCTTATCAGAGCCCACGACCCACCATGGGGATTCATCGTTTCGTGTTCGTGTTGTTCCGCCAACAGTGTAGACAAAGAGTATATGCTCCGGGATGGCGACAAAATTTTAATACGAGAGAATTTGCTGAACTTTACAATCTTGGGTTGCCAGTTGCTGCTGTCTTCTTTAACTGTCAGAGGGAGACTGGTTCTGGTGGAAGAACATTT

>Lj01_60197653_60196409_chr1.CM0010.70.r2.d_Lj_TFL1b ATGAAAGTGACTGGGCGTTGCAATACCACGCAAGGGCTGGATGGCCCTGAGTTCTTCCCTTCCACTATTAACACCAAGCCCAAGGTTGATATTGGTGGTGGTGACATGAGGTCCTTCTTTACAGTGATCATGACAGACCCAGATGTTCCTGGACCTAGTGATCCTTATCTCAAAGAGCACCTGCACTGGATGGTGACTGATATTCCCGGAACAACAGATGCCACATTTGGAAAAGAGTTGGTGAGCTATGAGATTCCAAAGCCTAACATTGGGATCCATAGGTTTGTGTTTGTCCTGTTCAAGCAAAAACGTAGGCAATGTGTGAGTCCACCATCTTCAAGGGATCACTTCAACACTCGCAGTTTTGCAGCACAGAATGACCTTGCTCTCCCTGTTGCTGCTGTCTACTTCAATGCTCAGAGGGAAACTGCTGCAAGAAGACGC

>Lj04_42914991_42913216_chr4.CM0042.2080.r2.m_Lj_MFT ATGGCTGCATCTGTTGATCCTCTGGTTGTGGGAAGAGTGATCGGAGATGTGGTTGACATGTTTGTTCCATCAGTGAACATGTCTGTTTACTTTGGTTCAAAGCATGTCACCAACGGCTGTGACATCAAGCCTTCCATTTGTATCAGCCCTCCAAAGGTCACTCTCACCGGCAACATGGACAACCTCTACACCCTGGTTATGACTGACCCTGATGCACCAAGCCCCAGTGAGCCCAGTCTTCGCGAGTGGATACACTGGATCGTTGTTGACATTCCTGGGGGAACAAACCCAAACCGAGGAAAAGAGGTGCTACCTTATGTTGGGCCAAGACCCCCAGTGGGCATTCACCGCTTCATATTTGTGTTGTTCAAGCAGAAGAGGCCACTAGGGCTTGTTGAGCAGCCACCAACCCGTGCAAGCTTCAACACCCGCTACTTCGCTCAGCAACTTGAGTTGGGCCTCCCTGTGGCGACTGTTTACTTCAACTCACAGAAAGAGCCTGCAACTAAGAAGCGT

>Lj05_12575624_12573824_chr5.CM0657.210.r2.m_Lj_TFL1c ATGGCTAGGGTGTTGTCAGATCCTCTAAAAGTTGGGACGGTGATTGGAGATGTTCTTGATCCTTTCATCCCAACCATAAAAATGTCTGTATTTTACAATAAGAAAGAAATCTTCAACGGATATGAGCTCTTCCCTTCCACAGTGACCACCAGGCCCAGGATTGAGATTGGAGGTGACATGAGGTCCCTCTTTACACTGATCATGATAGACCCAGATGTTCCTGGCCCTAGTGATCCTTATCTTAGAGAGCACTTGCACTGGATGGTGACTGACATTCCAGGCTCAACAGATGCCACATTTGGAAAGGAGTTGACAAGCTATGAGATCCCAATGCCTATTATAGGGATCCACAGGTATGTGTTTGTCCTTTTCAAGCAAAATCGCAAGAACAAGGGTGTTGTTAGTGTTACCCCACCATCAGCTTCAACTTCAAGTAGGGATCACTTCAACACTCGCGATTTCTCTGCTGAGAATGATCTTGGCCTCCCTGTTGCTGCTGTCTACTTCAATGCACGGAAGGAAACTGCTCCAAAGAGGCGC

>Mt01_26206613_26203089_Medtr1g060190.1_Mt_TFL1c ATGGGAAGTATCACTTCAGATCCACTAATTCTTGGGAGAGTGATAGGAGATGTTATTGACTATTTTACCCCAACCACAAAAATGACTGTGACTTACAACAACAAAGAAATCTTCAATGGATATGAACCCTTTCCTTCTTCAGTTACCACCAAGCCAAGGATTGAGATTGGAGGAGTGGACATGAGGTCCCTCTTTACACTGATCATGATAGATCCAGATGTTCCTGGCCCAAGTGATCCTTATATGAAAGAACACTTACACTGGATGGTGACAGACATTCCAGGGACCACAGATTCCACATTTGGCAAAGAATTGACAAGCTATGAAAAACCAAAGCCTAATATAGGAATCCACAGATATGTGTTTGTCCTATTCAAGCAAGAAAAGGGGAAGAAGCACTCAATTGTTGCTCCTTTTTCAAGGGATCACTTTAACACACGTGCCTTTTCTGCTCAAAATGACCTTGGTGTCCCTGTTGCTGCTGCATATTTCAATGCAAGAAGGGCAACGGCTCCTAGAAGGCGTGCTAGC

>Mt02_36279047_36277180_Medtr2g086270.1_Mt_TFL1a ATGAGTATTGTAACAGATCCTCTTGCTATTGGAAGAGTGATTGGAGATGTAGTTGATTATTTCACTTCAACTATGAAAATGTCTGTCACTTACAACACTAAGCAAGTTTACAATGGTCATGAGTTTTTTCCTTCCTCAGTTACCACTAAACCTAAAGTTCAGATTCATGGTGGTGATATGAGATCTTTCTTCACTCTGGTCATGACAGATCCAGATGTGCCTGGCCCTAGTGATCCGTACCTGAAGGAACACTTACACTGGATAGTCACAGATATACCAGGCACAACTGATGCTACATTTGGGAAAGAAGTGATGAAATATGAAATGCCACGACCTAACATAGGAATCCATAGGTTTGTGTTCCTTCTTTACAAGCAGAAGCGCAGACAGACAGTGATGAAAATACCAACATCAAGAGATCTCTTTAACACAAAGAAATTTGCACAAGACAATGACCTCGGGCCTCCTGTGGCTGCTGTTTTTTTCAATGCACAAAGAGAAACAGCTGCCAGAAGACGT

>Mt06_10371673_10369992_Medtr6g033040.1_Mt_FTa1a ATGAGTGGTAGTAGTAGAGACCCTCTTGTTGTTGGAGGTGTAATTGGTGATGTATTGGTCCCCTTTCAAAGTTCTATTCCTATAAGAGTTTCCTACAATGGTAAAGAACTTAACAATGGCTGTGAATTCAAACCTTCTCAAGTTGTCAATCAACCAAGAGTTAGTGTTGGTGGAGATGATCTTAGAAACTTCTATACTCTGATCATGGTGGATCCAGATGCACCTAGCCCTAGTAACCCAAATTTGAGGGAGTATCTTCATTGGTTGGTGACTGATATTCCTGCAACTACTGGACCTACTTTTGGTCATGAAGTGGTCCCTTATGAGAGTCCACGACCTTCGATGGGAATTCATCGCATCGTCTTTGTGATTTTTCGTCAACTTGGTAGAGAGACGGTTTATGCTCCAGGTTGGCGACAGAATTTCAACACTAGAGAATTTGCAGAACTTTACAATTTGGGATTGCCAGTTGCTGCTGCTTATTTTAACATTCAAAGGGAACATGGCTCTGGTGGAAGAAGGTTA

>Mt07_32843587_32845288_Medtr7g084970.1_Mt_FTa2a ATGGCTGGTAGCAGTAGGAATCCACTAGCTGTAGGGCGTGTAATAGGGGATGTAATAGACTCATTTGAAAATTCCATTCCTCTTCGAGTGACCTATGGTAATAGGGATGTGAATAATGGTTGTGAGCTCAAACCTTCTCAAATTGGAAATCAACCGAGAGTGAGTGTTGGTGGAAACGATCTCAGAAACCTCTACACCCTAGTTATGGTGGATCCTGATTCACCTAGCCCAAGTAACCCCACTTTTAAGGAGTACCTTCACTGGTTGGTGACTGATATTCCAGGAACCACTGAAGTCACTTTCGGCAATGAGGTTGTAAATTATGAAAGGCCACGACCCACTTCAGGGATCCATCGTTTCGTGTTTGTCTTATTCCGTCAACAGTGTAGACAAAGGGTTTATGCTCCAGGATGGCGACAAAATTTCAACACAAGAGAATTTGCTGAACTCTACAATCTTGGATCACCTGTTGCTGCTGTCTTCTTCAATTGTCAGAGGGAGAGTGGCTCTGGAGGAAGAACCTTTAGA

>Mt07_32863573_32865790_Medtr7g085020.1_Mt_FTa2b ATGGCAAGTGGTAGCAGACCGAATCCTCTTGCTGTTGGGCGTGTAATAGGGGATGTATTAGACCCCTTTGAAAGTACTATTCCTCTCTTAGTCACCTATGGTAATAGGACTGTTACCAATGGTGGTGAGCTTAAACCTTCCCAAGTTGCTAATCAACCCCAAGTGATTATTGGCGTAAATGACCCAACAGCCCTCTACACCCTGGTTTTGGTAGATCCAGATGCTCCTAGTCCCAGTTACCCCAGTTTTAGGGAGTACCTTCATTGGATGGTGACTGATATTCCAGCAACTAATGCGGCTAGTTTTGGTAATGAGGTTGTAAGTTATGAAAAGCCACGACCCAATTTAGGGATTCATCGTTTCGTGTTTGTATTATTGCATCAACAGTGTAGACAAAGAGTCTATGCTCCGGGATGGCGACAAAATTTCAATACAAGAGAATTCATTGAATTTTACAATCTTGGATCGCCGGTTGCTGCTGTCTTCTTCAATTGTCAAAGGGAAACTGGTTCTGGGGGAAGAACCTTTAGA

>Mt07_32874924_32877059_Medtr7g085040.1_Mt_FTc ATGCCACAAAATTTGGTCGACCCTCTTGGTGTTATAGGAGATGTTTTGAGCCCTTTTACAAATTCTGTATCTTTGAGTGCTCTCATCAATAACAGAGAGATTAGCAATGGATGTATAATGAAACCCTCTCAACTAGTTAATCGTCCAAGGGTTAATGTTGGTGGTGATGATCTCAGGACTTTCTACACAATGGTTATGGTGGACGCAGATGCACCTAGCCCTAGTAACCCTTTTTTGAAGGAATACTTGCATTGGATGGTGACGGATATTCCAGCAACAACAAGTGCAAGCTTTGGGAAAGAGGTAGTGTTTTATGAGAGCCCGAAACCTTCAGCAGGGATTCATCGATTTGTGATTGCATTATTCAAGCAACTTGGCAGAGACACTGTTTTCGCCCCAGATTGGCGTCATAATTTCAACACTACGAACTTTGCTGAAATTAACAATTTGGTAATTGTTGCTTCAGTTTATTTCAACTGCCAACGAGAGCGTGGTTGCGGCGGAAGGAGATGT

>Mt07_42325631_42324586_Medtr7g104460.1_Mt_TFL1b ATGGCAAGAATGTCTCAAGAACCACTCATTGTTGGAAGAGTGATAGGAGAAGTTCTTGATTCCTTTACCACAAGCATGAAAATGACTGTGAGTTACAACAAGAAGCAAGTCTTCAATGGCCATGAGTTTTTCCCTTCCACTATCAACACCAAACCCAAAGTTGAGATTGATGGTGGTGACATGAGGTCCTTCTATACACTGGTGATGACAGACCCTGATGTTCCTGGCCCTAGTGATCCTTATCTAAGAGAACACTTGCACTGGATTGTGACAGATATTCCTGGAACAACAGATGCTACATTTGGGAAAGAAGTAGTGAGCTATGAGATACCAAAGCCAAATATAGGTATACACAGGTTTGTGTTTGTTCTATTCAAACAAAAGAATAGAGAATCAGTGACAGCATCACCATCTTCAAGGGATTACTTCAACACTCGCAATTTTGCTTCACAGAATGATCTTGGTCTCCCTGTTGCTGCTGTTTACTTCAATGCTCAGAGAGAAACCGCTGCAAGAAGACGC

>Mt07_776568_774513_Medtr7g006630.1_Mt_FTb2a ATGAACCCTCTTGTGGTTTGTGGTGTAATTGGAGATGTTTTGGATCCCTTTACAAATTCAGTGTCTTTGAGGGTCGTTTATGAAAATAACAAAGAAGTCAGCAACAGTGGCGAGCTGAAACCCTCCCAAATAGTCAATCCACCAAGAGTTCAAGTTGGTGGAAATGACCTCAGGACTCTATACACTCTGGTGATGGTGGACCCTGATGGACCAAGCCCTAGTAACCCTAATATGAGGGAATACCTGCATTGGATGGTAACCAATATTCCAGCGACTACAGGGACAACTTTCGGACAAGAGATAGTGAGCTATGAAAATCCAAGACCAACATCAGGGATTCATCGTGTGATATTTGTGTTGTTTAGGCAACCTTGTAGGCACACAGTATTAGCTCCTGGATGGAGACAAAATTTCATTACAAGAGATTTTGCTGAATTTTACAATCTTGGATTACCTGTTGCTGCTCTCTATTTCAATTGTCAACGAGAAAATGGTTCTGGTGGAAGGAGGTTGATCATC

>Mt07_817865_814446_Medtr7g006690.1_Mt_FTb2b ATGCGTATTAAATCAACAAATCCTCTTGTTGTTGGTGGTGTAATTGGAGAGGTTTTGGACCCCTTTACAAGTTCAGTGTCTTTGAGAGTCGTTTATGACAATAACAAAGAAGTCATCAACAGTGGTGAGCTCAAACCCTCCCAAATAATCAACTCACCAAGAGTTCAAGTTGGTGGAAATGACCTCAGGACCCTGTATACTCTGGTGATGGTGAATCCTGATGCACCAAGCCCTAGTGACCCGAATATGAGGGAATACCTGTATTGGATGGTAACCAATATTCCAGCGACTACAGGGACAACTTTCGGACAAGAGATAGTGAGTTATGAAAGTCCAAGACCAGCATCAGGGATTCATCGTGTGATATTTGTGTTGTTTAGGCAACCTTGTAGGCACACAGTATTAGCTCCTGGATGGAGACAAAATTTCATTACAAGAGATTTTGCTGAATTTTACAATCTTGGATTACCTGTTGCTGCTCTCTATTTCAATTGTCAACGAGAAAATGGTTCTGGTGGAAGGAGGATGGTCATT

>Mt08_45110632_45108361_Medtr8g106840.1_Mt_MFT ATGGCTGCCTCGGTTGATCCTTTGGTGGTTGGTCGTGTGATTGGTGATGTTGTTGACATGTTCATTCCATCTGTTGGCATGTCTGTTTACTTTGGTCCTAAACATGTCACAAATGGTTGTGACATAAAGCCATCCATGGCTATCAACCCACCCAAGGTCACTCTCACTGGAAACATGGATAACCTCTACACTCTGGTTATGACTGATCCAGATGCACCAAGCCCCAGTGAGCCAAGCATGCGTGAACTGATACACTGGATTGTGGTTGACATTCCTGGAGGAACAAATCCAAAGCGAGGAAAGGAGATTCTTCCATATATAGGACCAAAACCACCGGTTGGTATCCACCGATACATATTGGTTTTGTTTGAGCAGAAGGGACCAATTGGAATGGTTGAGCAGCCAACCAGTAGAGTCAGTTTCAACACTCGTTATTTTGCAAGTCAAATGAACCTTGGTCTTCCTGTAGCTACAGTCTACTTTAACTCTCAGAAGGAGCCTCAGGCTAAGAGGCGT

>Mtscaffold0020_150907_152710_Medtr0020s0120.1_Mt_BFT ATGTCTAGGCCATTGGAACCACTTTCTGTGGGAAGAGTGATAGGAGAAGTTGTTGACATTTTCAATCCAAGTGTAAGAATGAATGTGACATATTCCACCAAGCAAGTTGCAAATGGACATGAGTTAATGCCTTCTATTGTTATGAACAAACCAAGGGTTGATATTGGAGGTGAAGACATGAGATCTGCTTATACTTTGATCATGACAGACCCAGATGCTCCTAGTCCTAGTGATCCACATTTAAGGGAACATCTCCACTGGATGGTTACAGATATTCCAGGTACCACAGATGTCTCTTTTGGAAATGAGATTGTGGAGTATGAGAATCCAAAGCCAGTGATAGGAATCCATAGATATGTGTTCATCTTGTTCAAGCAGAGAGGGAGACAAACAGTGAGATCCCCATCTTCTAGAGACAATTTCAACACAAGGAGATTCTCACAGGAGAATAACCTTGGTTTACCTGTTGCTGCAGTTTACTTCAATGCTCAAAGAGAAACTGCAGCAAGAAGAAGG

>Pv01_21439344_21438351_Phvul.001G097200.1_Pv_FTc ATGGCACGAGAGGATCCTCTTGCTATTGGGGGTGTGATAGGGGATGTTCTGAACCCTTTTACAAGCTCAGTTTCTCTCACAGTTTCCATCAACAACAGAGCCATTAGCAATGGCTATGAACTCAGGCCCTCTCTAGTTGTTAACCGCCCTAGAGTTACTGTAGGTGGTGATGACCTAAGGATCTTCTACACTCTGGTTATGGTAGATGCAGATGCACCTAGCCCTAGTAACCCCGTCTTGAGGGAATACATGCACTGGATGGTGACAGATATTCCAGCAACCACAAATGCAAGCTTTGGTAAGGAGGTTGTGGTTTATGAGAGTCCACAACCTTCAGCAGGAATTCATCGACTTGTGTTTGTATTGTTCCAGCAATTGGGCAGAGACACTGTCATCACCCCAGATTTGCGTCATAATTTCAATTCCAGAGACTTTGCTGAAAATAATAACCTCACACCTGTTGCAGCAGCTTACGTCAACTGCCAAAGAGAGCGTGGTTGCGGTGGAAGGAGATAT

>Pv01_21457835_21455436_Phvul.001G097300.1_Pv_FTa2a ATGCCTACTGGTAGTAGAAATCCTCTTGTGGTTGGGCGTGTTATAGGAGAAGTAGTAGACCCCTTTGAAACTTCTATTCCTTTCAGGGTCTCCTATGGCAATAGAGAGGTCAACAATGGTTGTGAGCTTAAACCTTCCCAAGTTGCAAACCAACCCAGAGTTAGTGTTGGTGGAGATGATCTCAGGATCTTCTATACTCTGTTGTTAGTAGATCCTGATTCTCCCAGCCCAAGTAATCCCAATTCTAGAGAGTACCTTCATTGGTTGGTGACTGATATTCCAGCAACTACTGGGGCTAGTTTCGGTAACGAGGTTGTTAGTTATGAGAGCCCACGACCAACGATGGGGATTCATCGTTTGGTGTTTGTGTTGTTCCGTCAACAGTATCGACAGAGGGTGTATGCTCCTGGATGGCGACAGAATTTCAACACAAGAGAATTTGCTGAACTTTACAATCTTGGATTACCAGTTGCTGCAGTCTTCTTCAACTGTCAGAGGGAAACTGGTTCTGGTGGTAGGACATTT

>Pv01_45563048_45561749_Phvul.001G189200.1_Pv_TFL1b ATGGCAAGAATGCCTTTAGAACCTCTTATAGTGGGGAGAGTCATAGGAGAGGTTCTTGACTCTTTTACCACAAGCATGAAAATGACTGTGAGTTATAACAAAAAGCAAGTCTACAATGGCCACGAGCTCTTCCCTTCATCTGTCAACACCAAACCCAAGGTTCAGATTGAGGGTGCTGATATGAGGTCCTTTTTCACACTGATCATGACAGACCCTGATGTTCCAGGCCCTAGTGACCCTTATCTCAGAGAACACTTGCACTGGATAGTGACAGATATTCCAGGCACAACAGATGCTACATTTGGGAAAGAGTTGGTGAGCTATGAGATCCCAAAGCCTAATATTGGGATCCATAGGTTTGTGTTTGTCCTGTTCAAGCAAAAGCGTAGGCAGTGTGTTACTCCACCAACTTCAAGGGATCACTTCAACACACGCAATTTCGCAGCACAGAACGACCTTGGCCTCCCAGTGGCTGCTGTCTACTTCAATGCACAGAGGGAAACAGCTGCAAGAAGACGC

>Pv02_48577594_48575762_Phvul.002G327700.1_Pv_MFT ATGGCTGCATCTGTTGACCCTCTTGTGGTGGGTCGAGTAATAGGTGATGTGGTCGACATGTTTGTCCCCTCAGTCAACATGTCTGTGTACTACGGTTCCAAACATGTCACAAGCGGATGTGACATTAAGCCATCCATTGCAGTCAACCCACCTAAGCTCACTCTAACTGGAAAGAAGGAAAACCTTTACACTCTGATTATGACTGATCCTGATGCACCAAGCCCCAGTGAACCAAACATGCGTGAATGGATCCACTGGGTGGTGGCGGATATACCTGGTTGCACAAATCCCTTTCGTGGAAAAGAAATCATCCCATACACTGGACCAAAGCCACCTGTGGGAATCCATCGTTACATCTTTGTGCTCTTTGAACAGAAAGCACCAATGGGTCCTGTGGAACAGCCAGAAAGTAGAGGGAACTTCAGCACTAGAACTTTTGCAAAGGACATGGATTTGGGTCTCCCTGTGGCCACAGTGTATTTCAACTCTCAGAAGGAACCTGCTTCTAGGAAGAGA

>Pv04_12306580_12310414_Phvul.004G074700.1_Pv_FTa1a ATGGCTGGAGGAAGTAGGGACCCTCTGGTTGTTGGTCGTGTGATAGGGGATGTGTTGGAACCCTTTGAGTGTTCCATTCCCATGAGGGTCACCTACAGTAACAGAGATGTCAGCAATGGATGTGAATTCAAACCCTCACAAGTTGTCAACCAACCAAGAATAAATATCGGTGGAGATGACCTCAGGAACTTCTACACCTTGATCGCAGTTGATCCCGATGCACCTAGTCCAAGTGACCCTAATTTGAGAGAATACCTCCATTGGCTGGTGACTGATATTCCTGCAACAACGGGACCTAGTTTCGGTCACGAGGTTGTAACGTACGAGAGTCCACGACCTATGATGGGGATTCATCGTATCGTGTTTGTGTTGTTCCGCCAACTGGGCAGGGAGACGGTGTATGCACCAGGATGGCGCCAGAATTTCAACACTAAAGAATTTGCTGAACTTTATAACCTCGGATTGCCCGTTGCGGCTGTCTATTTCAACATTCAGAGGGAATCTGGCTCTGGTGGAAGGAGGTTATAT

>Pv04_38812308_38813198_Phvul.004G119700.1_Pv_BFT ATGGAACCACTTTCTGTAGGAAGAGTGATAGGAGAAGTGGTTGACATTTTCAGCCCAAGTGTGAGAATGAATGTGACATATTCCACCAAGGAAGTTGCTAATGGTCATGAGTTAATGCCTTCTACTATTATGGCCAAGCCACGCGTGGAGATTGGTGGTGATGACATGAGAACTGCTTATACCTTGATCATGACAGACCCAGATGCTCCAAGTCCTAGTGATCCATATCTAAGGGAACATCTTCACTGGATGGTTACAGATATCCCTGGCACCACAGATGTCTCTTTTGGAAAAGAGGTTATGGGCTATGAGAGTCCAAAACCAGTGATAGGAATCCACAGATATGTGTTCATCTTGTTAAAGCAGAGAGGAAGACAAACAGTGAGACCTCCATCTTCAAGAGATCTTTTCAACACAAGGAGATTCTCGGAAGAGAATGGACTTGGCCTACCAGTTGCTGCAGTTTACTTCAATGCTCAAAGAGAGACTGCTGCAAGGAGGAGG

>Pv05_34844391_34845404_Phvul.005G124600.1_Pv_TFL1a ATGATATCAACAGATCCTCTTGTTATTGGAAGGGTGATTGGAGATGTTGTGGATCCTTTCACTACAACTCTGAAAATCACTGTCTCCTACAACAATAAGCAGGTCTACAATGGTCATGAGTTTTTTCCTTCCTCAGTAACCACTAAGCCTAAGGTTCAGATTCGTGGAGGCGACATGAGATCCTTCTTCACTCTTGTCATGACAGATCCAGACGTTCCAGGCCCTAGTGATCCATATCTCAGGGAACACTTGCACTGGATAGTCACAGACATCCCAGGCACAACGGACACCACATTTGGAAATGAGGTGGTGAACTATGAAATTCCAAGGCCAAACATAGGAATCCATAGGTTTGTGTTCCTGCTTTTCAAGCAGAAGTGCAGGCAGGCAGTGATGAAAGTACCAAGTTCCAGGGACCTCTTTAACACGAGGAGCTTTGCAGAGGAGAATGACCTGGGGCTTCCTGTGGCTGCTGTGTTTTTCAATGCTCAAAGGGAAACGGCTGCCAGAAGACGT

>Pv07_46933178_46932057_Phvul.007G229300.1_Pv_TFL1c ATGGCAAGGGTATCGACAGATCCACTAGTTATTGGGAGAGTCATAGGAGATGTTCTTGATTCTTTCACTCCAAACATAAAAATGACTGTGACTTACAGTATGAAGCAAGTCTACAATGGCAGTGAACTCTTCCCTTCCACAGTCACCACAAGGCCCAGGGTTGAGATTGGTGGAGGAGATATGAGGTCCTTCTTTACACTGATTATGACAGACCCAGATGTTCCTGGCCCTAGTGACCCTTATCTGAGAGAGCATTTGCACTGGATGGTGACAGACATTCCAGGCACAACAAATGCCTCATTTGGGAATGTGTTGGTGAGCTATGAAATGCCAAAGCCTTACATAGGGATACACAGGTTTGTGTTTGTCCTGTTCCAACAAACACGTAGGCAGTGTGTTACTCCACCCTCTTCAAGGGATCACTTTAACACTCGCAAATTCTCATCTGAGAACGATCTTGGCCTCCCTGTTGCTGCTGTCTACTTCAATGCACGGAGGGAAACTGCTGCAAGAAGACGC

>Pv08_425996_422538_Phvul.008G003700.1_Pv_FTb1a ATGCCAAGATCAACGAATCCTCTTGTTATTGGAGGTGTAATAGGTGATGTTTTGGAGCCATTTACAAGTTCTGTGTCTCTCAGAATACTCTACAACAATATTGGCTCCGAAGTTATCAACTGTTGTGAGCTCAAACCCTCACAAATCCTGAACCAACCAAGAGTTGAAATTGGTGGAGATGATCTCAGAACCCTTTACACTCTGGTGATGGTGGATCCTGATGCACCTAGTCCAGGAAATCCAAATCAGAGAGAATATCTGCACTGGTTAGTAGCTAATATTCCAGGAACCACAGGAACAAACTTCGGTGAAGAGGTTGTGGGGTATGAAGGTCCACGACCTATGATGGGGATTCATCGTATTGTGTTCATACTGTTTCGTCAACATGGTAGACAAACTGTGTATGCTCCAGGCTGGCGCCAGAATTTCAACACCAGAGATTTCAGTGAGGTTTATAACCTTGGATCACCGGTTGCAGCAACCTACTTCACCTGCAAACGACAACTTGATTATACTCGAAGGAGA

>Pv08_439251_435743_Phvul.008G003800.1_Pv_FTb2a ATGCGTGGCACAACGAACCCTCTTGTTGTAGGACGTGTAGTAGGAGATGTTCTTGAGCCCTTTGCATGCTCCATCCATCTCAGAGTTGTGTACAACAATAACAAAGAAGTGATCAATAGTGGTGAACTCAAACCCTCCCAAATAATCAACACTCCAAGAGTTGAGGTTGGTGGAGATGATCTCAGGACGCTTTACACTTTGGTCATGGTGGACCCTGATGCACCCAGCCCAAGTAACCCAAGCATGAGGGAATATCTTCACTGGTTGGTAATCAATATTCCAGCAACAACAGGTGTAAACTTTGGAGAAGAGATTGTGAGTTATGAAAGTCCAAGACCAACATCAGGGATTCATCGTCTAGTATTTGTGTTGTTTCGACAACCTGGTAGACAGTCTATTCATGCTCCTGGTTGGCGTCAAAATTTCATCACCAGAGATTTTGCTGAATATTACAATCTTGGATCACCAGTTGCTGCTGTCTATTTCAATTGCCAACGTCAAGCTGGTTCTGGAGGAAGGAGACTCATCTTA

>Vr_scaffold_7_3319591_3318322_Vr_FTc ATGGCACGAGAGGATCCTCTTTCTATTGGGGGTGTGATAGGGGATGTTCTGAACCCTTTTACAAGCTCAGTTTCTCTCACAGTTTCCATCAACAACAGAGCGATTAGCAATGGCTATGAACTGAGGCCCTCTCACGTTGCTAACCGCCCTAGAGTTACTGTAGGTGGTGAAGACCTAAGGACTTTCTATACTCTGGTCCTGGTAGATGCAGATGCACCTAGCCCTAGTAACCCCGTCTTGAGGGAATACCTTCACTGGATGGTGACAGATATTCCAGCAACCACAAATGCAAGCTTTGGTAGGGAAGTTGTGGTTTATGAGAGTCCACAACCTTCAGCAGGGATTCATCGACTTGTGTTTGTGTTGTTCCAGCAATTGGGCAGAGACACTGTAATCAGCCCAGAACTTCGCCATAATTTCAATTCAAGAAACTTTGCTGAAAATAATAACCTCACACCTGTTGCAGCAGCTTATGTCAACTGCCAAAGAGAGCGTGGCTGTGGTGGAAGGAGATAC

>Vr_scaffold_7_3334458_3332119_Vr_FTa2a ATGCCTAGCACTAGTAGGAATCCTCTTGTGGTTGGGCGTGTTATAGGGGAAGTAATAGACCCCTTTGAAAGTTCCATTCCTTTTAGGGTCTCCTATGGCAATAGAGAGGTCAACAATGGTTGTGAGCTTAAACCTTCCCAAGTTGTAAACCAACCCAGAGTGAGTGTTGGTGGAGATGATCTCAGGAACTTCTATACTCTGGTGTTAGTAGATCCTGATTCTCCCAGCCCTAGTAACCCTAATTTCAGGGAGTACCTTCATTGGTTGGTGACTGATATTCCAGCAACTACCGGGGCTAGTTTTGGTAACGAGGTTGTAAGTTATGAAAGTCCACGACCAACCATGGGGATTCATCGTTTGGTGTTTGTATTGTTCCGTCAACAGTATCGACAGAGAGTGTATGCTCCTGGATGGCGACAGAATTTCAACACCAGAGAATTTGCTGAACTTTACAACCTTGGATTGCCAGTTGCTGCAGTGTTCTTCAACTGTCAGAGGGAAACTGGTTCTGGTGGTAGGACATTT

>Vr01_17360438_17365046_Vr_FTa1a ATGCCTGGAGGAAGTAGGGACCCTCTGGTTGTTGGGCGTGTGATTGGGGATGTGTTGGACCCCTTTGAATCTTCCATTCCCATGAGGGTCTCCTACAATAACAGAGATGTCAGCAATGGATGTGAATTCAAACCTTCACATGTTTTCAACCAACCTAGAATAACTATCGGTGGAGATGATCTCAGGAACTTCTACACCTTGATCGCAGTTGATCCCGATGCACCTAGTCCAAGTGACCCTAATTTGAGAGAATACCTCCATTGGTTGGTGACTGATATTCCTGCAACAACGGGACCTAGTTTCGGTCATGAGGTTGTAACATATGAGAGTCCACGACCCATGATGGGGATTCATCGTATCGTGTTTGTGTTGTTTCGCCAACTGGGTAGGGAGACTGTGTATGCACCAGGATGGCGCCAGAATTTCAACACCAGAGAATTTGCTGAACTTTACAACCTTGGATTGCCCGTTGCGGCTGTCTATTTCAACATTCAGAGGGAATCTGGTTCTGGTGGAAGGAGATTATAT

>Vr01_36207455_36208383_Vr_BFT ATGTCTAGGCTCATGGAACCACTTTCTGTGGGAAGAGTGATAGGAGAAGTGGTTGACATTTTCAGCCCAAGTGTGAGAATGAATGTGACATATTCCACCAAGGAAGTTGCTAATGGTCATGAGTTAATGCCTTCTACTGTTATGGCCAAGCCACGCGTGGAGATTGGTGGTGATGACATGAGAACTGCTTATACCTTGATCATGACAGACCCAGATGCTCCAAGTCCTAGTGATCCATATCTAAGGGAACATCTTCACTGGATGGTTACAGATATCCCTGGCACCACAGATGTCTCTTTTGGAAAAGATATTATGGCGTATGAGAGTCCAAAACCAGTAATAGGAATCCACAGATATGTGTTCATCTTGTTCAAGCAGAGAGGAAGACAAACAGTGAGAGCTCCTTCTTCAAGAGACCATTTCAACACAAGGAGATTCTCGCAAGAGAATGGCCTTGGTCTACCAGTTGCTGCAGTTTACTTCAATGCTCAGAGAGAGACTGCTGCAAGGAGAAGG

>Vr01_4628854_4627094_Vr_MFT ATGGCTGTATCTGTCGACCCTCTTGTTGTTGGTCGGGTAATAGGTGATGTGGTCGATATGTTCGTTCCGTCAGTCAACATGTCCGTTTATTACGGTCCTAAACATGTCACAAATGGATGTGACATTAAGCCATCCGTTGCAGTCAACCCTCCTAAGCTCACTCTTACTGGAAAGAAAGAAAGCCTTTACACTCTGATTATGACAGATCCTGATGCACCAAGCCCCAGTGAACCAACCATGAGGGAATGGGTCCACTGGGTTGTGGTGGACATACCTGGTGGCACAAATCCCTTTCGTGGAAAAGAAATCCTAGCTTACACTGGACCAAAGCCACCTGTGGGAATCCATCGCTATATCTTTGTGCTCTTTGAACAGAAAGGGCCAATGGGTCCGGTGGAGCAACCAGAAAGCAGAGGAAACTTCAACACTAGGAATTTTGCTAAGGATTTGGACTTGGGTCTCCCTGTGGCCACAACCTATTTCAATGCTCAGAAGGAACCTGCTTCCAGGAGGCGTACT

>Vr03_6039142_6037894_Vr_TFL1b ATGGCAAGAATGCCTTTGGAACCTCTTATAGTGGGGAGAGTCATAGGAGAGGTTCTTGACTCTTTTACCACAACCACAAAAATGACTGTGACTTATAACAAAAAGCAAGTCTACAATGGCCATGAGTTTTTCCCTTCATCTATCAACATCAAACCCAAGGTTGAGATTGAGGGTGATGATATGAGATCCTTTTTCACACTGATCATGACAGACCCTGATGTTCCAGGCCCTAGTGACCCTTATCTGAGAGAACACTTGCACTGGATAGTGACAGACATTCCAGGCACAACAGATGCTACATTTGGGAAAGAGTTGGTGAGCTATGAGATCCCGAAACCTAATATTGGGATCCATAGGTTTGTGTTTGTCCTGTTCAAGCAAAAGCGTAGGCAGTGTGTTACTCCACCAACTTCAAGGGATCAGTTCAACACACGCAGTTTCGCAGCACAGAACGAGCTTGGGCTCCCAGTGGCTGCTGTCTACTTCAATGCACAGAGGGAAACGGCTGCAAGAAGACGC

>Vr04_20473091_20475526_Vr_FTb2a ATGCGTGGAACAACGAACCCTCTTGTTGTTGGACGTGTAATAGGAGATGTTGTGGAACCATTTGCATGTTCCATTCCTCTGAGAGTTGTCTACAACCATAACAAAGAAGTCATCAACAGTGGTGAGCTCAAACCCTCCCAAATAGTCTCCCATCCAAGAGTTGAGGTTGGCGGAGATGATCTCAGGACCCTTTACACTTTGGTCATGGTGGACCCTGATGCACCCAGCCCAAGTAACCCAACTATGAGGGAATATCTTCACTGGTTGGTAATCAACATTCCAGCGACAACAGGGTCAAACTTTGGAGAAGAGATTGTGAGTTATGAAAGTCCAAGACCAACATCAGGGATTCATCGTTTAGTATTCGTGTTGTTTAAACAACCTGGTAGACAATCGATACATGCTCCTGGGTGGCGTCAAAATTTCATCACCAGAGATTTCGCCGAATATTACAATCTTGGATCACCAGTTGCTGCTGTCTATTTCAATTGCCAGCGTCAATCTGGTTCTGGTGGAAGGAGGCTCATCTTA

>Vr04_20486238_20489313_Vr_FTb1c ATGGAGGGTCCCCTTGTAATTGGACGTATAATAGGAGAAGTTTTGGATCCATTTACGAGTTCAGTGAGTCTGAGAGTTGTTTATAAGAATCAGACAGAAGTTATCAATAGTTGTGAGTTGAAACCTTCACAAATTGTGAACAAACCAAGAGTTCATATTGGTGGAGATGACCTAAGAGTCTTTTACACTCTGATAATGGTCAATCCTGATGCTCCTAGCCCTAGTCACCCTTCTATGAAGGAATATTTGCATTGGTTGGTAACCAATATTCCAGCTACTACTGCGGCAAGCTTTGGACATGAAATCGTGGAATATGAAAGTCCACGACCAACGTCGGGGATTCATCGTATTGTTTTGGTGTTGTTTCGACAATTGGGTAGACAAATAGTGCATGCTCCAAGATGGCGTAACAATTTCAACACCAAAGATTTTGCTCAAGTTTACAACCTTGGATTACCAGTTGCTGCTGTCTATTTCAACTGTCAACGTGAAAGCGGTTGGGGTGGAAGAAGGGCG

>Vr04_20495482_20497501_Vr_FTb1b ATGCCTAGATCAACAGATCCTCTTGTTATAGGAGGTGTAATAGGTGATGTTTTGGAGCCATTTACAAGTTCTGTTTCTCTCAGGATACTTTACAACAATTCCTTTCAAGTTATCAACTGTTGTGAGCTCAAACCCTCACAAGTACTAAACCAACCAAGAGTTGAAATTGGTGGAGATGATTTCAGAACCCTTTACTCTCTGGTGATGGTGGATCCTGATGCACCTAGTCCAGGAAATCCAAATCAGAGGGAATATCTGCACTGGTTAGTAGCCAATATTCCAGGAACTACAGGAACAAACTTCGGTGAAGAGGTTGTGGGATATGAAGGTCCACGACCTATGATGGGGATTCATCGTATTATTTTCGTACTTTTTCGGCAACTGGGTAGACAAACCGTATACGCTCCAGGATGGCGACAAAATTTCAACACCAGAGATTTTAGTGAACTTTACAATCTTGGATCACCAGTTGCAGCATCCTACTTCAACTGCAAA

>Vr04_20505855_20507302_Vr_FTb1a ATGCCTAGATCAACAGATCCTCTTGTTATAGGAGGTGTAATAGGTGATGTTTTGGAGCCATTTACAAGTTCTGTTTCTCTCAGGATACTTTACAACAATTCCTTTCAAGTTATCAACTGTTGTGAGCTCAAACCCTCACAAGTACTAAACCAACCAAGAGTTGAAATTGGTGGAGATGATTTCAGAACCCTTTACTCTCTGGTGATGGTGGATCCTGATGCACCTAGTCCAGGAAATCCAAATCAGAGGGAATATCTGCACTGGTTAGTAGCCAATATTCCAGGAACTACAGGAACAAACTTCGGCGAAGAGGTTGTCGGGTACGAAGGTCCACGACCAGTGATGGGGATTCATCGTATTGTTTTCATACTGTTTCGTCAACCTAGCAGACAAACTGTGTACGCTCCAGGATGGCGACAAAATTTCAACACCAGAGATTTTAGTGAGCTTTATAATCTTGGATCACCAGTTGCAGCAGCCTACTTCAACTGCAAACGACAACTTGATTCTACTCGAAGGAGA

>Vr04_7141382_7140392_Vr_TFL1a ATGAATATGATATCAACAGACCCTCTTGTGATTGGGAGGGTGATCGGAGATGTTGTGGATCTTTTCTCTCCAACTGTGAAAATCACTGTCTCCTACAACAACAATAAGCAGGTCTATAACGGTCATGAGTTTTTCCCTTCCTCAGTAGCCAACAAGCCTAAGGTTCAGATTCGTGGAGGCGACATGAGATCCTTCTTCACTCTTCTCATGACAGATCCAGACGTTCCTGGCCCTAGTGATCCATATCTCAGGGAACATTTACACTGGATAGTCACGGACATCCCTGGCACAACGGACACCACATTTGGAAATGAGGTGGTGAGCTATGAAATTCCAAGGCCAAACATAGGCATCCATAGGTTTGTGTTCCTCCTTTTCAAGCAGAAGTGCAGGCAGGCAGTGATGAAAATACCAAGTTCTAGGGACCTCTTCAAGACGAGAAGCTTTGCAGAGGAGAATGACCTTGGGCTTCCTGTGGCTGCTGTGTTTTTCAATGCTCAAAGGGAAACGGCTGCCAGAAGACGT

>Vr08_11746865_11745848_Vr_TFL1c ATGGCAAGGGTGTCCACAGATCCACTAGTTATTGGGAGAGTCATAGGAGATGTTCTTGATTCTTTCACTCCAAGCACAAAAATGACTGTAACATACAGTAAGAAGCAAGTCCACAATGGCCATGAACTCATTCCTTCCACAGTTACCACATGCCCAAGGGTTGAGATTGGTGGAGGAGATTTGAGATCCTTCTTTACACTGATTATGACAGACCCTGATGTCCCAGGACCTAGTGACCCTTATCTGAGAGAACATCTGCACTGGATGGTGACAGACATACCAGGCACAACAAATGCCTCATTTGGGAATGTGTTGGTGAGCTATGAAATGCCAAAGCCTAACATAGGGATACACAGGTTTGTGTTTGTCCTGTTCAAGCAAAAACGTAGGCAGTGTGTTACTCCACCCTCTTCGAGGGACAACTTTAACACTCGAAAATTCTCATCTGAGAACGATCTTGGCCTCCCTGTTGCTGCTGTCTACTTCAATGCACAGAGGGAAACTGCTGCAAGAAGACGC
