# Supplementary material for: Expansion of the phosphatidylethanolamine binding protein family in legumes: a case study of Lupinus angustifolius L. FLOWERING LOCUS T homologs, LanFTc1 and LanFTc2
Source: BMC Genomics. 2016 Oct 21;17:820. doi: 10.1186/s12864-016-3150-z (PMC5073747; doi:10.1186/s12864-016-3150-z)
Supplement: Additional file 11: — Settings applied to perform MrBayes inference of phylogeny. (DOC 31 kb) [file 12864_2016_3150_MOESM11_ESM.doc]

**Settings applied to perform MrBayes inference of phylogeny**

| Substitution model | Codon (M1) |
| --- | --- |
| Rate variation | Gamma |
| Outgroup sequence | AtBFT.AT5G62040 |
| Gamma categories | 5 |
| Chain length | 2500000 |
| Subsampling frequency | 400 |
| Burn-in length | 400000 |
| Nchains | 5 |
| Heated chain temperature | 0.25 |
| Unconstrained branch lengths | exponential 10 |
| Shape parameter | exponential 10 |
